# Highly stable and self-repairing membrane-mimetic 2D nanomaterials assembled from lipid-like peptoids

**DOI:** 10.1038/ncomms12252

**Published:** 2016-07-12

**Authors:** Haibao Jin, Fang Jiao, Michael D. Daily, Yulin Chen, Feng Yan, Yan-Huai Ding, Xin Zhang, Ellen J. Robertson, Marcel D. Baer, Chun-Long Chen

**Affiliations:** 1 Division of Physical Sciences, Pacific Northwest National Laboratory, Richland, Washington 99352, USA; 2 School of Chemistry and Molecular Engineering, East China Normal University, Shanghai 200241, China; 3 College of Chemistry and Chemical Engineering, Linyi University, Linyi, Shandong 276005, China; 4 Institute of Rheology Mechanics, Xiangtan University, Xiangtan, Hunan 411105, China; 5 Molecular Foundry, Lawrence Berkeley National Laboratory, Berkeley, California 94720, USA

## Abstract

An ability to develop sequence-defined synthetic polymers that both mimic lipid amphiphilicity for self-assembly of highly stable membrane-mimetic 2D nanomaterials and exhibit protein-like functionality would revolutionize the development of biomimetic membranes. Here we report the assembly of lipid-like peptoids into highly stable, crystalline, free-standing and self-repairing membrane-mimetic 2D nanomaterials through a facile crystallization process. Both experimental and molecular dynamics simulation results show that peptoids assemble into membranes through an anisotropic formation process. We further demonstrated the use of peptoid membranes as a robust platform to incorporate and pattern functional objects through large side-chain diversity and/or co-crystallization approaches. Similar to lipid membranes, peptoid membranes exhibit changes in thickness upon exposure to external stimuli; they can coat surfaces in single layers and self-repair. We anticipate that this new class of membrane-mimetic 2D nanomaterials will provide a robust matrix for development of biomimetic membranes tailored to specific applications.

## Abstract (web summary)

Biomimetic membranes can be used for various applications such as sensors and separations. Here, Chen *et al*. report the assembly of lipid-like peptoids into stable and self-repairing 2D membrane nanomaterials that change in thickness when under external stimuli.

## Graphical abstract

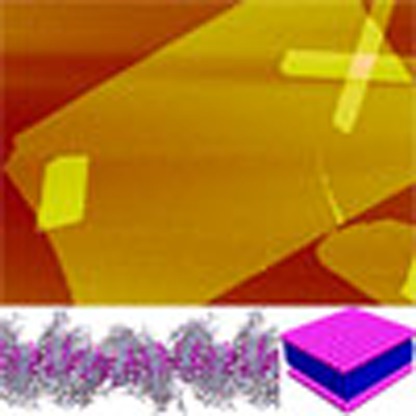

Molecular self-assembly is the key to building up well-defined functional structures in biology^1^,^2^. For example, self-assembly of lipid molecules and membrane proteins is crucial in defining cell architecture and enabling cell function^2^. From a material's perspective, cell membranes, which consist of 3–5-nm-thick lipid bilayers, are of particular interest because they represent a class of two-dimensional (2D) nanomaterials that have rather unusual properties, such as sequence-specific water and ion transport and the ability to self-repair^2^,^3^. Because cell membranes are a great source of inspiration in biotechnology and nanotechnology, many efforts have been made to create synthetic, membrane-mimetic 2D nanomaterials assembled from lipids, or synthetic analogues of lipids (for example, lipid-like peptides, block copolymers)^3^,^4^,^5^. However, assembly of these lipid analogues into stable, free-standing, bilayer 2D membranes presents a significant challenge, and one of the major disadvantages that limit the technological applications of the lipid-based membranes is their poor stability^3^,^4^,^5^,^6^.

Peptoids (or poly-N-substituted glycines)^7^, non-natural mimetics of peptides and proteins, have received particular attention because they can be cheaply and efficiently synthesized, and several hundred commercially available amines can be used to attain large side-chain diversity^8^. Peptoids are highly peptidase/proteinase-resistant^9^, and are chemically and thermally stable^7^. Because peptoids lack backbone hydrogen bonds, which simplifies tuning interpeptoid and peptoid-surface interactions exclusively through side-chain chemistry, they offer unique opportunity for achieving sequence-specific molecular recognition^7^,^10^ and controlling self-assembly^11^,^12^,^13^,^14^,^15^. All these features make peptoids highly attractive as synthetic analogues of lipids for self-assembly of membrane-mimetic 2D materials. Herein we report the first example of self-assembling highly stable and self-repairing membrane-mimetic 2D nanomaterials by designing lipid-like sequence-defined peptoids.

## Results

### Design of lipid-like peptoids

**Figure 1:**
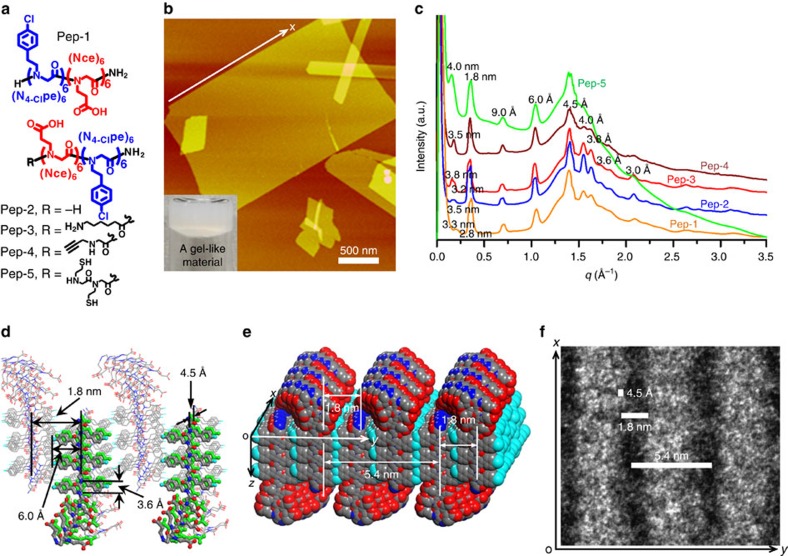
Self-assembly of lipid-like peptoids into highly stable and crystalline membrane-mimetic 2D nanomaterials. (**a**) Structures of Pep**-1**–Pep**-5**. (**b**) AFM image of self-assembled Pep**-1** membranes. The insert is the optical image of the gel-like material containing a large number of free-standing Pep-**1** nanomembranes. (**c**) X-ray diffraction data of membranes assembled from Pep-**1** to Pep-**5**. (**d**) Molecular model showing proposed packing of Pep-**2** inside membranes, in which the peptoid backbone to backbone distance is 4.5 Å along the *x* direction and is 1.8 nm along the *y* direction. (**e**) Space-filling molecular model of Pep-**2** membranes showing formation of strips of hydrophilic domains along the *x* direction on membrane surface. (**f**) A high-resolution TEM image showing well-aligned strips in the Pep-**5** membrane structure.

Inspired by the diblock-like feature of lipids and taking advantage of the ease of peptoid design, here we designed and synthesized a series of amphiphilic, lipid-like peptoids containing two sub-blocks, respectively, six polar residues, N-(2-carboxyethyl)glycine (Nce) and six nonpolar residues, N-[2-(4-chlorophenyl)ethyl]glycines(N_4-Cl_pe) (Pep-**1**–Pep-**5**, Fig. 1a, see Supplementary Methods for details). In doing so, our goal was to build lipid-bilayer-like membrane-mimetic 2D nanomaterials by using the six Nce groups to mimic polar lipid head groups and the six N_4-Cl_pe groups to create aromatic counterparts of nonpolar lipid tails. Moreover, the aromatic residue N_4-Cl_pe was chosen because it is known to stabilize self-assembled materials by enhancing intermolecular packing through, for example, *π*–*π* interactions^16^.

### Assembly of lipid-like peptoids into 2D membranes

Peptoid membranes were formed through an easy, evaporation-induced crystallization process from amorphous to highly crystalline materials (see Supplementary Methods and Supplementary Figs 1 and 2 for details). Atomic force microscopy (AFM) studies showed that these peptoids formed 2D membrane-like nanomaterials with straight edges (Fig. 1b and Supplementary Figs 1b and 3c). Membranes assembled from Pep-**1** to Pep-**5** exhibited a thickness in the range of 3.5–4 nm (Supplementary Figs 1b and 3c), similar to the thickness of lipid membranes^3^. The observation of many overlapping membranes (Supplementary Fig. 1b,c) indicated that they were free-standing in solution and overlapped during sample preparation.

X-ray analyses demonstrate that membranes assembled from Pep-**1** to Pep-**5** are highly crystalline and all exhibit similar X-ray diffraction patterns, with only noticeable differences in the first low *q* peak corresponding to membrane thickness (Fig. 1c). The difference and broadening in this peak suggests that peptoid backbones are dynamic and the thickness of peptoid membranes is variable (Fig. 1c). The similarity in other X-ray diffraction peaks indicates that functional groups, such as clickable alkynyl group in Pep-**4**, can be added at the N termini of membrane-forming peptoids to build functional membranes with similar structures.

For the peaks above *q*=0.25 Å^−1^, the strongest peak at *q*=1.4 Å^−1^ is because of the alignment of peptoid chains, which leads to a spacing of 4.5 Å between the peptoids^11^. The 3.6-Å spacing is the distance between each residue along the chain direction. The 6-Å spacing corresponds to the N_4-Cl_pe side-chain length (Fig. 1d). The 1.8-nm spacing corresponds to the distance between two peptoid backbones packed inside the membranes with N_4-Cl_pe groups facing each other (Fig. 1d,e). The presence of extensive π-stacking is evidenced by X-ray diffraction peaks at 4, 3.8 and 3 Å (refs 16, 17).

In addition, the hydrophobic interactions that impart high stability to the membrane are robust to variations in ionic strength and pH. Pep-**3** nanomembranes formed even when 500 mM NaCl was added to the crystallization solution (Supplementary Fig. 4a), and Pep-**2** membranes formed at pH values from 5.6 to 10.5 (Supplementary Fig. 4b–e). To further illuminate the role of hydrophobic interactions in membrane formation, we designed peptoids Pep-**6** and Pep**-7** by varying the position of the side-chain chloro (-Cl) group on the nonpolar residues. Pep-**6**, which has six N-[2-(3-chlorophenyl)ethyl]glycines (N_3-Cl_pe) residues, formed membranes with slightly different packing (Supplementary Fig. 5), while Pep**-7** with six N-[2-(2-chlorophenyl)ethyl]glycines (N_2-Cl_pe) did not self-assemble into crystalline membranes (Supplementary Fig. 5), which N_2-Cl_pe might create too much steric hindrance to maintain an ordered packing of hydrophobic domains. These results demonstrate that the position of the -Cl group is important for the packing of hydrophobic domains and the resultant membrane structures.

To further demonstrate that the ordering of hydrophobic domains is the key to forming membrane structures, we synthesized peptoids Pep-**8**–Pep-**11** by either having only three N_4-Cl_pe residues as hydrophobic domains (Pep-**8**) or changing the polar domains from Nce_6_ to Nce_3_ (Pep-**9**), non-carboxyl-containing Nte_3_ [Nte=N-2-(2-(2-methoxyethoxy)ethoxy)ethylglycine] (Pep-**10**) or (NceNae)_3_ [Nae=N-(2-aminoethyl)glycine] (Pep-**11**; Supplementary Fig. 6). As we expected, Pep-**8** with only three N_4-Cl_pe residues could not form membranes because of the lack of enough hydrophobic interactions to stabilize the membrane structure. On the other hand, when six N_4-Cl_pe residues remain, Pep-**9**–Pep-**11** with even three polar residues (Nce_3_ of Pep-**9** and Nte_3_ of Pep-**10**) or a mixture of Nce and Nae residues (Pep-**11**) self-assembled into biomimetic membranes (Supplementary Fig. 6).

To highlight the significance of hydrophobic interactions in the membrane formation and the similarity of designed peptoids and lipids, we designed and synthesized peptoids Pep-**12**–Pep-**14**, which have various number of Nhex [Nhex=*N*-(6-hexyl)glycine] residues (Supplementary Fig. 7). While Pep-**12**, an addition of two Nhex residues at the C terminus of Pep-**2**, self-assembled into biomimetic membranes with ∼4 nm thickness (Supplementary Fig. 7a), replacing of all aromatic N_4-Cl_pe residues with aliphatic Nhex residues resulted in no membrane formation (Supplementary Fig. 7b). We reason that such changes might result in forming decreased hydrophobic interactions because of the loss of *π–π* interactions^16^. To increase the hydrophobic interactions for sufficiently stabilizing membrane structures, we further synthesized Pep-**14**, which has 12 Nhex residues and 6 Nce residues. As we expected, Pep-**14** self-assembled into ∼3.8-nm-thick biomimetic membranes (Supplementary Fig. 7c).

The presence of 4.5 Å and 1.8-nm spacings (Fig. 1c) in Pep-**5** membranes was also observed within their high-resolution transmission electron microscopy (TEM) images (Fig. 1f and Supplementary Fig. 3a,b). These TEM data showed that Pep-**5** formed well-aligned strips along the *x* direction (defined as the direction along straight edge, Fig. 1f and Supplementary Fig. 3a,b) in the membrane structure, suggesting peptoids packed anisotropically and assembled into membranes with a faster rate along the *x* direction than along the *y* direction. Indeed, time-dependent TEM experiments, which showed that Pep-**3** self-assembled into elongated nanoribbons as intermediates, further confirmed this anisotropic membrane formation process (Supplementary Fig. 8). We infer that peptoids align along the *x* direction with stronger interpeptoid interactions than those along the *y* direction; however, interpeptoid interactions along both directions are strong enough for peptoids to build membrane-mimetic 2D nanomaterials.

These AFM, X-ray diffraction and TEM results enable us to propose a structural model for peptoid membranes (Fig. 1). Membrane thickness is determined by the hydrophobic N_4-Cl_pe domains and the flexible hydrophilic Nce domains. To minimize the exposure of hydrophobic groups to water and to bring carboxyl groups on adjacent peptoid chains next to each other, the flexible hydrophilic domains need to be floppy, but they could align laterally to form strips along the *x* direction (Fig. 1e). Amphiphilic peptoids are stacked together along both *x*- and *y* directions to form a lipid-membrane-like structure consisting of a hydrophobic interior and two polar faces. We reason that the significant hydrophobic interactions between N_4-Cl_Pe groups and the diblock-like features of these assembling peptoids combine to create strong interpeptoid interactions and drive the crystallization of free-standing membranes in solution. Thus, these peptoids exhibit much more robust assembly than hydrocarbon-based phospholipids, which self-assemble into liposomes or micelles in solution and only form bilayer 2D membranes at interfaces^3^,^18^.

### Molecular dynamic simulations of peptoid membranes

**Figure 2:**
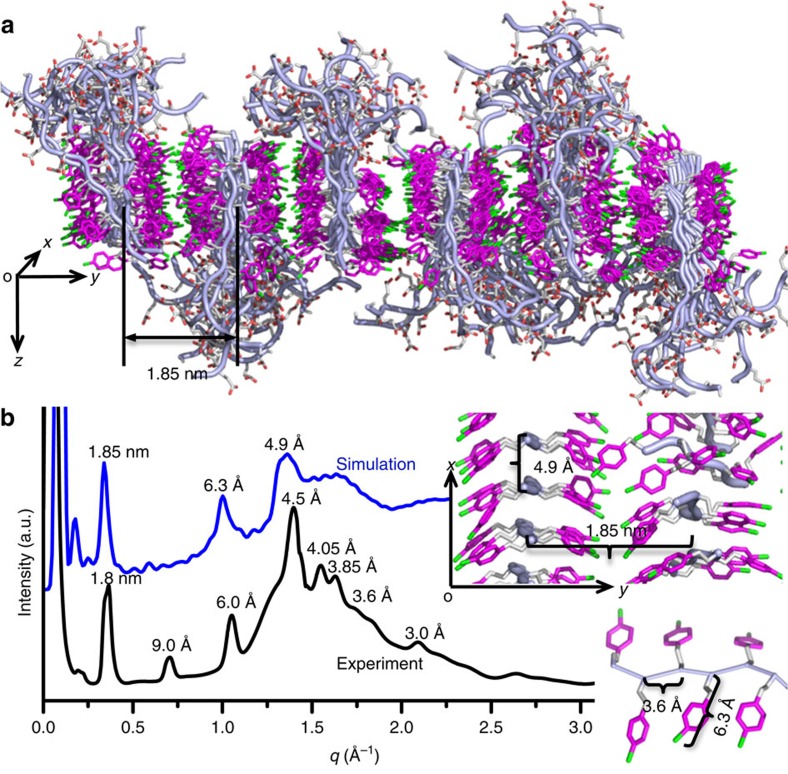
Molecular dynamics simulation of a structural model for peptoid nanomembranes. (**a**) Final structure from a 600-ns simulation of a 16 × 6 lattice of the lipid-like Pep-**1**. Carboxyl residues were in the protonated state, and hydrophilic domains formed strips along the *x* direction; the middle right panel shows the simplified membrane surface with hydrophilic domains deleted, which shows the anisotropic packing of the N_4-Cl_pe domains of Pep-**1** along *x*- and *y* directions; the bottom right panel shows a hydrophobic domain of Pep-**1** showing the side chain to side chain distance. (**b**) Simulated X-ray diffraction data for the Pep-**1** membrane agrees well with the experimental data.

To test this model and to understand the interactions that stabilize a robust assembly, we used molecular dynamics simulations to investigate the stability and flexibility of these peptoid membranes. Individual peptoids were initially placed in extended conformations and the N_4-Cl_Pe residues were positioned in the membrane centre to minimize the exposed hydrophobic surface area. To model the peptoid membrane atomistically, we set up a periodic box of 16 × 6 Pep-**1** (6.4 × 10.2 nm) with half the peptoids oriented up along the *z* axis and half oriented down. The side chains were oriented along the *y* axis, and the peptoids were stacked along the *x* axis. The system was solvated with 2 nm of explicit SPC water in the *z* direction. Peptoids were modelled with the AMBER03 forcefield^19^ with parameters from the generalized amber force field^20^,^21^, and the simulations were carried out for 600 ns using GROMACS 4.6.4 (ref. 22). Our simulated structures in Fig. 2 showed extensive *π*-stacking in the hydrophobic N_4-Cl_Pe region and disorder in the carboxyl tails. In addition, simulated X-ray diffraction data for Pep-**1** membranes is in good agreement with the experimental data (Fig. 2b). The observation of strips of flexible hydrophilic domains (Fig. 2 and Supplementary Fig. 9) agrees well with TEM data (Fig. 1f and Supplementary Fig. 3a,b). Furthermore, simulated structures showed much higher contact density along the *x* direction (15.9 contacts per nm^2^ of contact area) than the *y* direction (0.86 contacts per nm^2^ of contact area); this indicates that peptoid packing in the membrane is anisotropic, which is consistent with our experimental results showing the anisotropic membrane formation process (Supplementary Fig. 8).

### Incorporating functional objects into peptoid membranes

**Figure 3:**
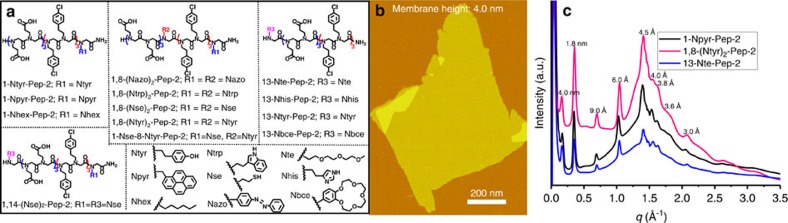
Tolerance of peptoid membrane structures to incorporation of functional objects. (**a**) Membrane-forming peptoids containing a wide range of functional objects as side chains at a number of locations, in which nine functional objects were used and listed. (**b**) AFM image of one 2D nanomembrane assembled from 1-Npyr-**Pep-2**. (**c**) X-ray diffraction data of membranes assembled from 1-Npyr-**Pep-2**, 1,8-(Ntyr)_2_-**Pep-2** and 13-Nte-**Pep-2** are similar to the X-ray diffraction data of Pep-**2**, which shows that the core structure is retained.

While peptoids Pep-**1**–Pep-**5** have a stable membrane-mimetic core structure, we also aimed to design membranes that can incorporate different functional objects to perform useful chemical functions while retaining the high stabilities and core structures of the parent peptoids. To test our membranes for this property, we synthesized 13 peptoids based on modifying Pep-**2** by incorporating functional objects as side chains either at the terminus, or inserted into strategic locations (Fig. 3, also see Supplementary Methods and Supplementary Figs 10–23 for details). These functional objects include N-[2-(1H-indol-3-yl)ethyl]glycine (Ntrp) that mimics tryptophan—a redox-active residue that facilitates electron transfer^23^; N-[2-(4-hydroxylphenyl)ethyl]glycines (Ntyr) that mimics tyrosine—a redox-active residue significant for electron transfer during photosynthesis in nature^24^; [2-(4-imidazolyl)ethylamine]glycines (Nhis) that mimics histidine—a residue significant for carbonic anhydrases to capture CO_2_ (ref. 25); *N*-(2-thiolethyl)glycine (Nse) that mimics cysteine^26^; azo-containing ultraviolet-responsive^27^ *N*-[4-(2-phenyldiazenyl)phenyl]glycines (Nazo); and *N*-[benzo-15-crown-5-ether]glycines (Nbce) that could build ion channels^28^,^29^. Hydrophilic Nte and hydrophobic N-[(1-pyrenemethyl)]glycines (Npyr) and Nhex were also used to test the tolerance of peptoid membrane assembly in incorporating highly hydrophilic and hydrophobic objects.

As we expected, AFM, TEM, scanning electron microscopy (SEM) and X-ray diffraction results showed that all these peptoids formed 2D nanomembranes with similar structures to Pep-**2** (Fig. 3 and Supplementary Figs 10–23). As shown in Fig. 3, both peptoids 13-Nte-**Pep-2**, where Nte is at the N terminus of the peptoid as the thirteenth side chain, and 1-Npyr-**Pep-2**, where Npyr is located at the C terminus as the first side chain), formed 2D membranes. X-ray diffraction results confirmed that they have highly crystalline structures similar to Pep-**2** (Fig. 3c). The consistency of all membrane structures suggests that membrane assembly is robust to tolerate the addition of functional objects as peptoid side chains at various locations, building multicomponent complexity and offering protein-like functionality.

**Figure 4:**
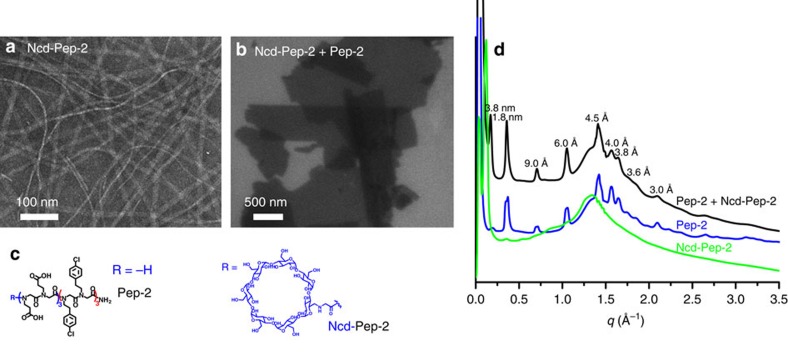
Incorporating functional objects through co-crystallization. (**a**) A TEM image showing 1D nanoribbons from N_cyclodextryl_ (Ncd)-**Pep-2**. (**b**) A SEM image showing 2D membranes co-assembled from **Pep-2** and Ncd-**Pep-2** (molar ratio 1:4). (**c**) Structures of **Pep-2** and Ncd-**Pep**-**2**. (**d**) X-ray diffraction data of membranes assembled from Ncd-**Pep-2**, **Pep-2** or the mixture of Ncd-**Pep-2** and **Pep-2**; membranes assembled from the mixture of Ncd-**Pep-2** and **Pep-2** (molar ratio 1:4) exhibit similar X-ray diffraction patterns to those of Pep-**2**, showing structural similarity.

To test whether functional objects can be introduced via co-crystallization, **Pep-2** containing Ncd (namely Ncd-**Pep-2**; Fig. 4), which itself self-assembles into one-dimensional (1D) nanoribbons (Fig. 4a), was mixed with membrane-forming **Pep-2** in a 1:4 molar ratio. SEM results showed that the peptoid mixture formed only 2D membranes (Fig. 4b), and X-ray diffraction data show that these nanomembranes retain the core structure of the **Pep-2** membrane (Fig. 4d). Furthermore, the density of Ncd residues can be controlled by varying the molar ratios of Ncd-**Pep-2** and **Pep-2** (Supplementary Fig. 24).

### Peptoid membranes are highly stable and dynamic

**Figure 5:**
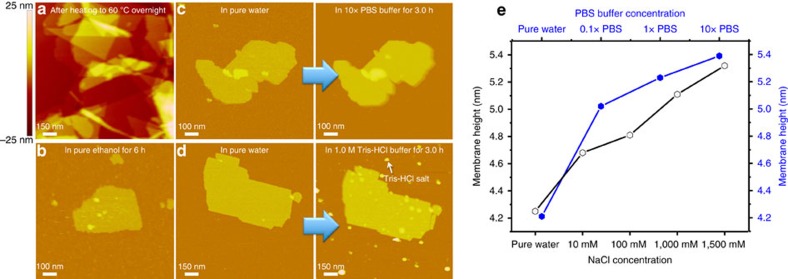
Peptoid membranes are highly stable and dynamic. (**a**) *Ex situ* AFM image of Pep-**3** membranes after heating to 60 °C overnight in water solution. (**b**) *In situ* AFM images showing Pep-**3** membranes even survive after being exposed to pure ethanol solution for 6 h. (**c**) *In situ* AFM images showing Pep-**3** membranes are stable in 10 × PBS buffer (pH 7.4) for 3 h. (**d**) *In situ* AFM images showing Pep-**3** membranes are stable in 1 M Tris buffer (pH 7.4) solution for 3 h. (**e**) Pep-**3** membranes exhibit increases in thickness proportional to the NaCl or PBS buffer concentration.

To test the stability of peptoid membranes, we exposed them to a range of solvents as well as high temperature. As shown in Fig. 5, peptoid membranes survived when they were placed in a mixture of water and organic solvents, or even in pure CH_3_CN (Supplementary Fig. 25) and EtOH (Fig. 5b) for over 6 h. They were also stable in 10 × PBS buffer (pH 7.4; Fig. 5c), 1 M Tris-HCl buffer (pH 7.4; Fig. 5d) and 1.5 M NaCl (Supplementary Fig. 26), or after heating to 60 °C in water overnight (Fig. 5a).

Next, we investigated whether these membranes would exhibit salt-induced thickness changes, as is observed with lipid bilayers^30^,^31^. *In situ* AFM images showed that when the peptoid membranes were exposed to NaCl solution or PBS buffers of increasing concentration, their thicknesses increased by ∼30% from ∼4.2 to ∼5.4 nm (Fig. 5e and Supplementary Fig. 26).

### Spontaneous assembly of peptoid membranes at interfaces

**Figure 6:**
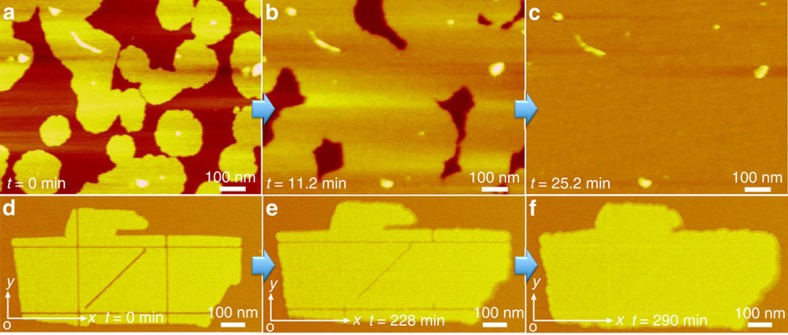
Formation of a single-layer continuous membrane on mica and membrane self-repair. (**a**–**c**) *in situ* AFM images at different time points showing formation of single-layer continuous Pep-**3** membranes on mica through a process of heterogeneous nucleation and lateral growth. (**d**–**f**) *In situ* AFM images at different time points showing the anisotropic self-repair of one Pep-**3** membrane with mechanically induced defects along the *x*, *y* and 45° to *x* and *y* directions, in which the Pep-**3** membrane exhibited the most rapid repair along the *x* direction.

To examine whether lipid-like peptoids would also form single-layer membranes on solid substrates, as is the case with phospholipid membranes, mica surfaces were exposed to a 93.5-μM aqueous solution of Pep-**3** (Supplementary Methods). *In situ* AFM imaging showed that Pep-**3** self-assembled into a continuous single-layer membrane covering the entire mica surface through heterogeneous nucleation of islands followed by lateral spreading and fusion (Fig. 6a–c). The measured thickness of ∼3.5 nm indicates that Pep-**3** forms similar membranes both in solution and on substrates.

### Peptoid membrane self-repairs

One remarkable property of cell membranes is their ability to self-repair^32^. To test whether peptoid membranes can self-repair, we deposited free-standing Pep-**3** membranes on a mica surface. The AFM tip was used to mechanically create defects along the membrane *x*- and *y* axes and at 45° to the *x*- and *y* axes (Fig. 6d). The resulting defect-containing membrane was then incubated in a 10-μM aqueous solution of Pep-**3** (Supplementary Methods). As shown in Fig. 6f, the defects were completely repaired after 290 min. Moreover, the membrane self-repaired most rapidly along its *x* axis and most slowly along its *y* axis, which provides further evidence of faster assembly in the *x*- than in the *y* direction. This is consistent with our proposed 2D membrane model (Fig. 1d,e), time-dependent mechanistic TEM studies (Supplementary Fig. 8) and molecular dynamics simulations (Fig. 2 and Supplementary Fig. 9).

### Nanoscale patterning of functional objects within membranes

Interestingly, Pep-**3** membranes can also be repaired by addition of Ncd-**Pep-2** or N_NHS-Rhodamine_-Pep**-3** monomers, which indicates that the co-crystallization process can be used not only to repair defect-containing membranes but also to introduce functional objects into membranes in a desired pattern (Supplementary Figs 27 and 28). By taking advantage of this co-crystallization capability, which is not available in previous peptoid 2D materials^33^, we have developed an approach to introduce functional objects into assembled 2D materials to create nanoscale patterning.

## Discussion

We have demonstrated the self-assembly of lipid-like sequence-defined peptoids into a new class of membrane-mimetic 2D materials that are highly stable and are capable of self-repair. They self-assemble either as free-standing membranes through an anisotropic crystallization process in solution or as continuous single-layer membranes on substrate surfaces through a heterogeneous nucleation process. They exhibit a number of properties associated with cell membranes, including hydrophobic cores and hydrophilic surfaces, thicknesses in the 3.5–5.6-nm range, spontaneous assembly at interfaces, thickness variations in response to changes in Na^+^ concentration and the ability to self-repair. We further demonstrate that peptoid membranes provide a robust platform to incorporate and pattern a diverse range of functional objects as peptoid side chains either before assembly or by co-crystallization approaches. We are currently using these approaches to incorporate functional objects such as artificial channels^34^,^35^,^36^ and protein complexes^4^,^35^. We believe that our membrane-mimetic 2D materials represent a significant step in the development of biomimetic membranes with possible applications in water purification, surface coatings, biosensing, energy conversion and biocatalysis.

## Methods

Detailed information on materials and methods is available in the Supplementary Methods. Peptoids were synthesized using a modified solid-phase submonomer synthesis method as described previously^8^,^10^. They were either synthesized on a commercial Aapptec Apex 396 robotic synthesizer or manually synthesized in a 6-ml plastic vial. Peptoids were cleaved from the resin by addition of 95% trifluoroacetic acid in water, and then dissolved in water and acetonitrile (*v*/*v*=1:1) for HPLC purification. Lyophilized and HPLC-grade peptoids were dissolved in the mixture of water and acetonitrile (*v*/*v*=1:1) to make a 5-mM clear solution, which was then transferred to a 4°C refrigerator for slow evaporation. Suspensions or gel-like materials containing a large amount of crystalline membranes were formed after a few days.

### Data availability

All relevant data are available from the authors.

## Additional information

**How to cite this article:** Jin, H. *et al*. Highly stable and self-repairing membrane-mimetic 2D nanomaterials assembled from lipid-like peptoids. *Nat. Commun.* 7:12252 doi: 10.1038/ncomms12252 (2016).

## Supplementary Material

Supplementary Information Supplementary Figures 1-57, Supplementary Methods and Supplementary References
