# Supplementary material for: Highly stable and self-repairing membrane-mimetic 2D nanomaterials assembled from lipid-like peptoids
Source: Nat Commun. 2016 Jul 12;7:12252. doi: 10.1038/ncomms12252 (PMC4945955; doi:10.1038/ncomms12252)
Supplement: Supplementary Information — Supplementary Figures 1-57, Supplementary Methods and Supplementary References [file ncomms12252-s1.pdf]

# Supplementary Figures

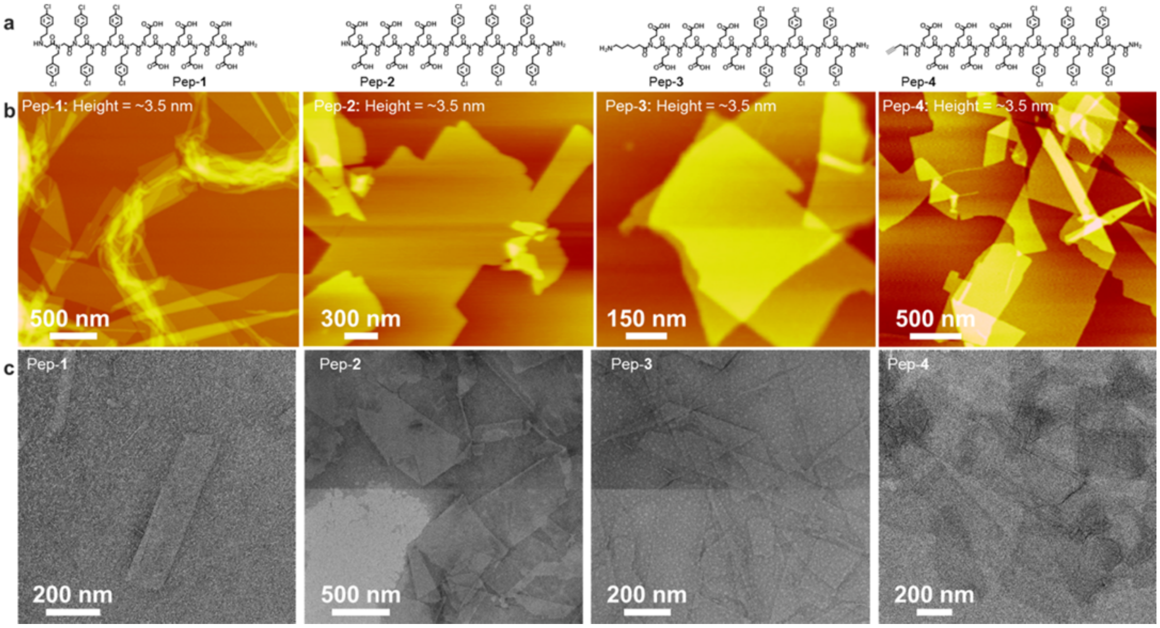


**Supplementary Figure 1 | 2D membranes assembled from lipid-like peptoids. a**, Structures of membrane-forming peptoids Pep-**1** – Pep-**4**. **b**, AFM images of biomimetic membranes assembled from Pep-**1**, Pep-**2**, Pep-**3**, and Pep-**4**. These membranes exhibit a similar height of ~3.5 nm. **c**, TEM images of biomimetic membranes assembled from Pep-**1**, Pep-**2**, Pep-**3**, and Pep-**4**; 2% phosphotungstic acid was used for negative staining.

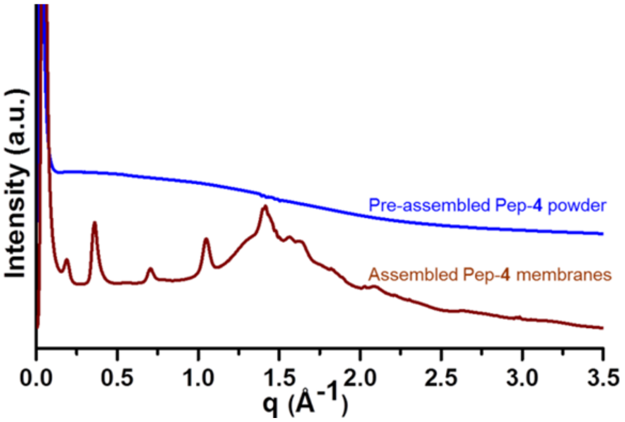


**Supplementary Figure 2 | XRD data of** Pep-**4 membranes and pre-assembled** Pep-**4 powder.** XRD data show that pre-assembled Pep-**4** lyophilized from its $H_2O$ and $CH_3CN$ (1:1) solution is amorphous before they are crystallized into highly-ordered membrane structures.

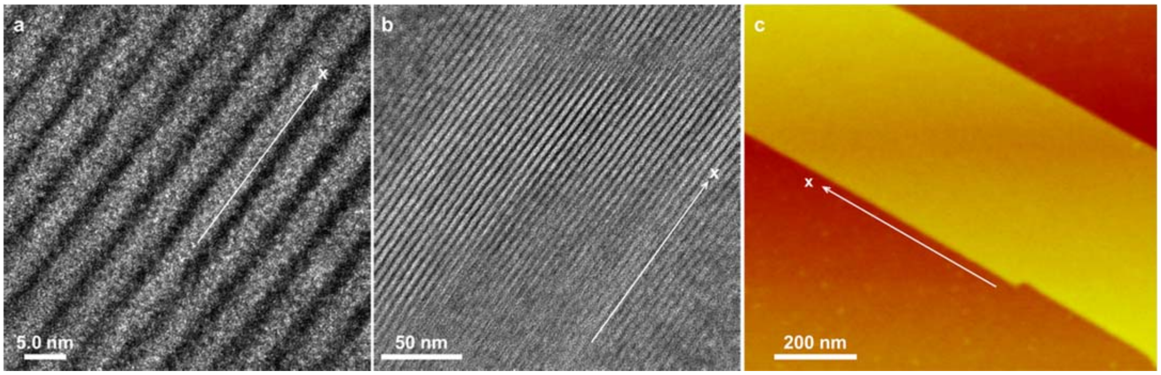


**Supplementary Figure 3 | TEM and AFM characterizations of Pep-5 membranes. a** and **b**, high resolution TEM images to show the well-aligned hydrophilic strips of Pep-**5** membranes along x-direction. **c**, AFM image of one Pep-**5** membrane with height ~ 4.0 nm, the x-direction was labeled for comparison.

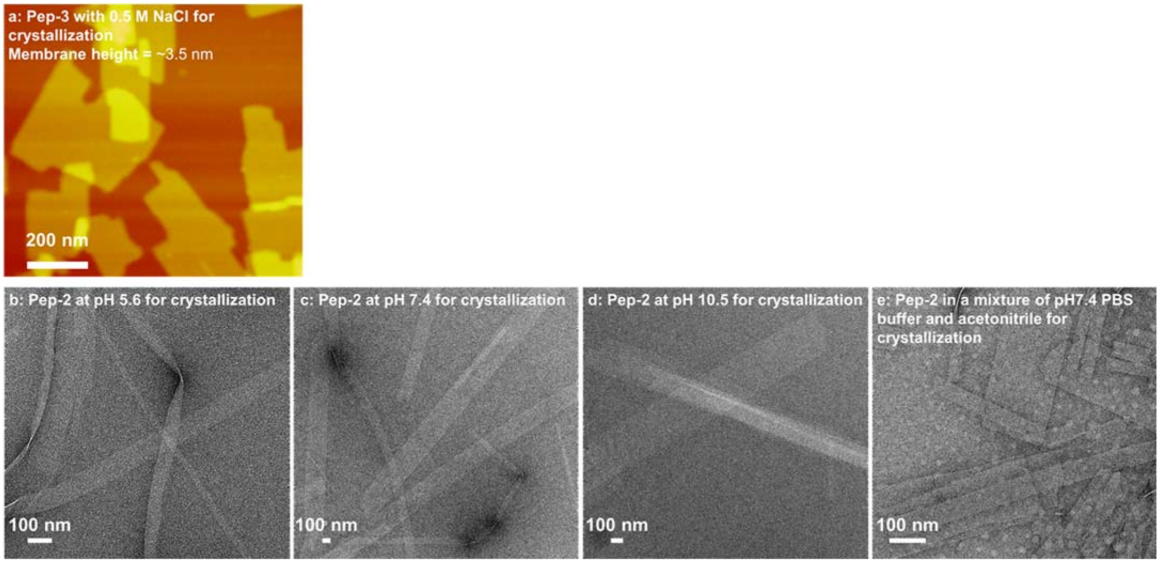


**Supplementary Figure 4 | The importance of inter-peptoid hydrophobic interactions in the assembly of biomimetic membranes. a**, AFM image of biomimetic membranes assembled from Pep-**3** with the presence of over 0.5M NaCl. **b - e**, TEM images of biomimetic membranes assembled from Pep-**2** in the mixture of water and acetonitrile (v/v = 1:1) at pH 5.6 (b), pH 7.4 (c), pH 10.5, and in a mixture of PBS (1X, pH 7.4) buffer and acetonitrile (v/v = 1:1) (d).

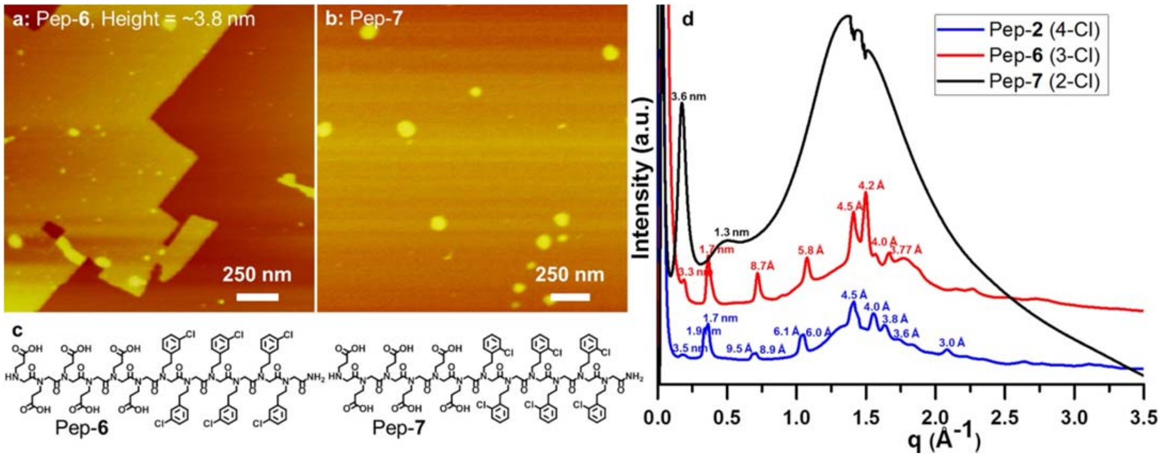


**Supplementary Figure 5 | The influence of -Cl positions in the assembly of biomimetic membranes. a**, An AFM image showing the biomimetic membranes assembled from Pep-**6**. **b**, An AFM image showing the nanoparticles assembled from Pep-**7**. **c**, Structures of Pep-**6** with six N-[2-(3-chlorophenyl)ethyl]glycines ($N_{3\text{-Cl}}$pe) and Pep-**7** with six N-[2-(2-chlorophenyl)ethyl]glycines ($N_{2\text{-Cl}}$pe). **d**, XRD data showing biomimetic membranes assembled from both Pep-**2** (with six $N_{4\text{-Cl}}$pe) and Pep-**6** (with six $N_{3\text{-Cl}}$pe) are highly-crystalline, while nanoparticles assembled from Pep-7 (with six $N_{2\text{-Cl}}$pe) are amorphous; The change of the –Cl locations in hydrophobic side chains caused different packings of hydrophobic domains, the formation of amorphous nanoparticles induced by Pep-**2** is probably due to the weak hydrophobic interactions as a result of large steric hindrance caused by $N_{2\text{-Cl}}$pe side chains.

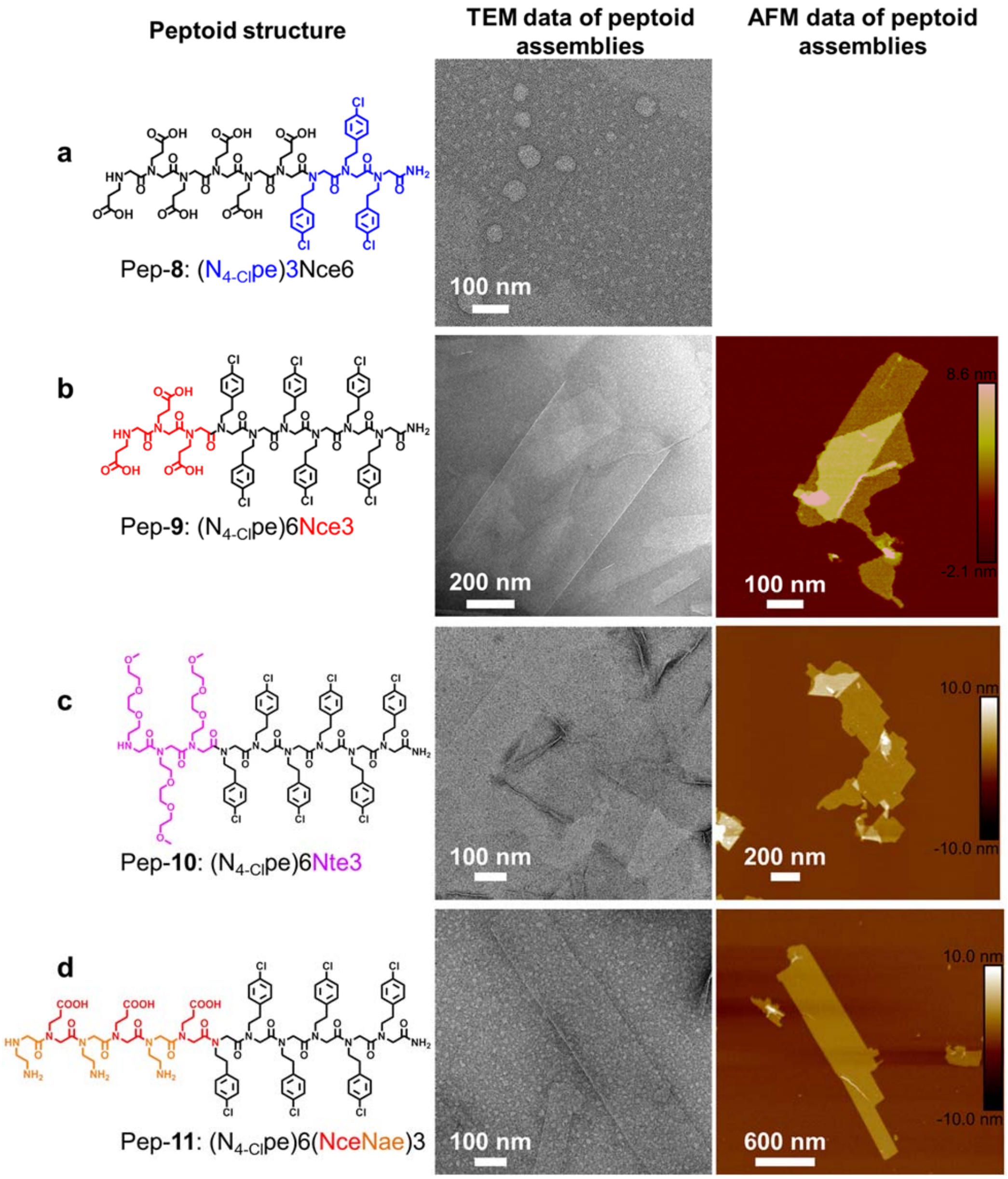


**Supplementary Figure 6 | The ordering of hydrophobic domains is the key to forming membrane structures. a**, Structure of Pep-**8** which has only three hydrophobic $N_{4\text{-Cl}}$pe residues; TEM data show that three $N_{4\text{-Cl}}$pe residues are not enough to stabilize the membrane structure. **b**, Structure of Pep-**9** which has six $N_{4\text{-Cl}}$pe and three hydrophilic Nce residues; TEM and AFM data show that Pep-**9** self-assemble into membranes even with three Nce residues as hydrophilic domain. **c**, Structure of Pep-**10** which has six $N_{4\text{-Cl}}$pe and three hydrophilic Nte residues; TEM and AFM data show that Pep-**10** self-assemble into membranes even with three Nte as the hydrophilic domain; biomimetic membranes assembled from Pep-**10** in the mixture of PBS (1X, pH 7.4) buffer and acetonitrile (v/v = 1:1). **d**, Structure of Pep-**11** which has six $N_{4\text{-Cl}}$pe, three Nce and three Nae residues; TEM and AFM data show that Pep-**10** self-assemble into membranes when hydrophilic domain has half Nce and half Nae residules; biomimetic membranes assembled from Pep-**11** in the mixture of PBS (1X, pH 7.4) buffer and acetonitrile (v/v = 1:1).

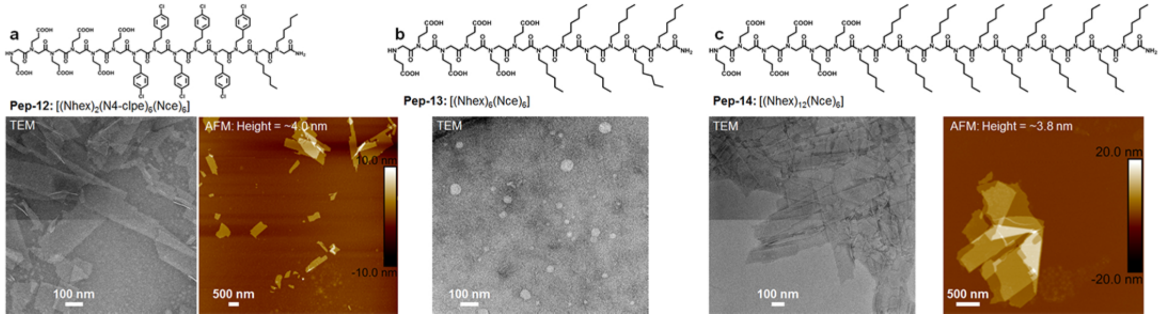


**Supplementary Figure 7 | The significance of hydrophobic interactions in the membrane formation and the similarity of designed peptoids and lipids. a**, Structure of Pep-**12** [$(Nhex)_2(N4\text{-}clpe)_6(Nce)_6$] and the TEM and AFM data of its self-assembled membranes. **b**, Structure of Pep-**13** [$(Nhex)_6(Nce)_6$] and the TEM data of its assemblies. **c**, Structure of Pep-**14** [$(Nhex)_{12}(Nce)_6$] and the TEM and AFM data of its self-assembled membranes.

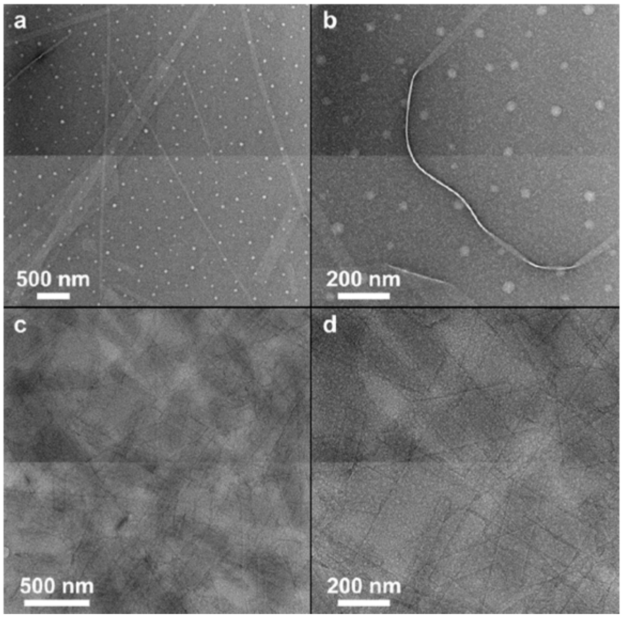


**Supplementary Figure 8 | Peptoid membrane formation is through an anisotropic crystallization process. a** and **b,** Pep-**3** nanoparticles and nanoribbons formed about one day later. **c** and **d,** Pep-**3** membranes formed about two days later**.** 2% phosphotungstic acid was used for negative staining.

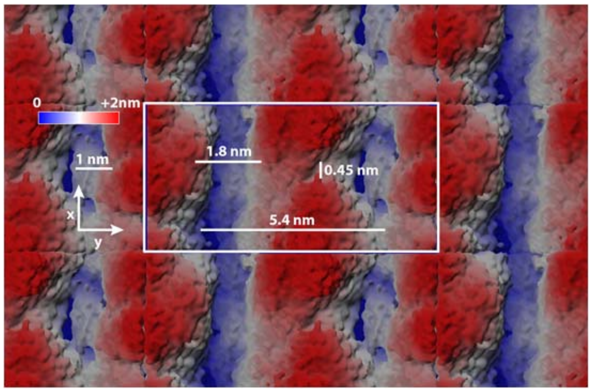


**Supplementary Figure 9 | Molecular dynamics of a structural model for peptoid nanomembranes.** Top view of the solvent accessible surface area for the first solvation shell water molecules above the membrane averaged over the last 200 ns of the trajectory for the membrane structure formed in N=96 simulations of the lipid-like Pep-1 (carboxyl side-chains were in the protonated state, and hydrophilic domains formed strips along x-direction). The height difference is color coded from blue (0nm) to red (2nm) setting the zero to the first solvation shell around the hydrophobic core of the membrane. The white rectangle represents the super cell of the simulation.

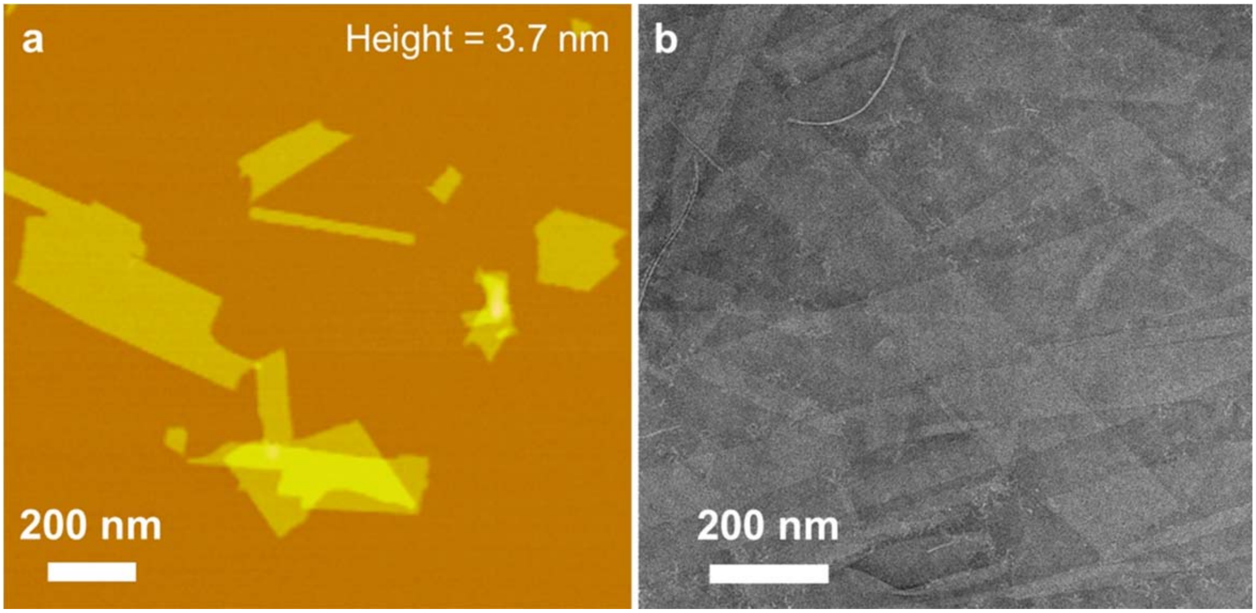


**Supplementary Figure 10 | Peptoid membranes assembled from 1-Ntyr-Pep-2.** AFM (a) and TEM (b) characterizations; 2% phosphotungstic acid was used for negative staining.

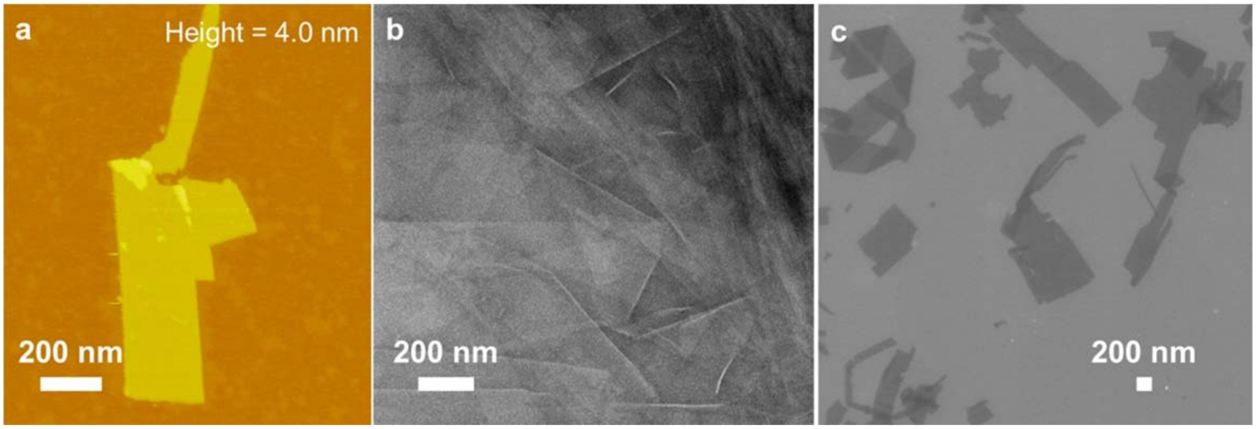


**Supplementary Figure 11 | Peptoid membranes assembled from 1-Npyr-Pep-2.** AFM (a), TEM (b), and SEM (c) characterizations.

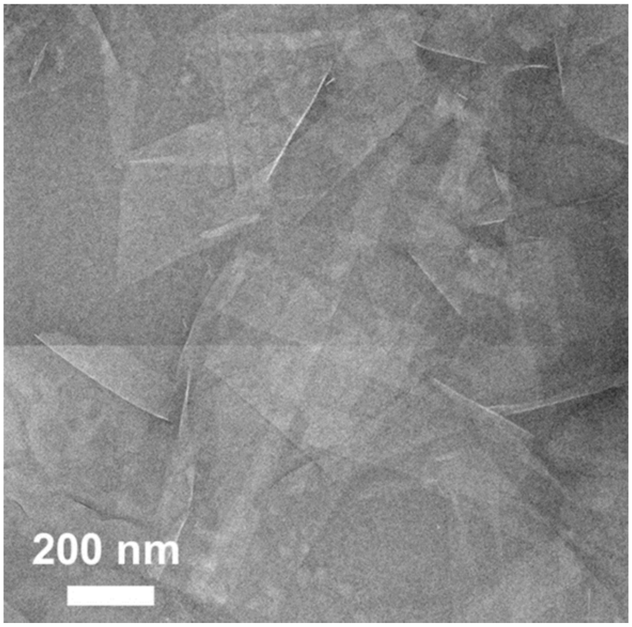


**Supplementary Figure 12 | Peptoid membranes assembled from 1-Nhex-Pep-2.** TEM characterization of peptoid membranes; 2% phosphotungstic acid was used for negative staining.

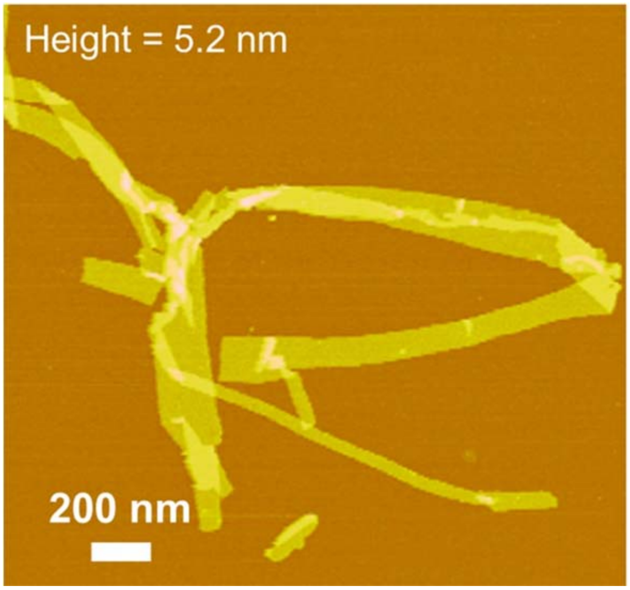


**Supplementary Figure 13 | Peptoid membranes assembled from 1,8-(Nazo)$_2$-Pep-2.** AFM characterization of peptoid membranes.

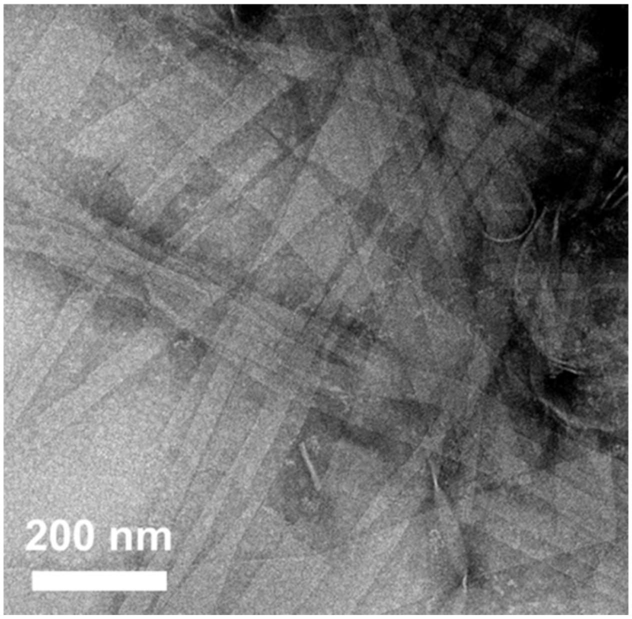


**Supplementary Figure 14 | Peptoid membranes assembled from 1,8-(Ntrp)$_2$-Pep-2.** TEM characterization of peptoid membranes.

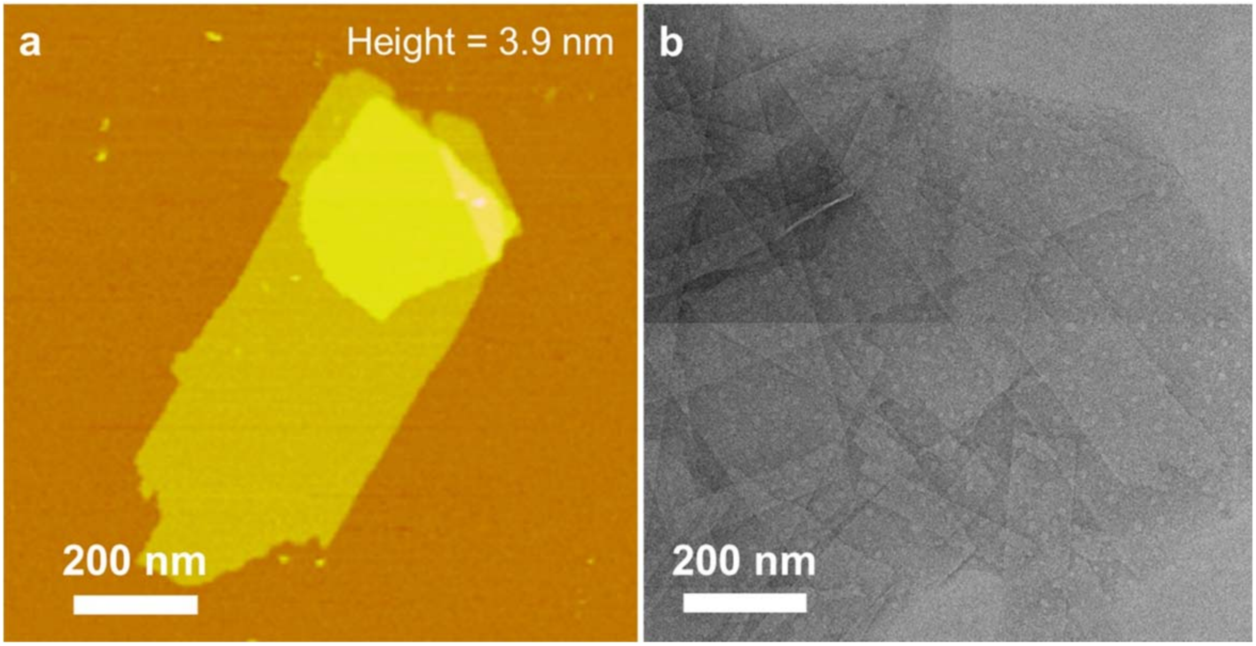


**Supplementary Figure 15 | Peptoid membranes assembled from 1,8-(Nse)$_2$-Pep-2.** AFM (a) and TEM (b) characterizations; 2% phosphotungstic acid was used for negative staining.

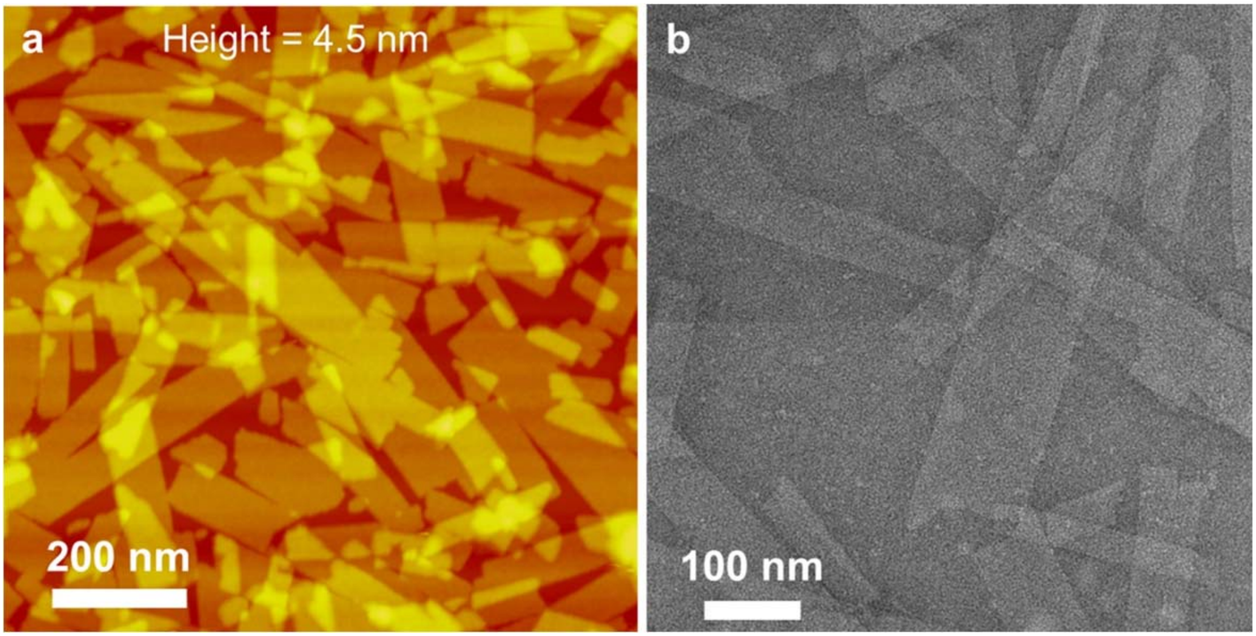


**Supplementary Figure 16 | Peptoid membranes assembled from 1,8-(Ntyr)$_2$-Pep-2.** AFM (a) and TEM (b) characterizations; 2% phosphotungstic acid was used for negative staining.

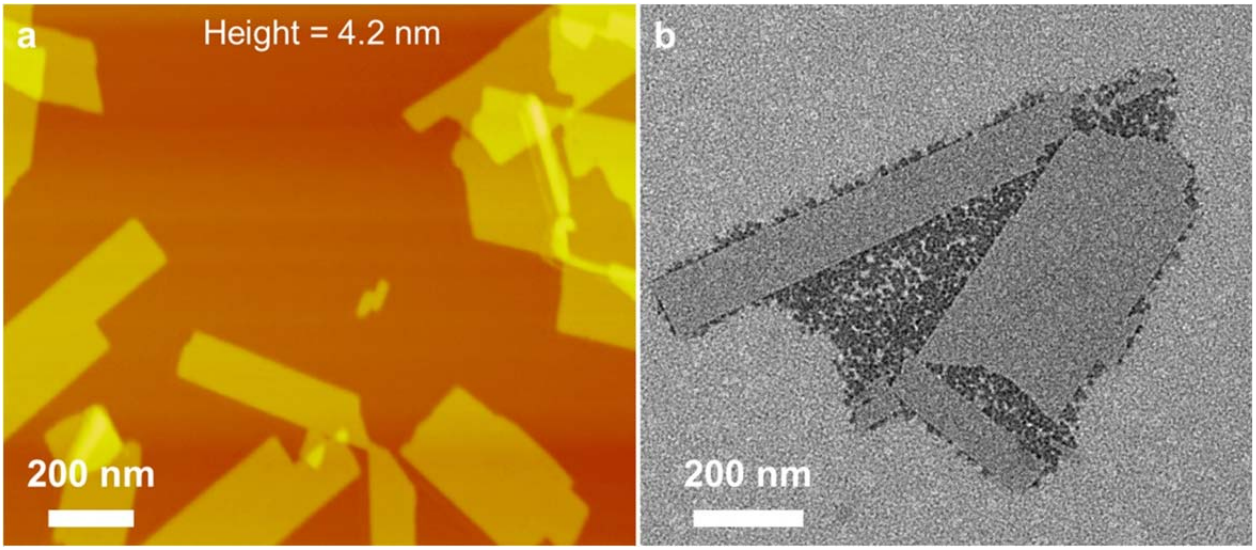


**Supplementary Figure 17 | Peptoid membranes assembled from 1-Nse-8-Ntyr-Pep-2.** AFM (a) and TEM (b) characterizations; 2% phosphotungstic acid was used for negative staining.

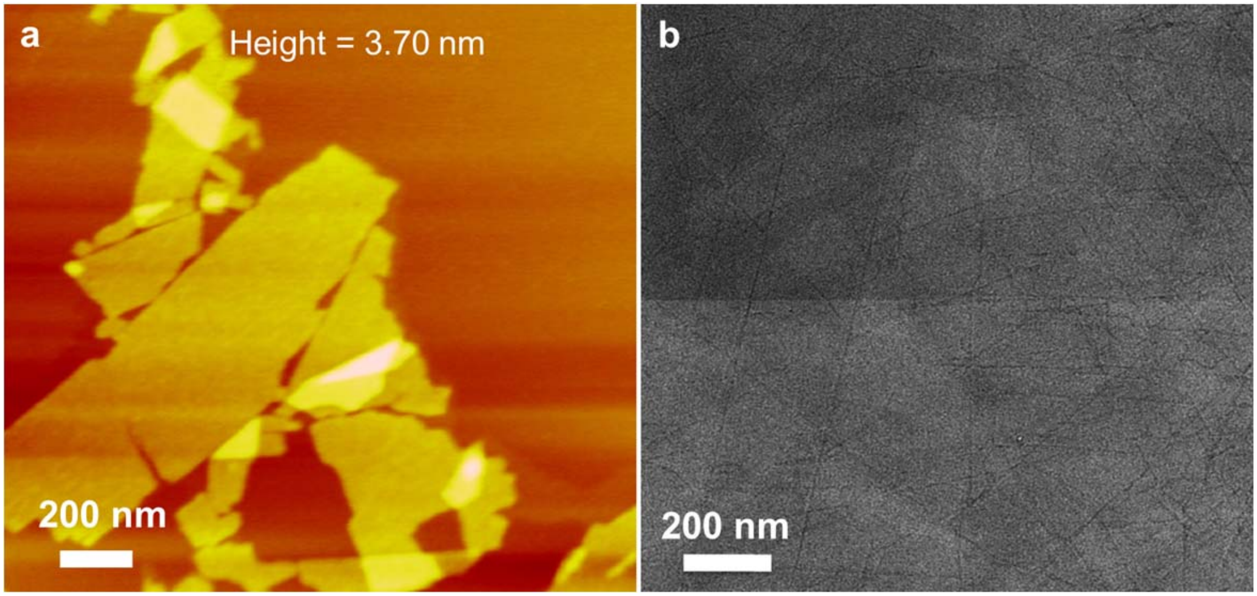


**Supplementary Figure 18 | Peptoid membranes assembled from 13-Nte-Pep-2.** AFM (a) and TEM (b) characterizations; 2% phosphotungstic acid was used for negative staining.

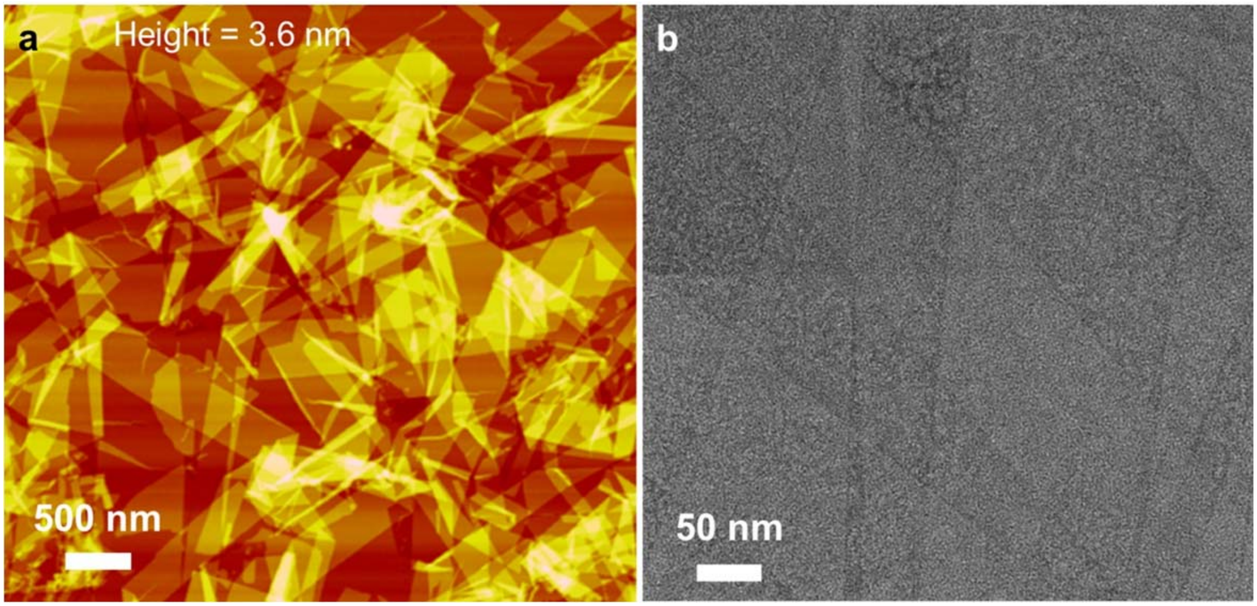


**Supplementary Figure 19 | Peptoid membranes assembled from 13-Nhis-Pep-2.** AFM (a) and TEM (b) characterizations; 2% phosphotungstic acid was used for negative staining.

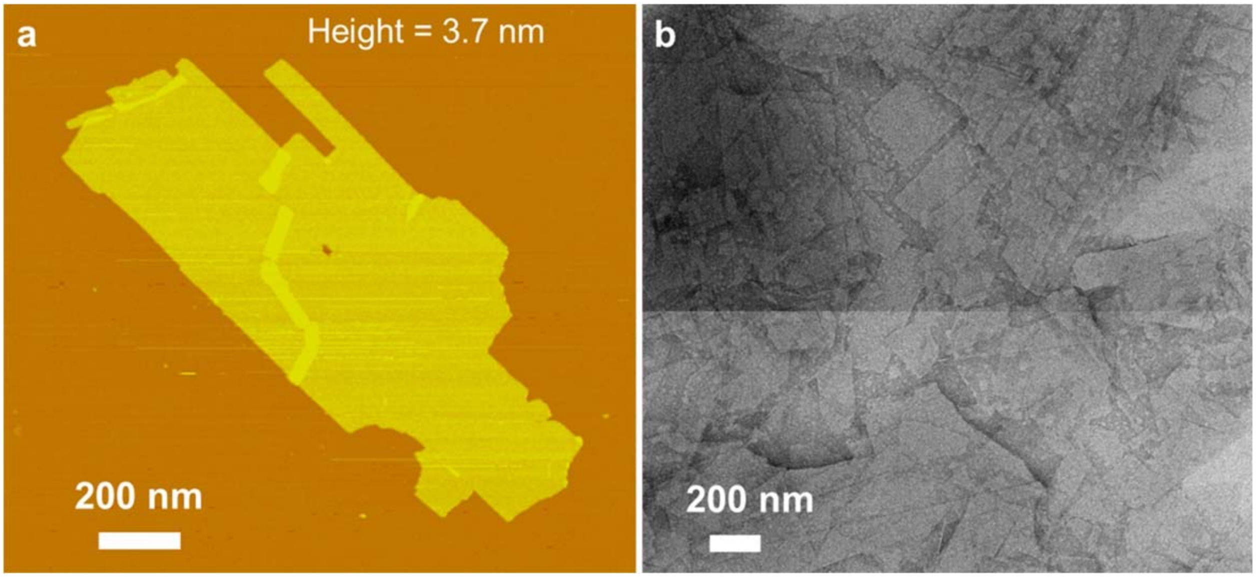


**Supplementary Figure 20 | Peptoid membranes assembled from 13-Ntyr-Pep-2.** AFM (a) and TEM (b) characterizations; 2% phosphotungstic acid was used for negative staining.

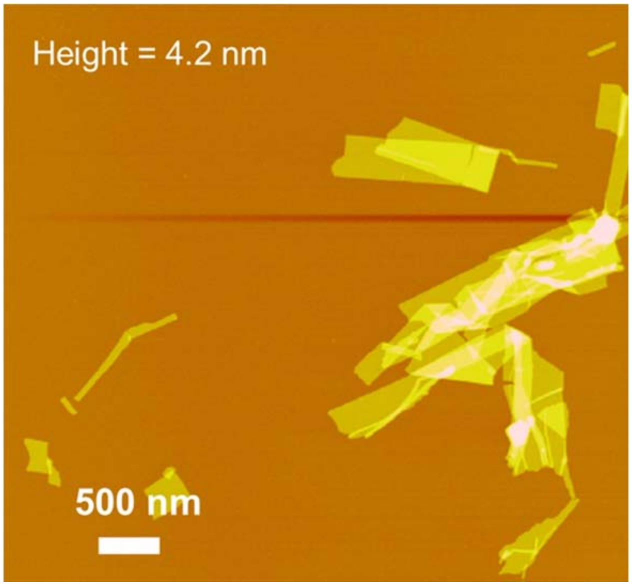


**Supplementary Figure 21 | Peptoid membranes assembled from 13-Nbce-Pep-2.** AFM characterization of peptoid membranes.

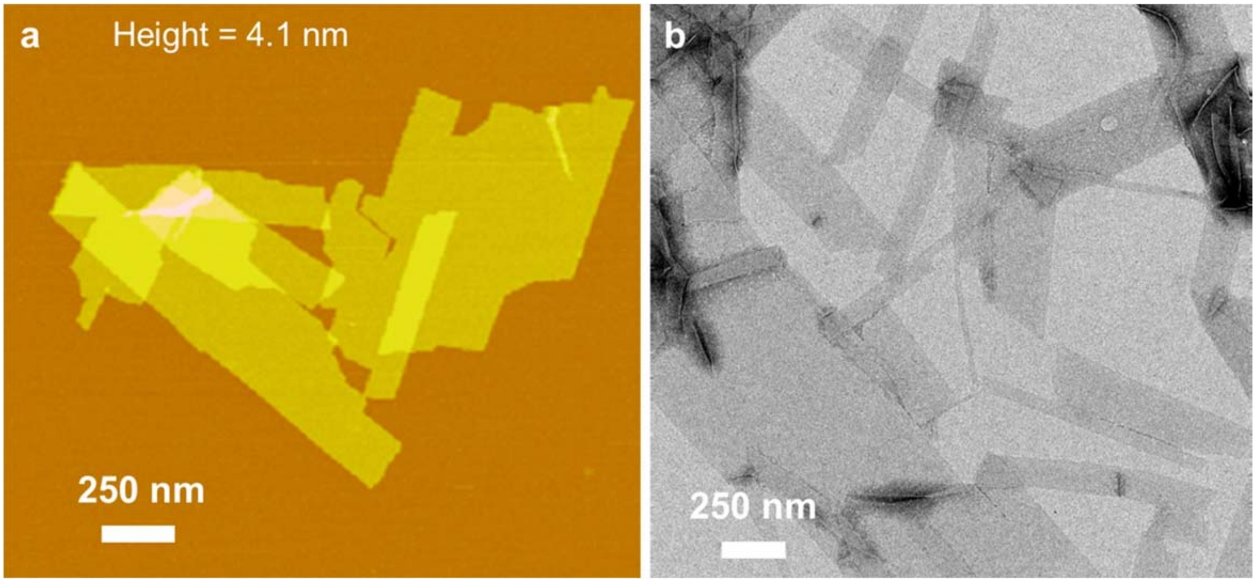


**Supplementary Figure 22 | Peptoid membranes assembled from 1,14-(Nse)$_2$-Pep-2.** AFM (a) and TEM (b) characterizations; 2% phosphotungstic acid was used for negative staining.

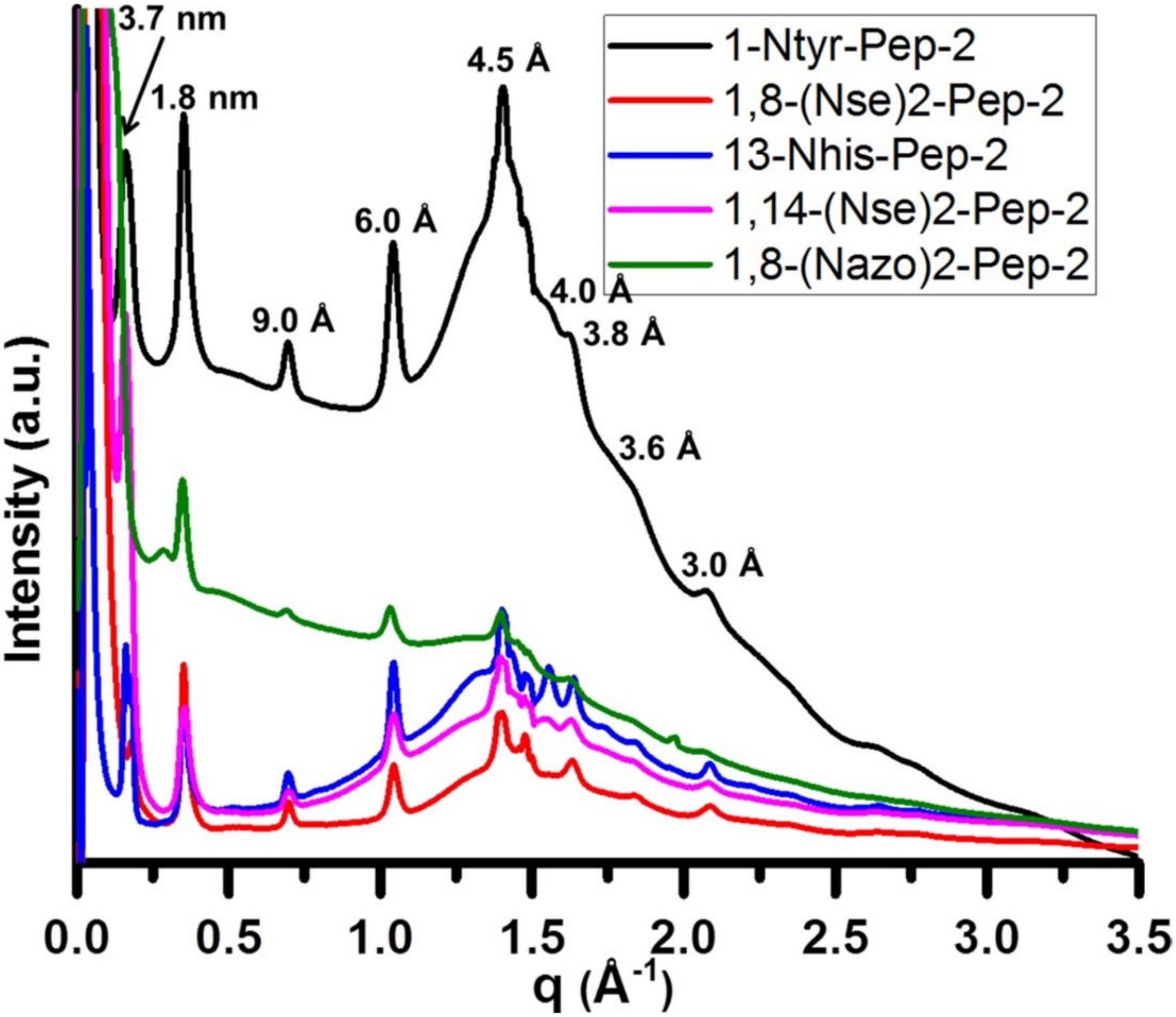


**Supplementary Figure 23 | Peptoids with functional objects assembled into biomimetic membranes with similar structures.** XRD data of membranes assembled from 1-Ntyr-**Pep-2**, 1,8-$(Nse)_2$-**Pep-2**, 13-Nhis-**Pep-2**, 1,14-$(Nse)_2$-**Pep-2**, and 1,8-$(Nazo)_2$-**Pep-2**; they all exhibit similar XRD patterns to those of Pep-**2**, showing structural similarity.

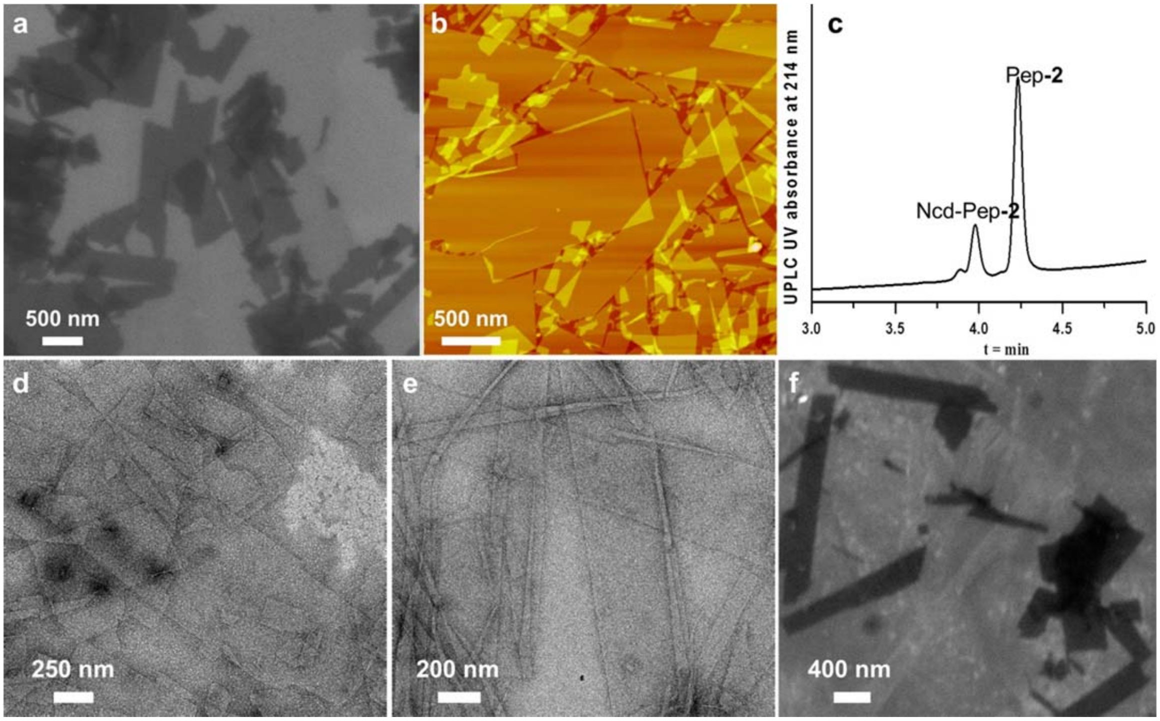


**Supplementary Figure 24 | Incorporating functional objects through co-crystallization. a**, A SEM image showing 2D membranes co-assembled from **Pep-2** and Ncd-**Pep-2** with molar ratio 4:1. **b**, An AFM image showing the co-assembled membranes. **c**, UPLC data showing the co-assembled membranes from **Ncd-Pep-2** & **Pep-2** have the molar ratio of Ncd-Pep-**2** *vs* Pep-**2** is almost 1 : 4 (integration of UPLC peaks). **d**, A TEM image showing 2D membranes co-assembled from **Pep-2** and Ncd-**Pep-2** with molar ratio 9:1. **e**, A TEM image showing 2D membranes co-assembled from **Pep-2** and Ncd-**Pep-2** with molar ratio 1:1. **f**, A SEM image showing 2D membranes co-assembled from **Pep-2** and Ncd-**Pep-2** with molar ratio 1:1.

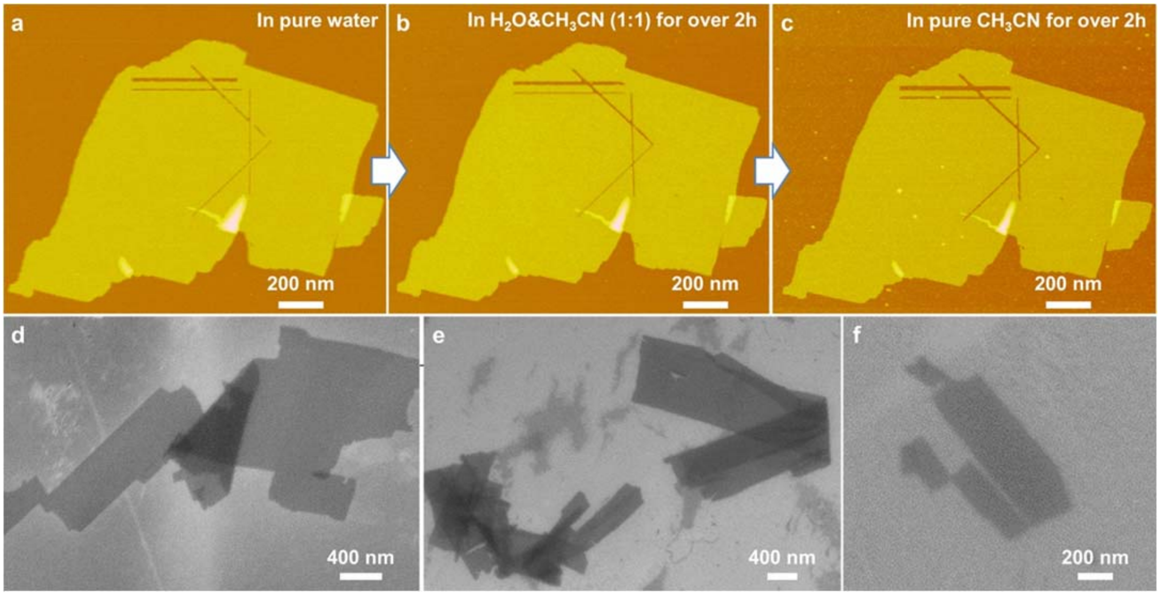


**Supplementary Figure 25 | Peptoid membranes are highly stable. a**, An *in situ* AFM image of one Pep-3 membrane in pure water, some defects were purposely introduced using mechanical forces for membrane self-repair experiments. **b**, An *in situ* AFM images show that no significant disruptions were observed when peptoid membranes were incubated in $H_2O$&$CH_3CN$ (1:1) solution for over 2h. **c**, An *in situ* AFM image show the peptoid membrane is stable even in pure $CH_3CN$ for over 2h. **d,** A SEM image of **Pep-2** membranes which were incubated in pure $CH_3CN$ for over 6h. **e**, A SEM image showing peptoid membranes co-assembled from **Pep-2** and Ncd-**Pep-2** with molar ratio 1:1, these membranes were incubated in pure $CH_3CN$ for over 6h before SEM experiment. **f**, A SEM image showing the 1-Npyr-**Pep-2** membranes after they were incubated in pure $CH_3CN$ for over 6h.

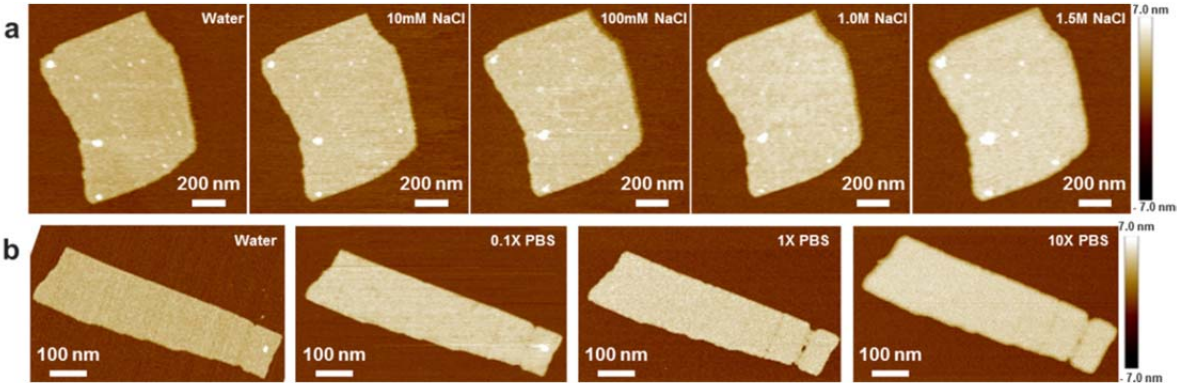


**Supplementary Figure 26 | Salt-induced thickness changes of peptoid membranes. a,** *In situ* AFM image of one Pep-**3** membrane in pure water, 10 mM NaCl, 100 mM NaCl, 1.0M NaCl and 1.5 M NaCl showing membrane thickness change. **b,** *In situ* AFM image of one Pep-**3** membrane in pure water, 0.1x PBS, 1x PBS and 10x PBS buffer showing membrane thickness change.

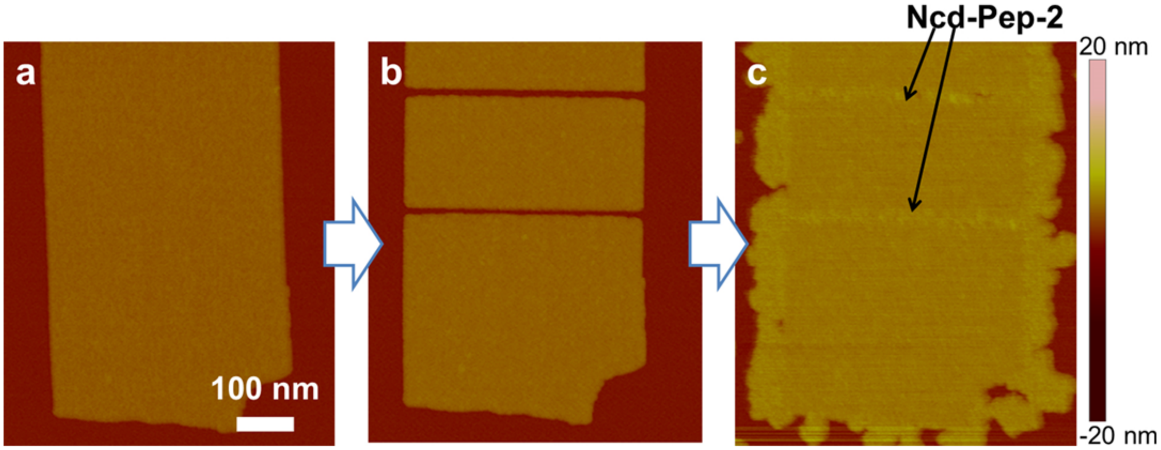


**Supplementary Figure 27 | The nanoscale patterning of Ncd-Pep-2 within Pep-2 membranes. a,** An *In situ* AFM image showing one Pep-**2** membrane in water. **b**, An *In situ* AFM image showing one Pep-**2** membrane with mechanically-induced defects. **c**, An *In situ* AFM image showing the nanoscale patterning of Ncd-Pep-**2** within Pep-**2** membrane.

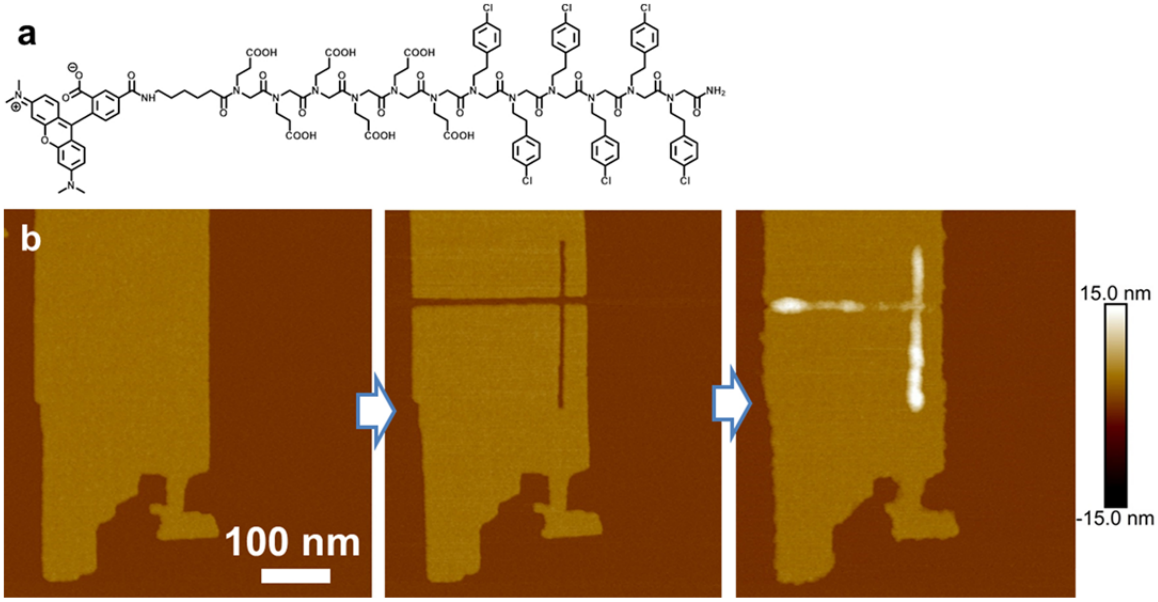


**Supplementary Figure 28 | The nanoscale patterning of NHS-Rhodamine-labeled Pep-3 within Pep-3 membranes. a,** Structure of NHS-Rhodamine-labeled Pep-3. **b,** The nanoscale patterning of NHS-Rhodamine-Pep-3 within Pep-3 membranes; (left) An *In situ* AFM image showing one Pep-2 membrane in water; (middle) An *In situ* AFM image showing one Pep-3 membrane with mechanically-induced defects; (right) An *In situ* AFM image showing the nanoscale patterning of NHS-Rhodamine-Pep-3 within Pep-3 membrane.

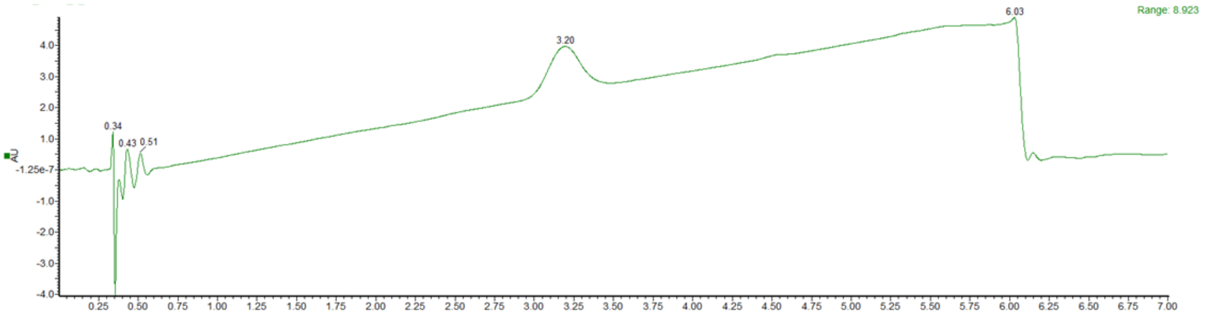


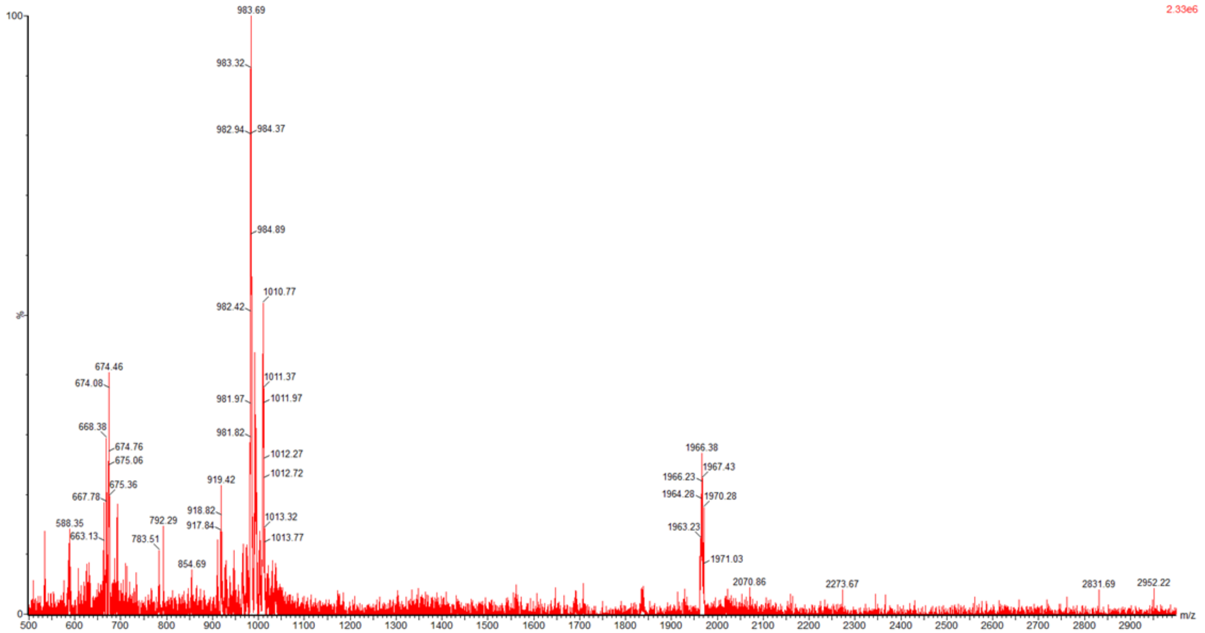


**Supplementary Figure 29 | UPLC-MS characterization of Pep-1.** (Top image) UPLC characterization of Pep-**1** with the gradient of 40 - 80% $CH_3CN$ in $H_2O$. (Bottom image) MS characterization of Pep-**1.**

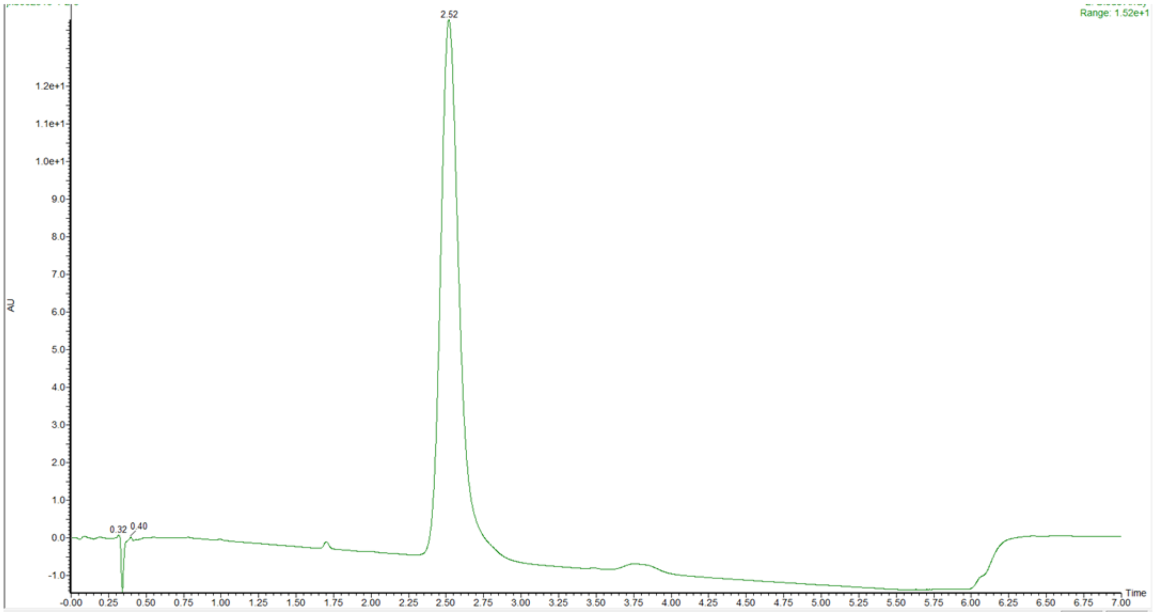


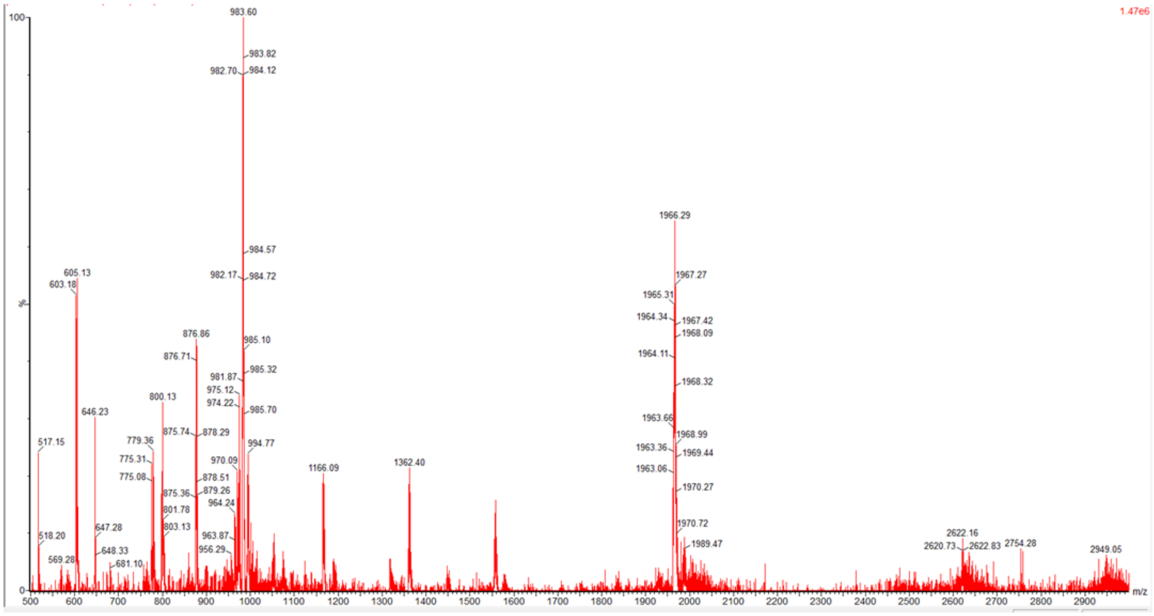


**Supplementary Figure 30 | UPLC-MS characterization of Pep-2.** (Top image) UPLC characterization of Pep-**2** with the gradient of 55 - 75% $CH_3CN$ in $H_2O$. (Bottom image) MS characterization of Pep-**2.**

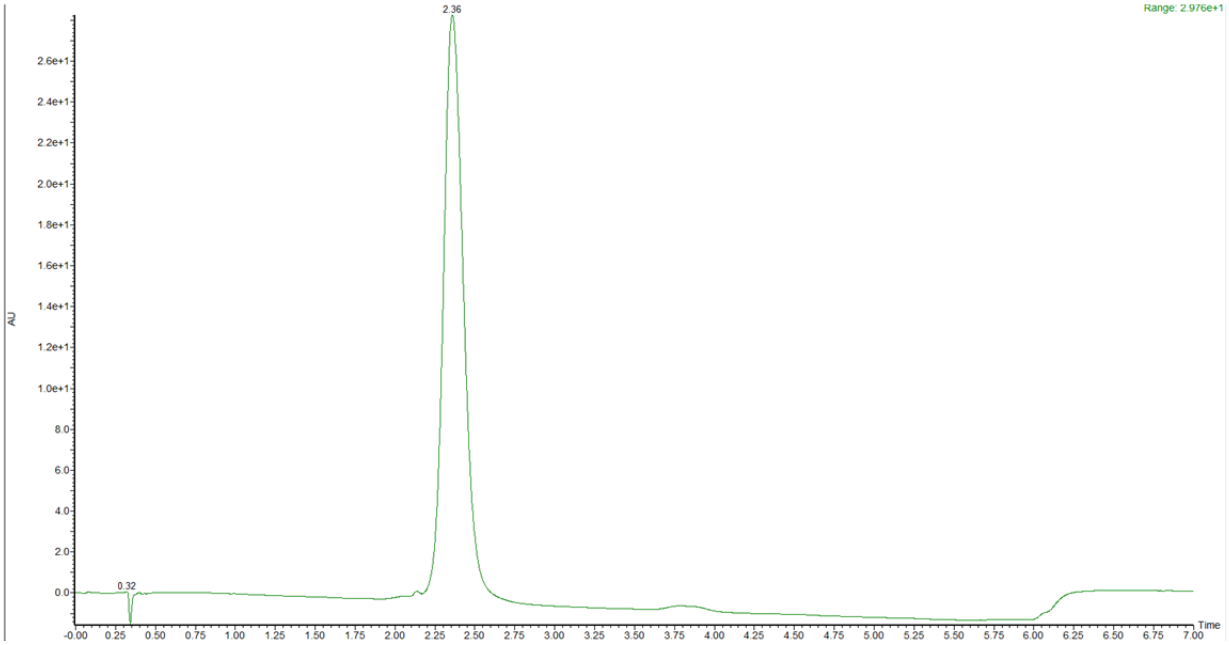


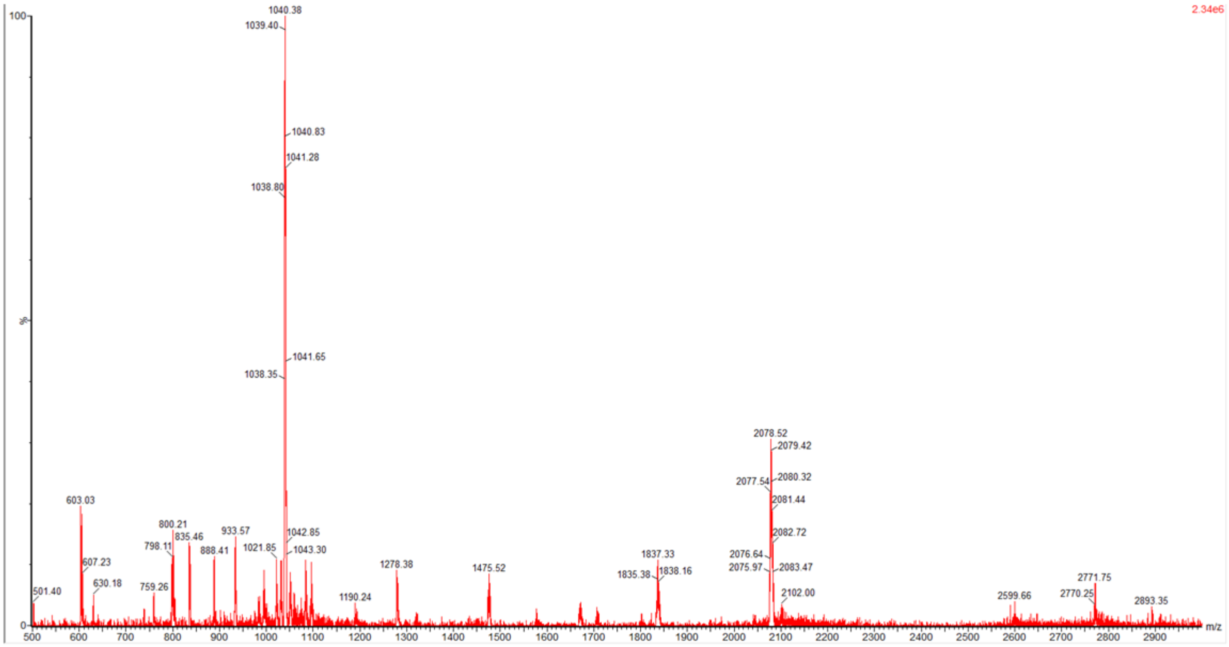


**Supplementary Figure 31 | UPLC-MS characterization of Pep-3.** (Top image) UPLC characterization of Pep-**3** with the gradient of 55 - 75% $CH_3CN$ in $H_2O$. (Bottom image) MS characterization of Pep-**3.**

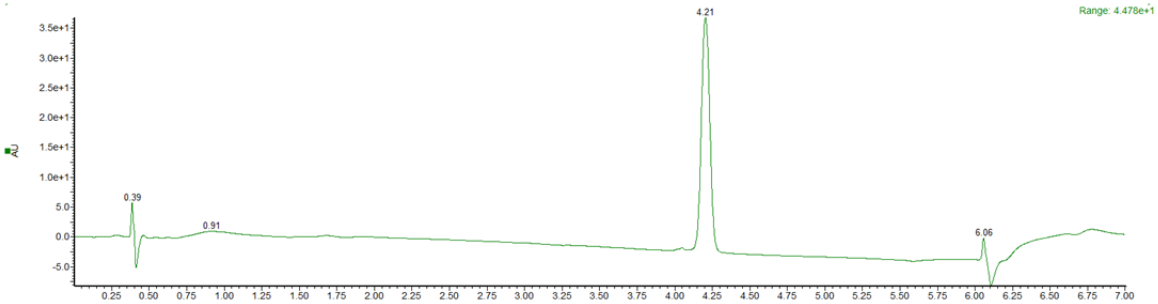


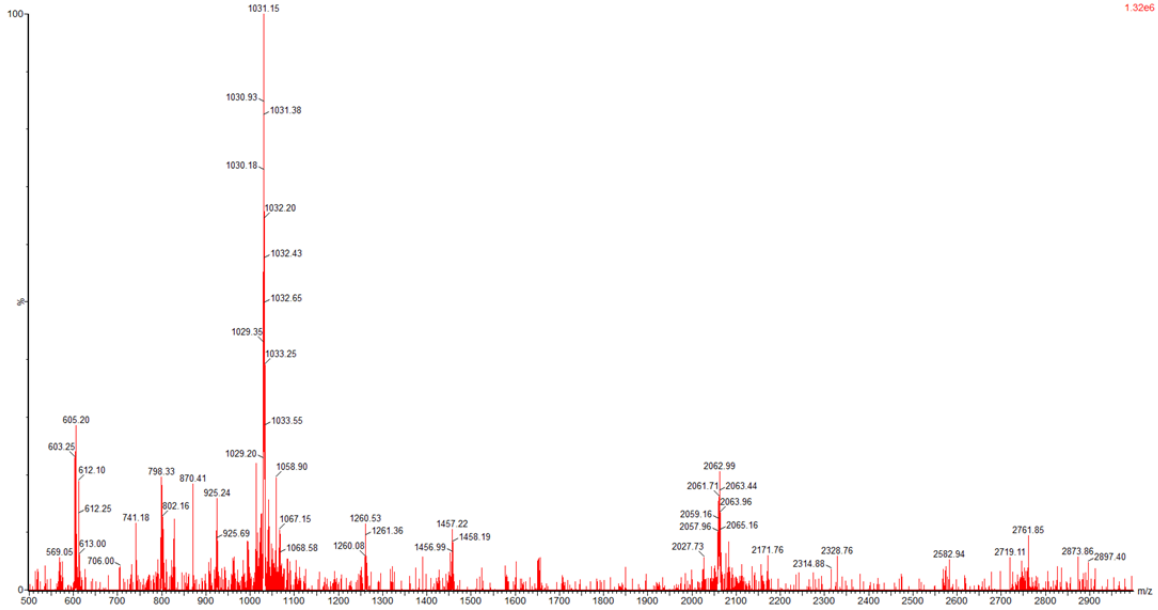


**Supplementary Figure 32 | UPLC-MS characterization of Pep-4.** (Top image) UPLC characterization of Pep-**4** with the gradient of 5 - 95% $CH_3CN$ in $H_2O$. (Bottom image) MS characterization of Pep-**4.**

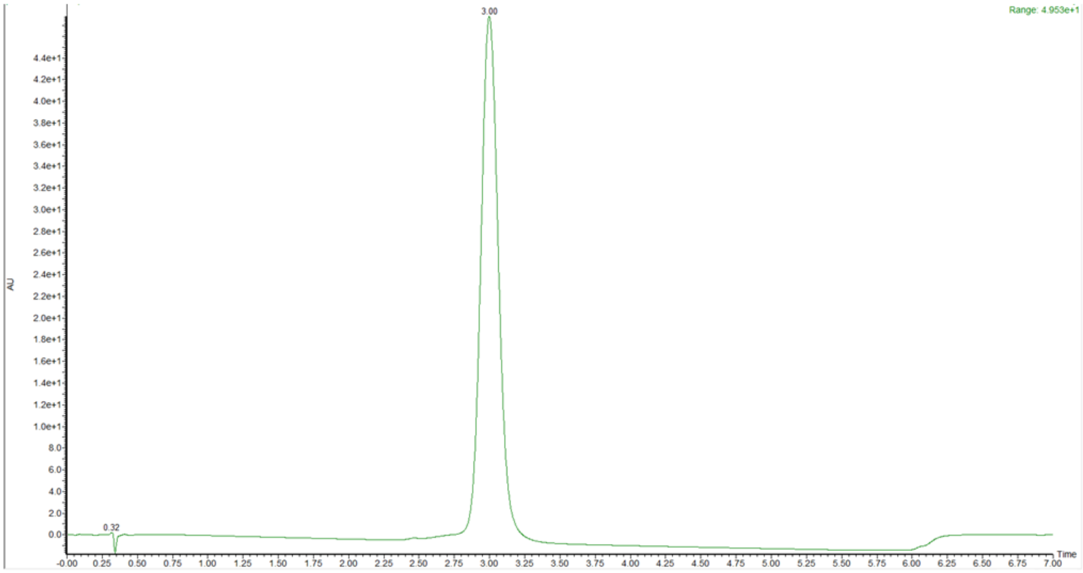


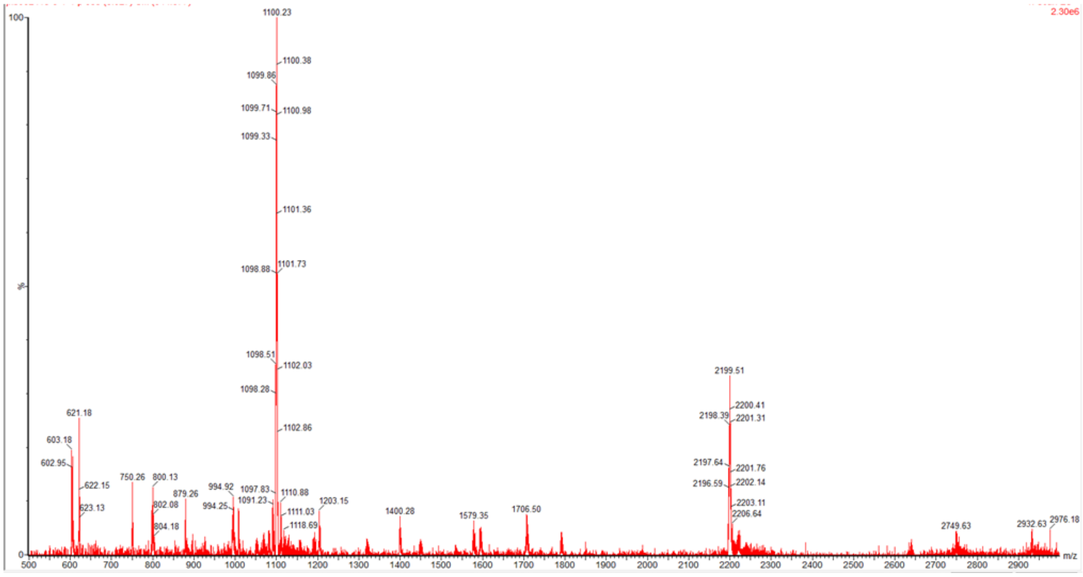


**Supplementary Figure 33 | UPLC-MS characterization of Pep-5.** (Top image) UPLC characterization of Pep-**5** with the gradient of 40 - 80% $CH_3CN$ in $H_2O$. (Bottom image) MS characterization of Pep-**5.**

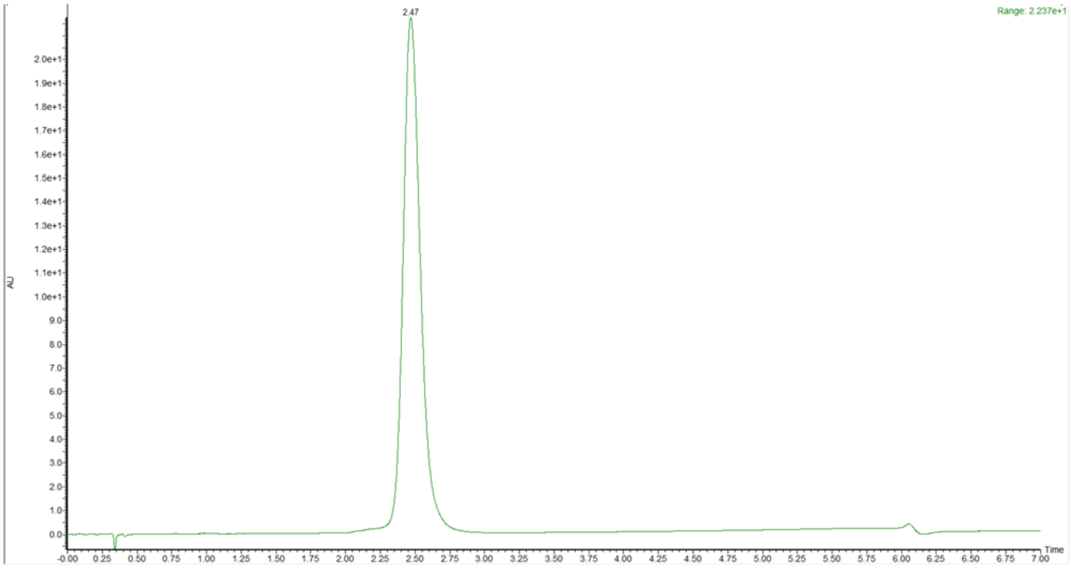


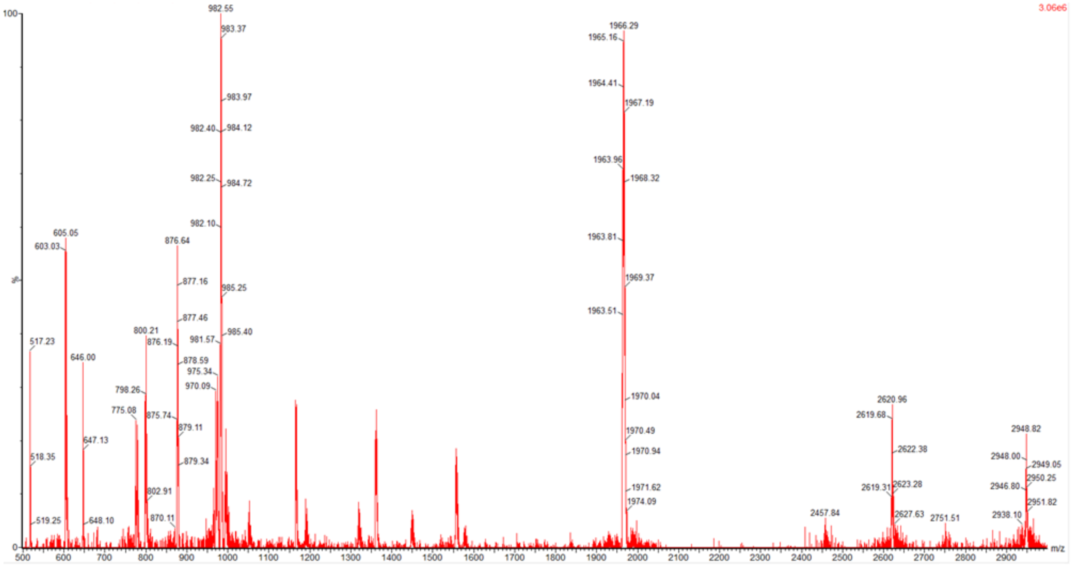


**Supplementary Figure 34 | UPLC-MS characterization of Pep-6.** (Top image) UPLC characterization of Pep-**6** with the gradient of 55 - 75% $CH_3CN$ in $H_2O$. (Bottom image) MS characterization of Pep-**6.**

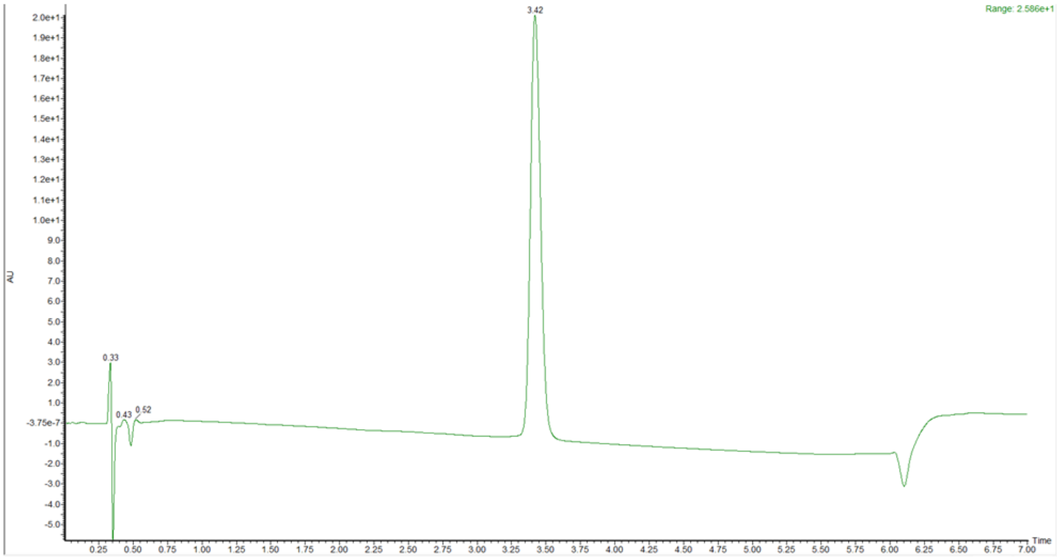


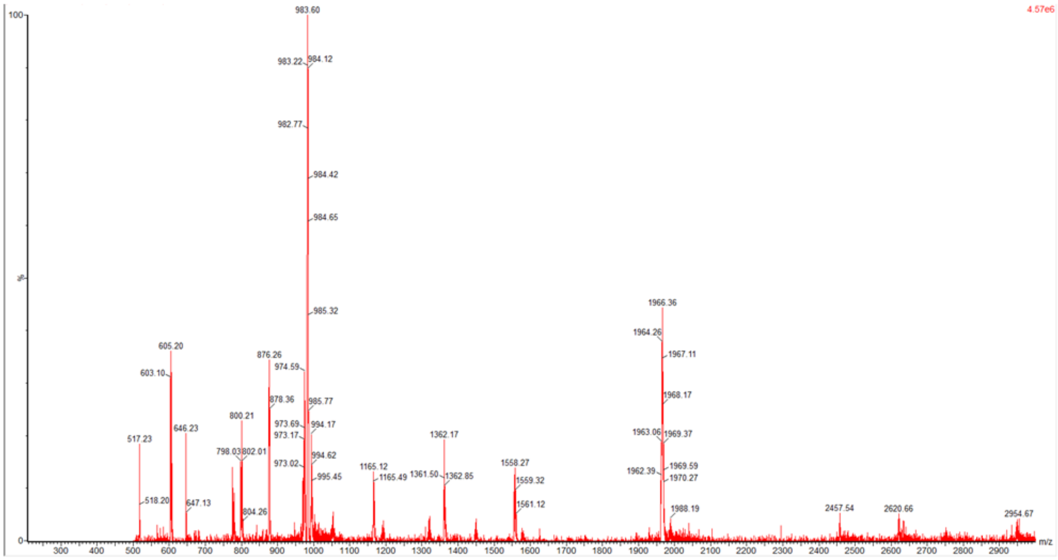


**Supplementary Figure 35 | UPLC-MS characterization of Pep-7.** (Top image) UPLC characterization of Pep-**7** with the gradient of 40 - 80% $CH_3CN$ in $H_2O$. (Bottom image) MS characterization of Pep-**7.**

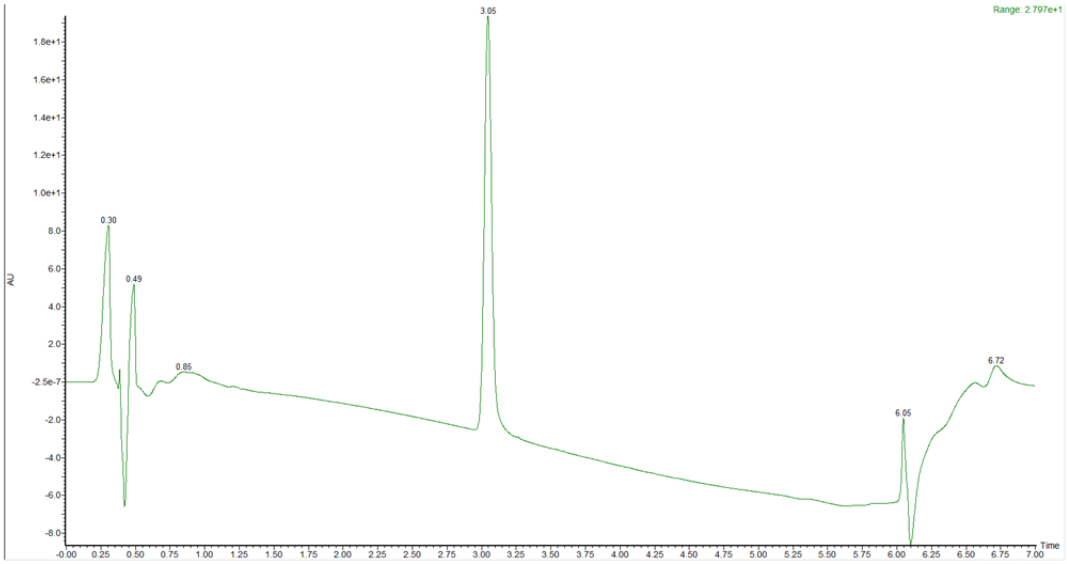


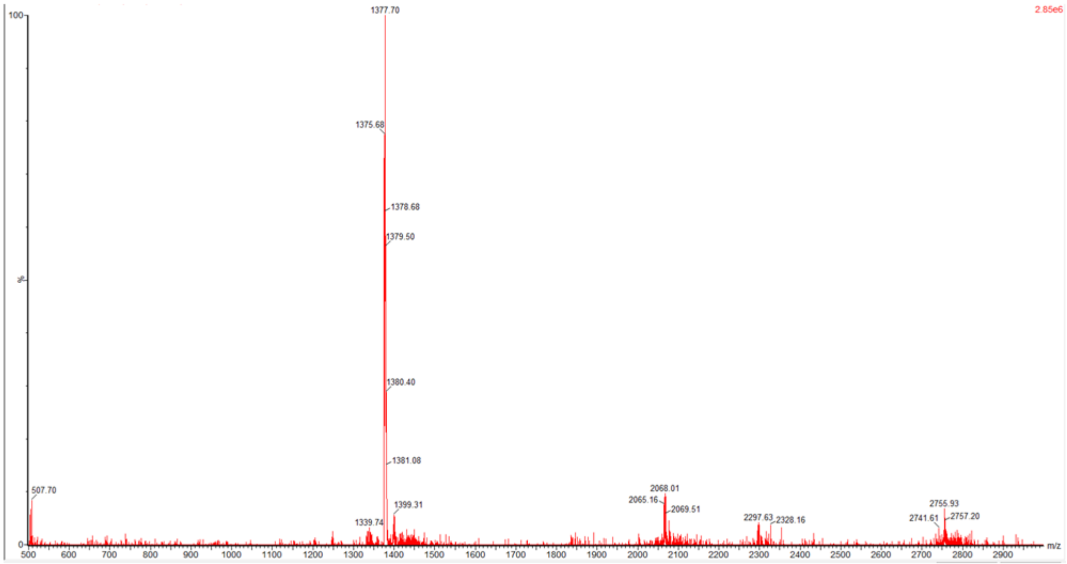


**Supplementary Figure 36 | UPLC-MS characterization of Pep-8.** (Top image) UPLC characterization of Pep-**8** with the gradient of 5 - 95% $CH_3CN$ in $H_2O$. (Bottom image) MS characterization of Pep-**8.**

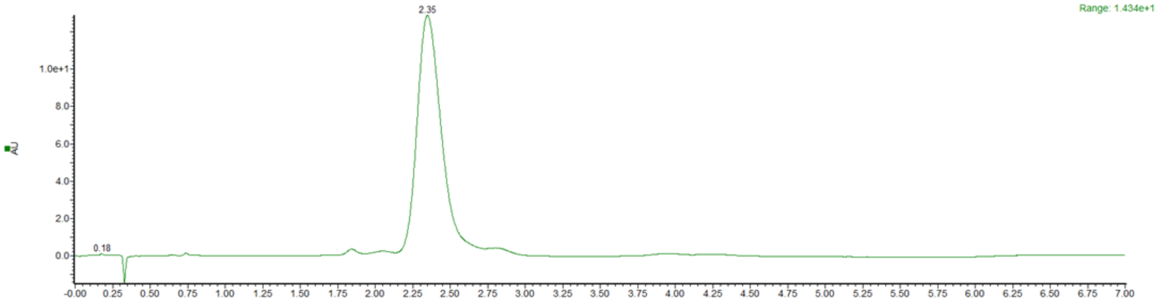


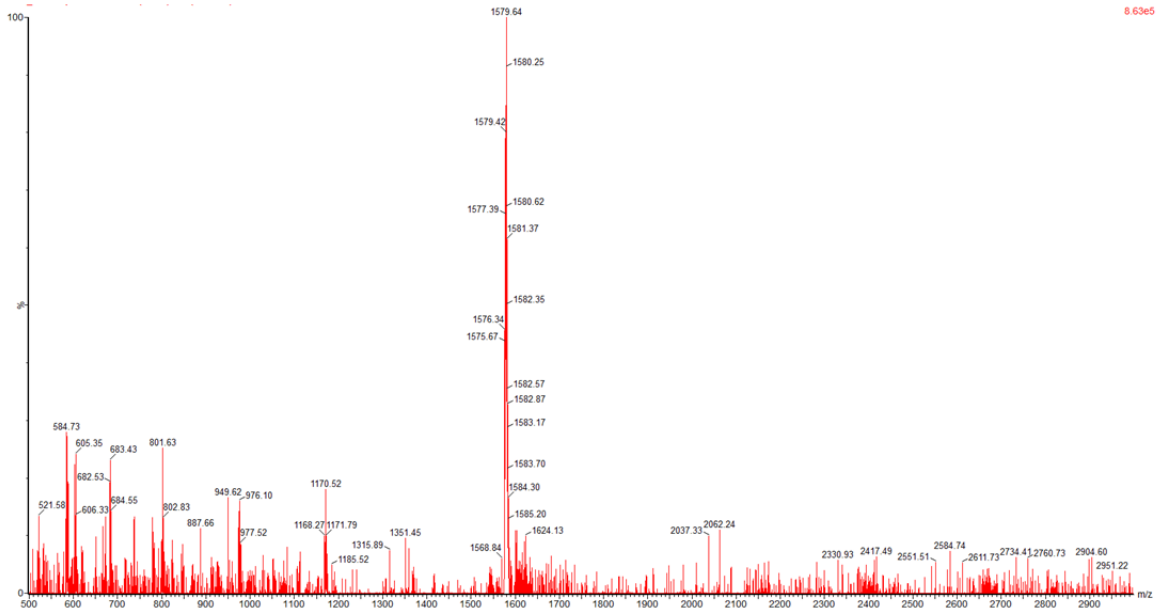


**Supplementary Figure 37 | UPLC-MS characterization of Pep-9.** (Top image) UPLC characterization of Pep-**9** with the gradient of 60 - 65% $CH_3CN$ in $H_2O$. (Bottom image) MS characterization of Pep-**9.**

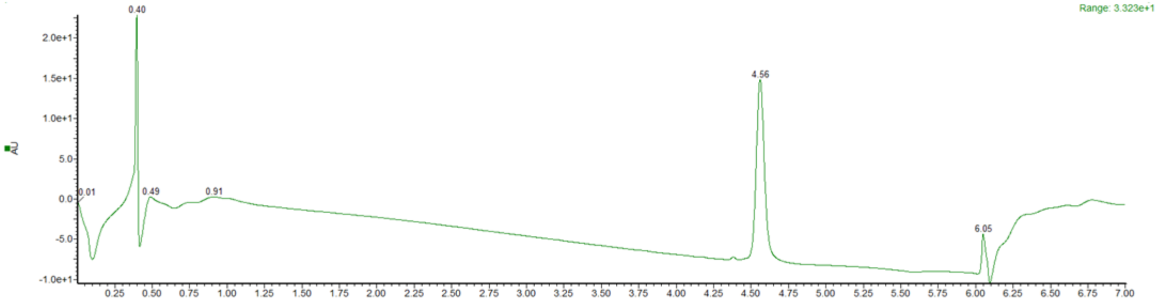


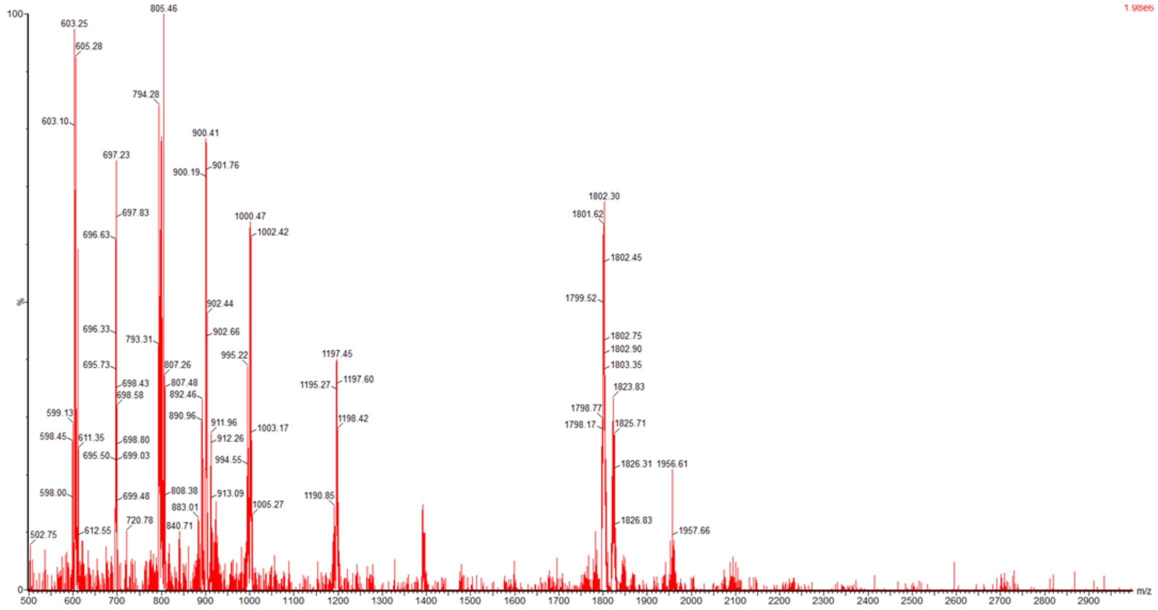


**Supplementary Figure 38 | UPLC-MS characterization of Pep-10.** (Top image) UPLC characterization of Pep-**10** with the gradient of 5 - 95% $CH_3CN$ in $H_2O$. (Bottom image) MS characterization of Pep-**10.**

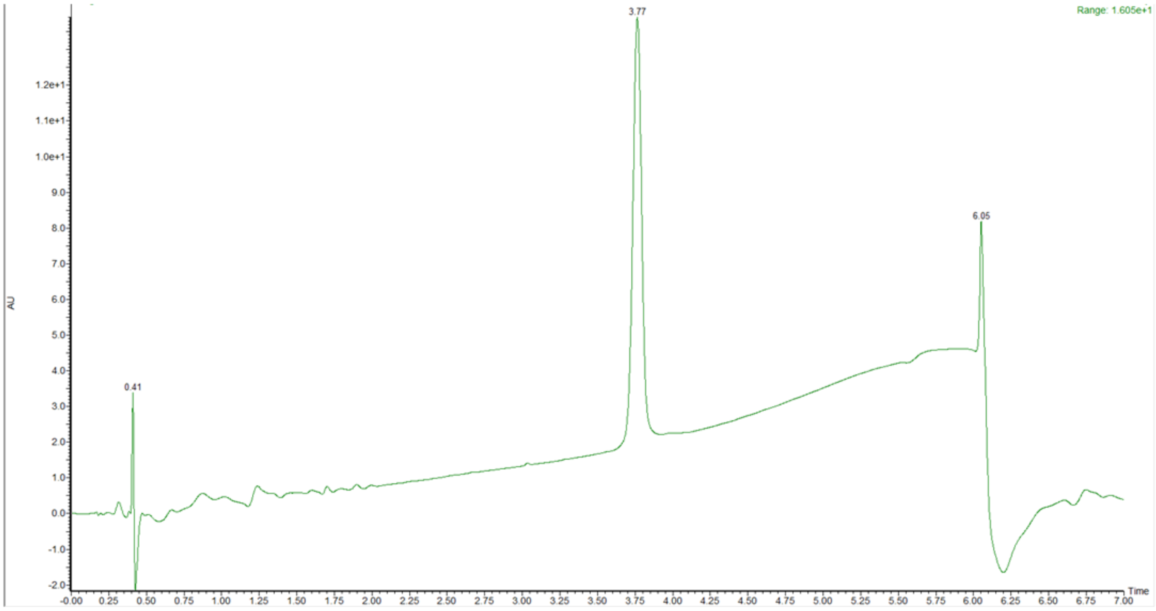


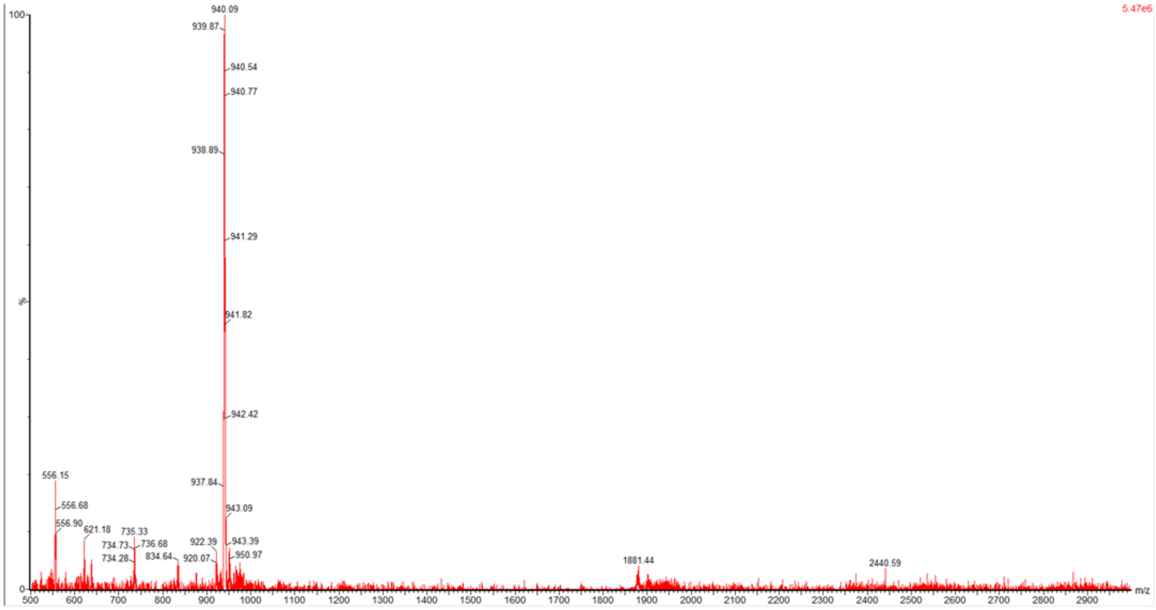


**Supplementary Figure 39 | UPLC-MS characterization of Pep-11.** (Top image) UPLC characterization of Pep-**11** with the gradient of 45 - 65% $CH_3CN$ in $H_2O$. (Bottom image) MS characterization of Pep-**11.**

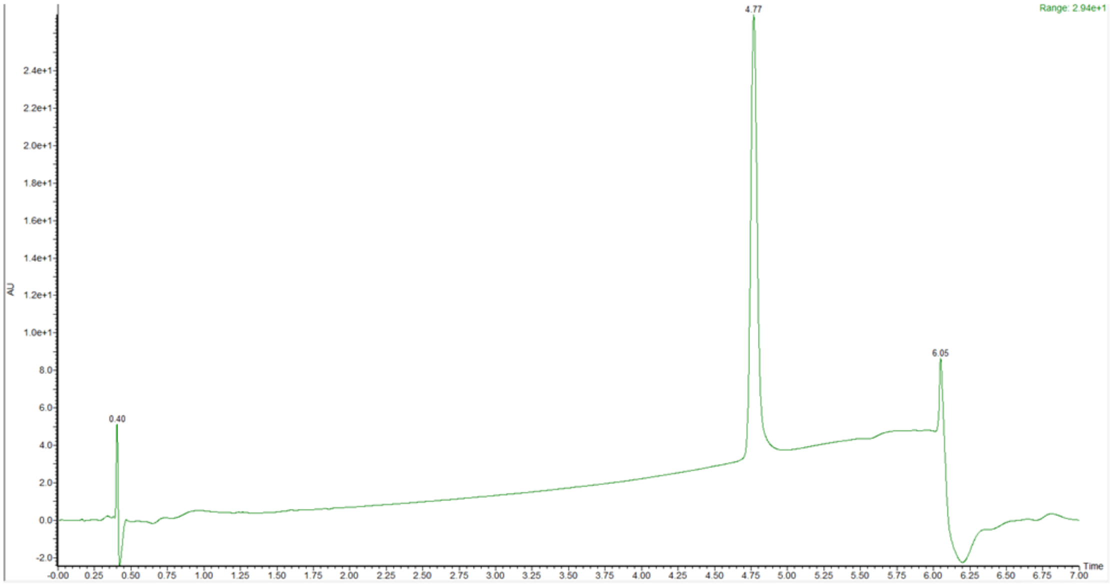


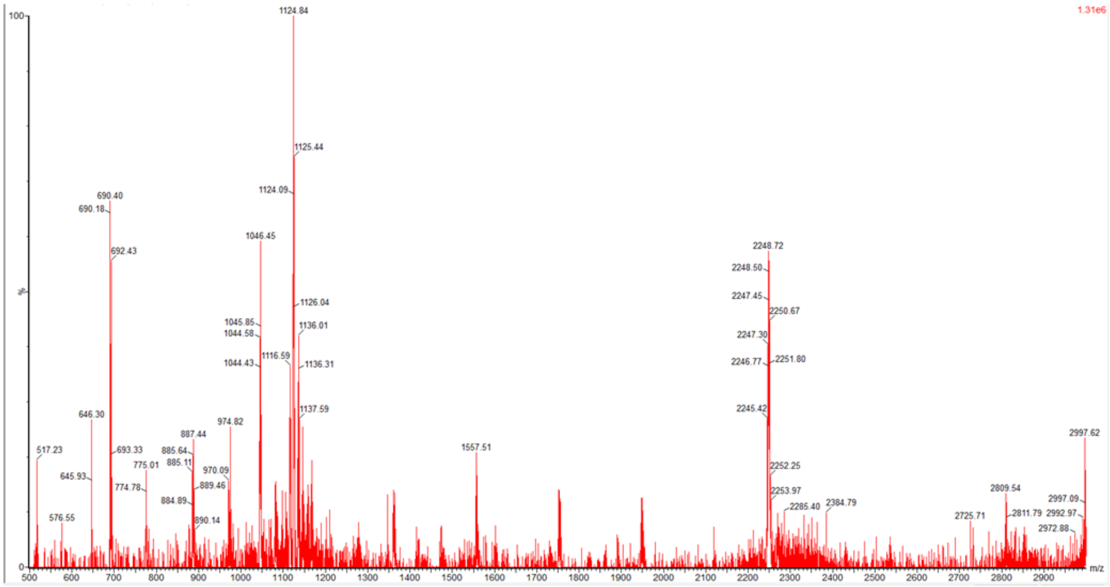


**Supplementary Figure 40 | UPLC-MS characterization of Pep-12.** (Top image) UPLC characterization of Pep-**12** with the gradient of 5 - 95% $CH_3CN$ in $H_2O$. (Bottom image) MS characterization of Pep-**12.**

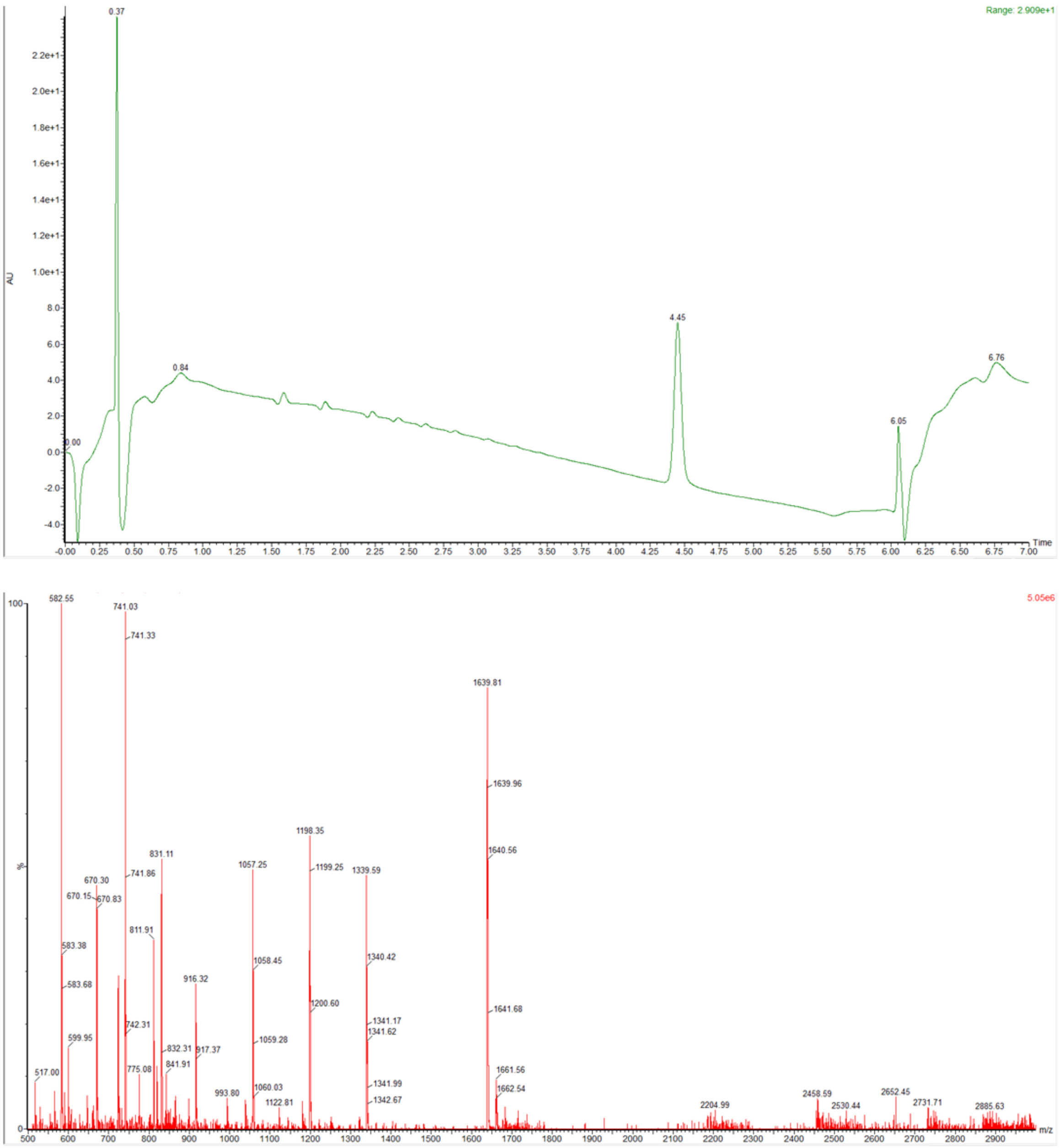


**Supplementary Figure 41 | UPLC-MS characterization of Pep-13.** (Top image) UPLC characterization of Pep-**13** with the gradient of 5 - 95% $CH_3CN$ in $H_2O$. (Bottom image) MS characterization of Pep-**13.**

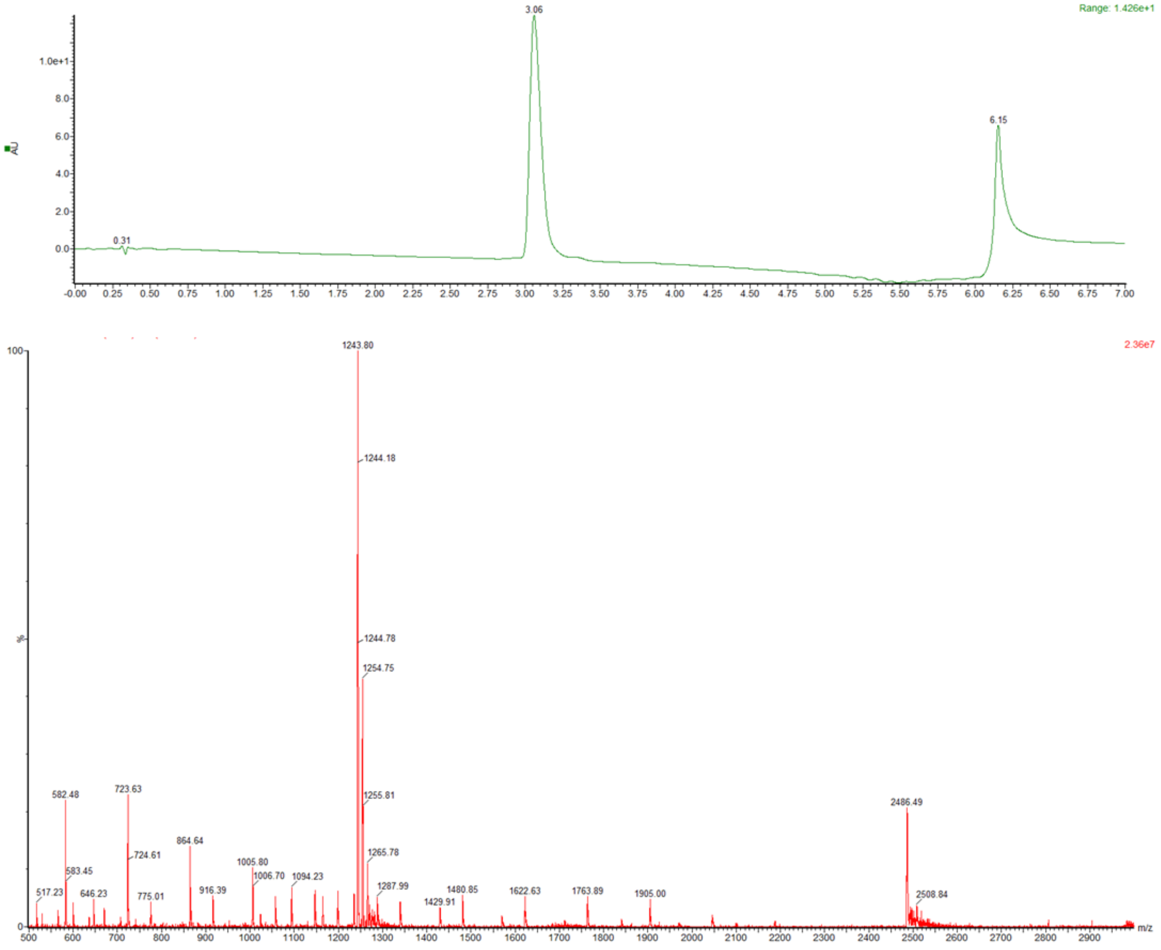


**Supplementary Figure 42 | UPLC-MS characterization of Pep-14.** (Top image) UPLC characterization of Pep-**14** with the gradient of 80 - 100% $CH_3CN$ in $H_2O$. (Bottom image) MS characterization of Pep-**14.**

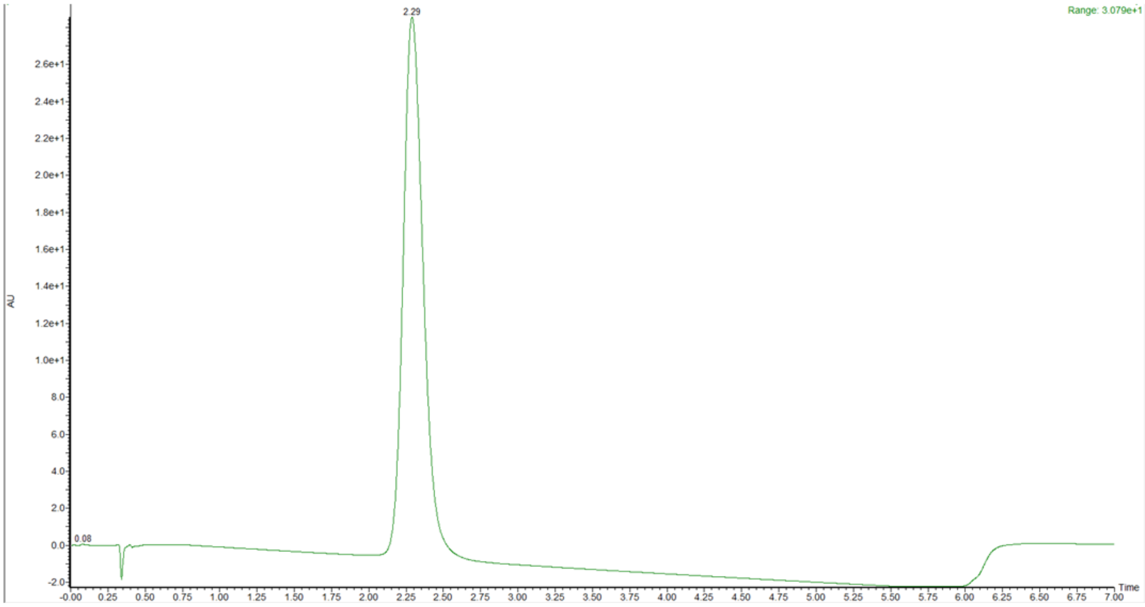


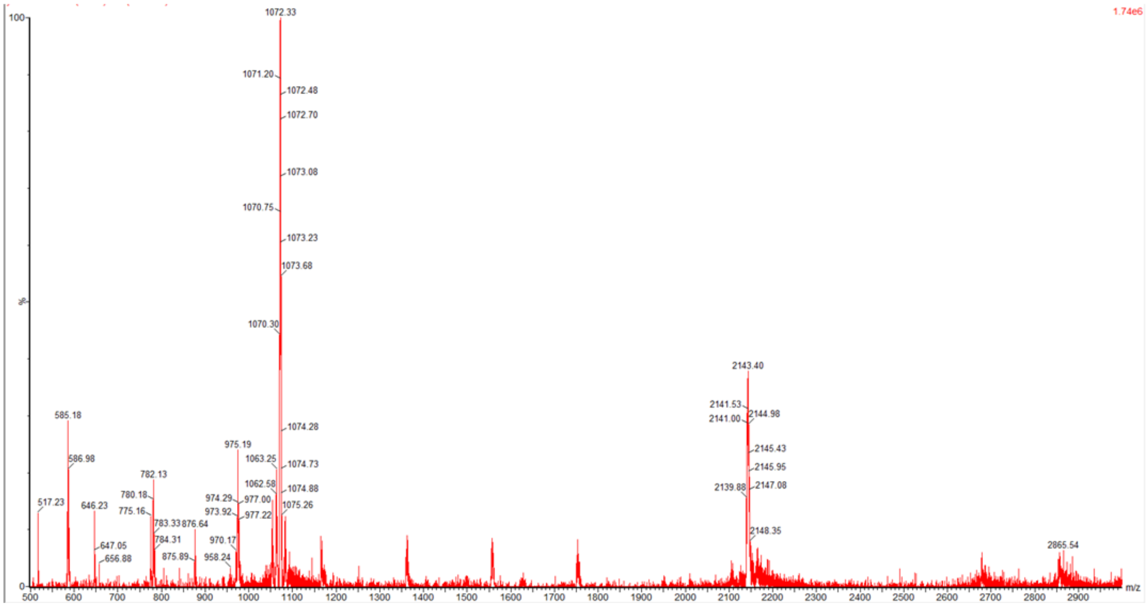


**Supplementary Figure 43 | UPLC-MS characterization of 1-Ntyr-Pep-2.** (Top image) UPLC characterization of 1-Ntyr-**Pep-2** with the gradient of 55 - 75% $CH_3CN$ in $H_2O$. (Bottom image) MS characterization of 1-Ntyr-**Pep-2.**

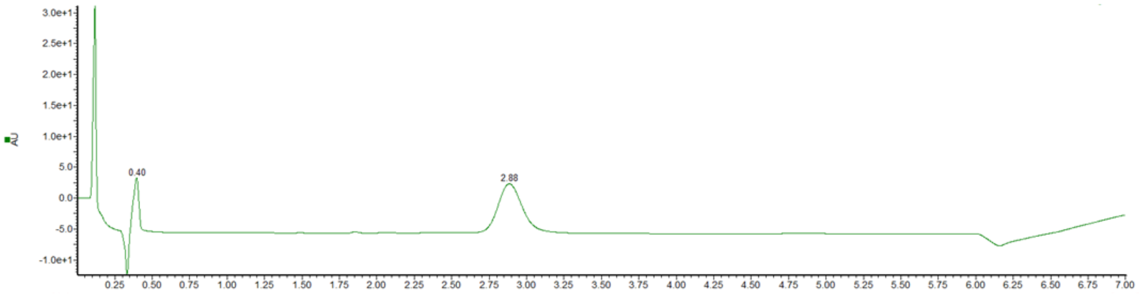


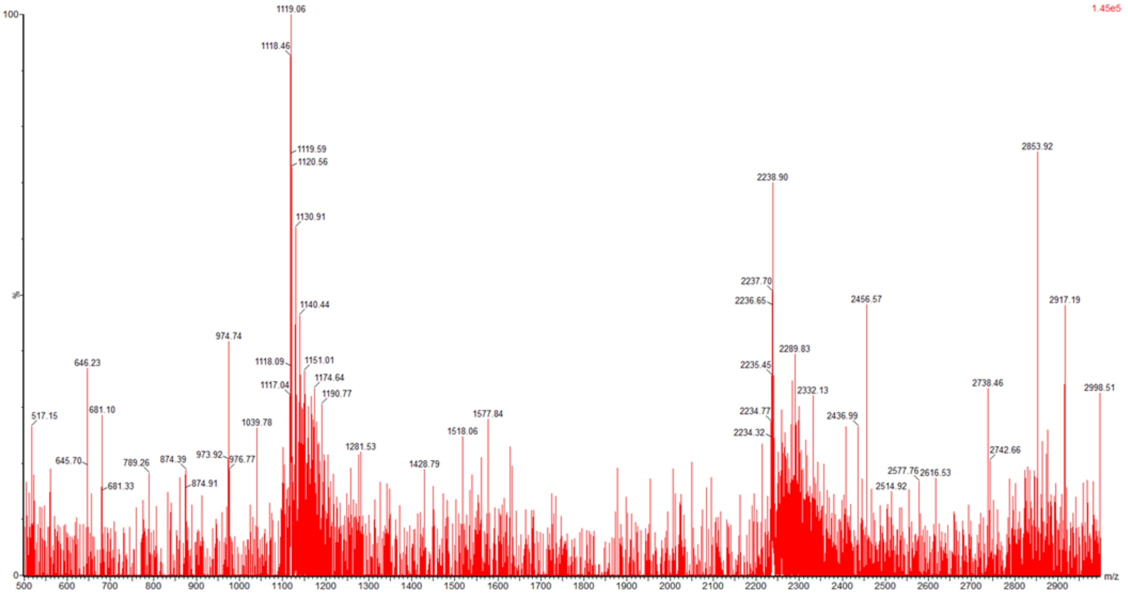


**Supplementary Figure 44 | UPLC-MS characterization of 1-Npyr-Pep-2.** (Top image) UPLC characterization of 1-Npyr-**Pep-2** with the gradient of 65 - 70% $CH_3CN$ in $H_2O$. (Bottom image) MS characterization of 1-Npyr-**Pep-2.**

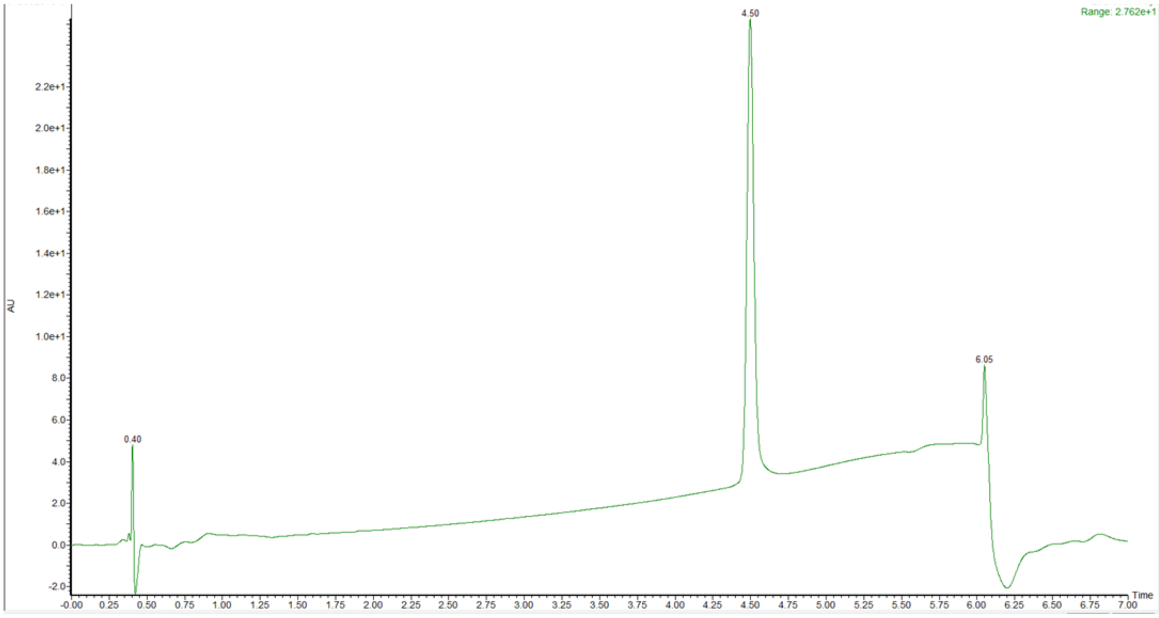


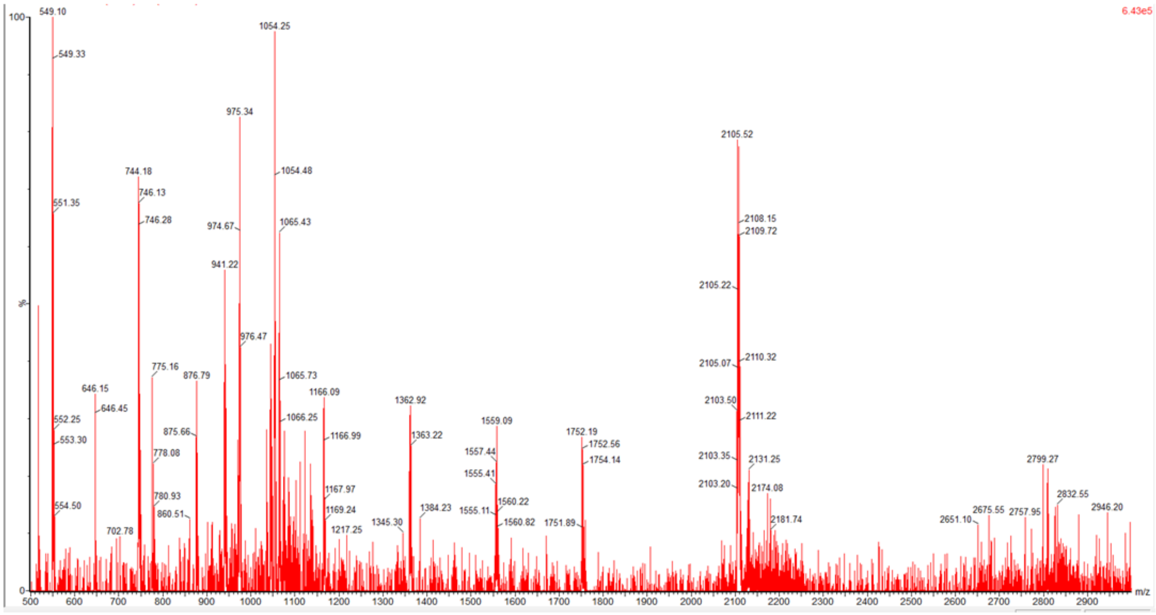


**Supplementary Figure 45 | UPLC-MS characterization of 1-Nhex-Pep-2.** (Top image) UPLC characterization of 1-Nhex-**Pep-2** with the gradient of 5 - 95% $CH_3CN$ in $H_2O$. (Bottom image) MS characterization of 1-Nhex-**Pep-2.**

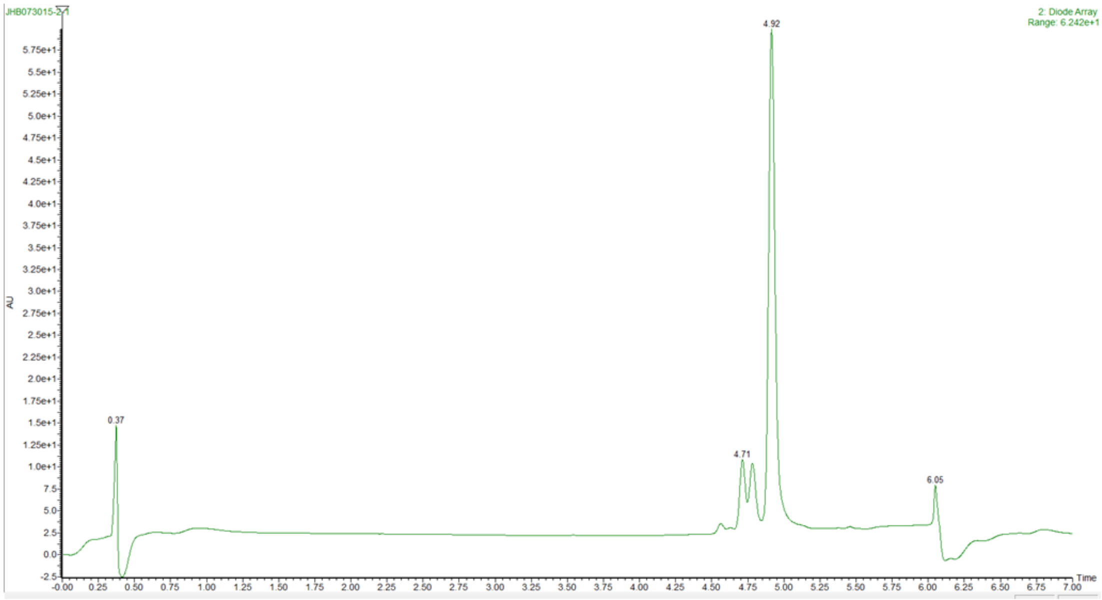


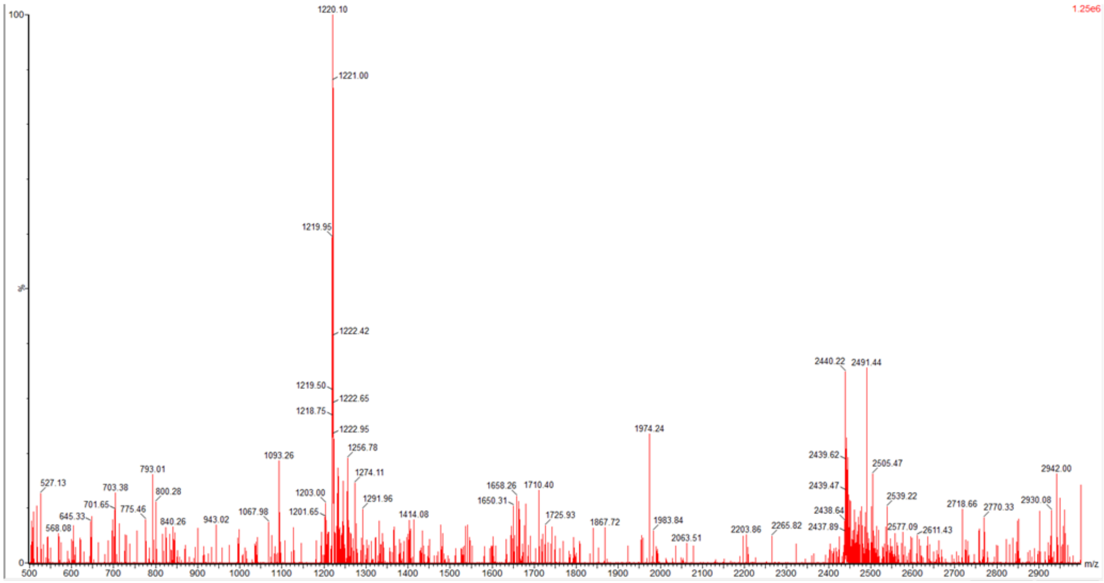


**Supplementary Figure 46 | UPLC-MS characterization of 1,8-(Nazo)$_2$-Pep-2.** (Top image) UPLC characterization of 1,8-(Nazo)$_2$-**Pep-2** with the gradient of 5 - 95% $CH_3CN$ in $H_2O$. (Bottom image) MS characterization of 1,8-(Nazo)$_2$-**Pep-2.**

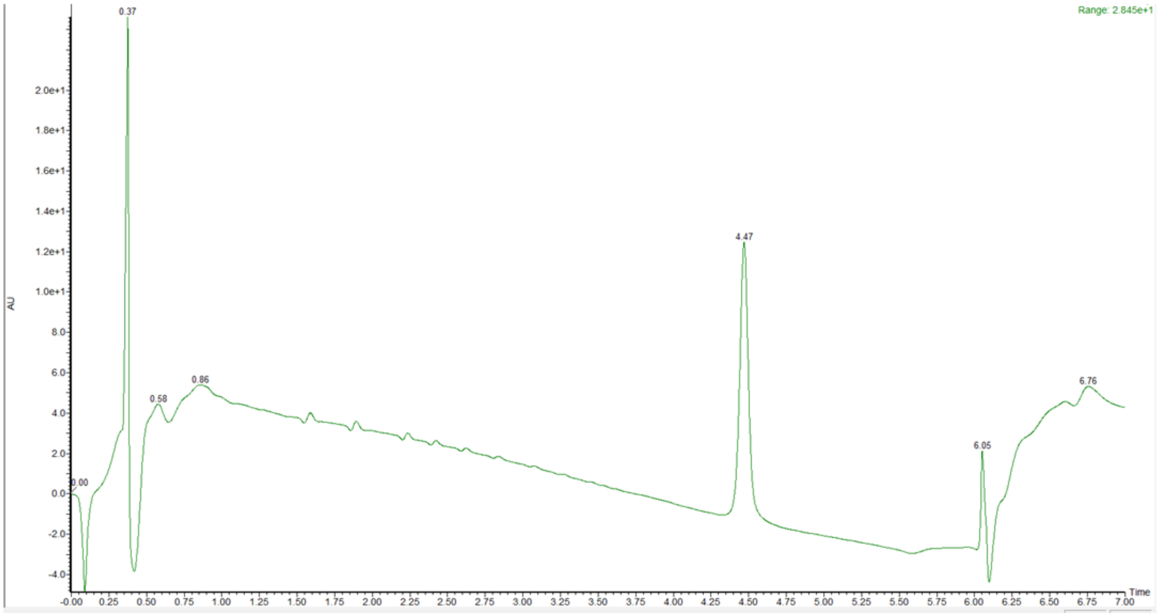


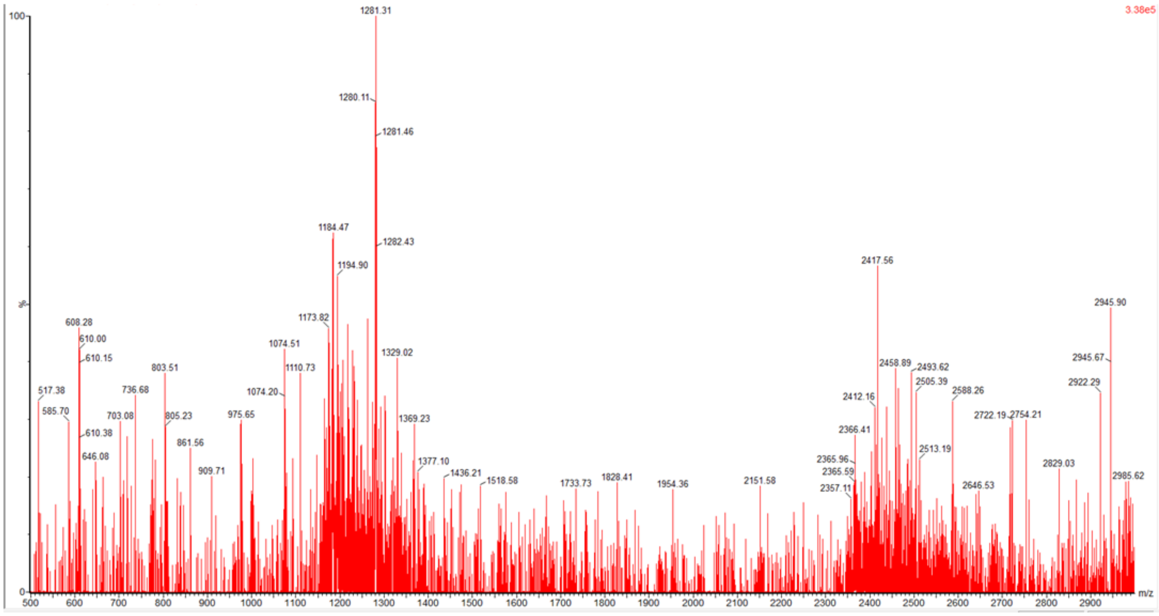


**Supplementary Figure 47 | UPLC-MS characterization of 1,8-(Ntrp)$_2$-Pep-2.** (Top image) UPLC characterization of 1,8-(Ntrp)$_2$-**Pep-2** with the gradient of 5 - 95% $CH_3CN$ in $H_2O$. (Bottom image) MS characterization of 1,8-(Ntrp)$_2$-**Pep-2.**

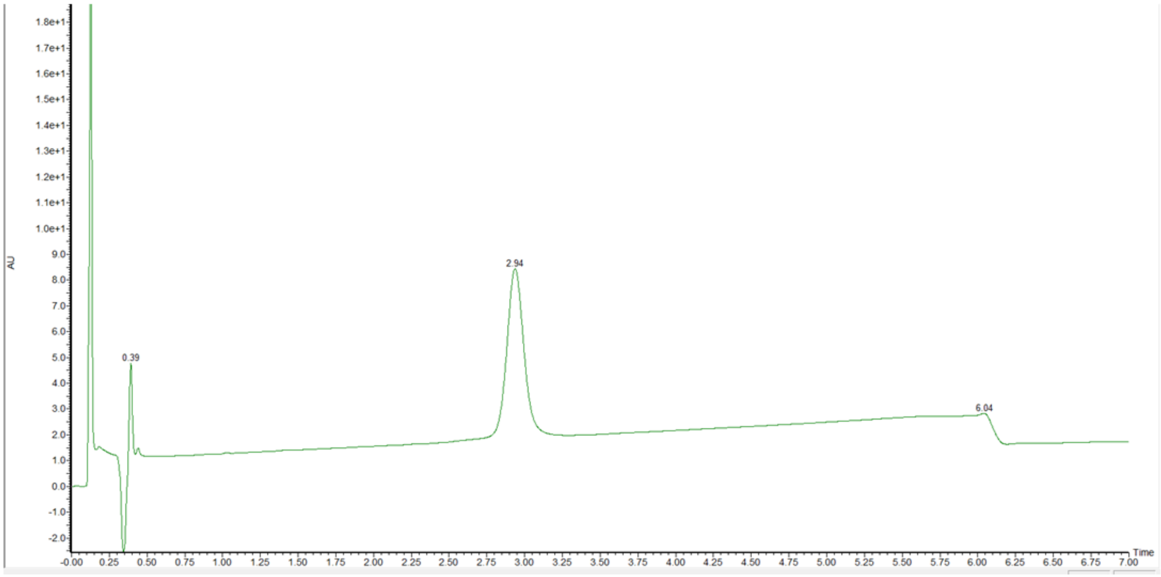


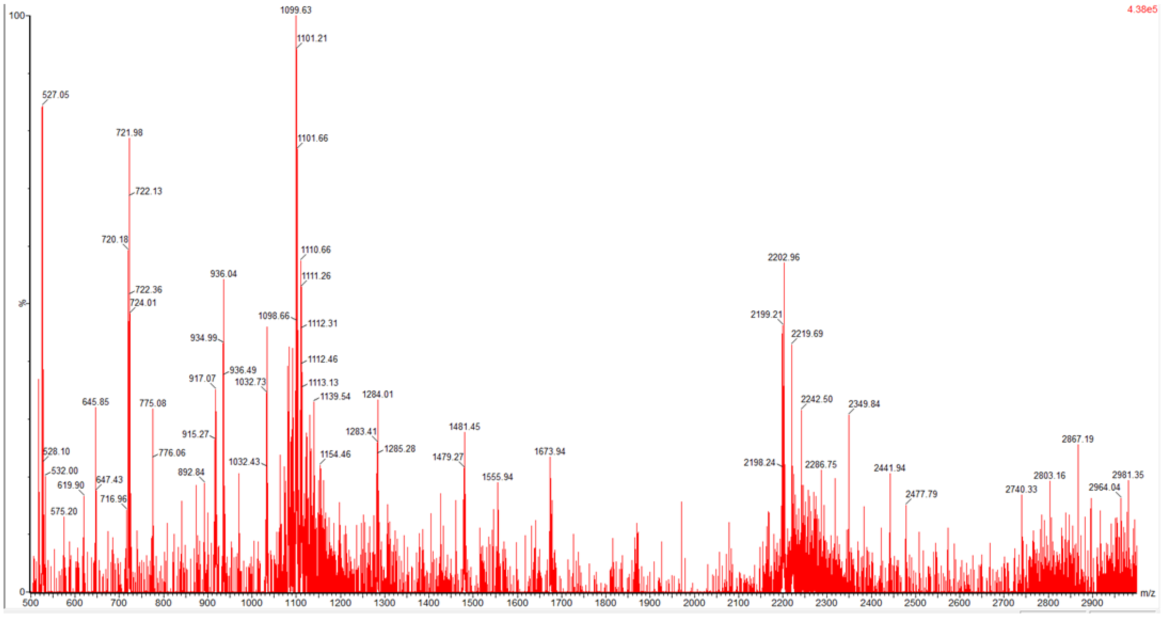


**Supplementary Figure 48 | UPLC-MS characterization of 1,8-(Nse)$_2$-Pep-2.** (Top image) UPLC characterization of 1,8-(Nse)$_2$-**Pep-2** with the gradient of 55 - 75% $CH_3CN$ in $H_2O$. (Bottom image) MS characterization of 1,8-(Nse)$_2$-**Pep-2.**

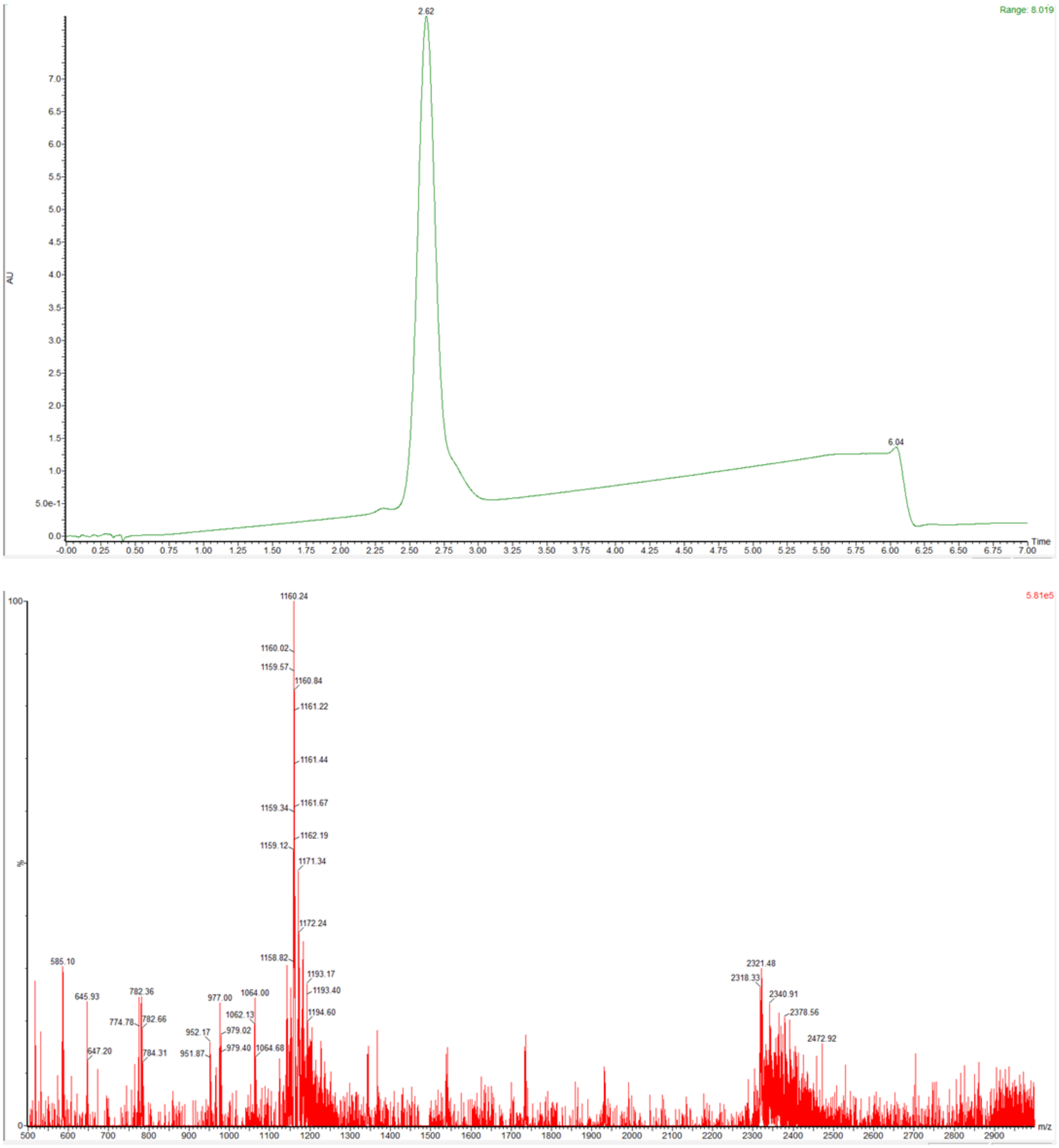


**Supplementary Figure 49 | UPLC-MS characterization of 1,8-(Ntyr)$_2$-Pep-2.** (Top image) UPLC characterization of 1,8-(Ntyr)$_2$-**Pep-2** with the gradient of 55 - 75% $CH_3CN$ in $H_2O$. (Bottom image) MS characterization of 1,8-(Ntyr)$_2$-**Pep-2.**

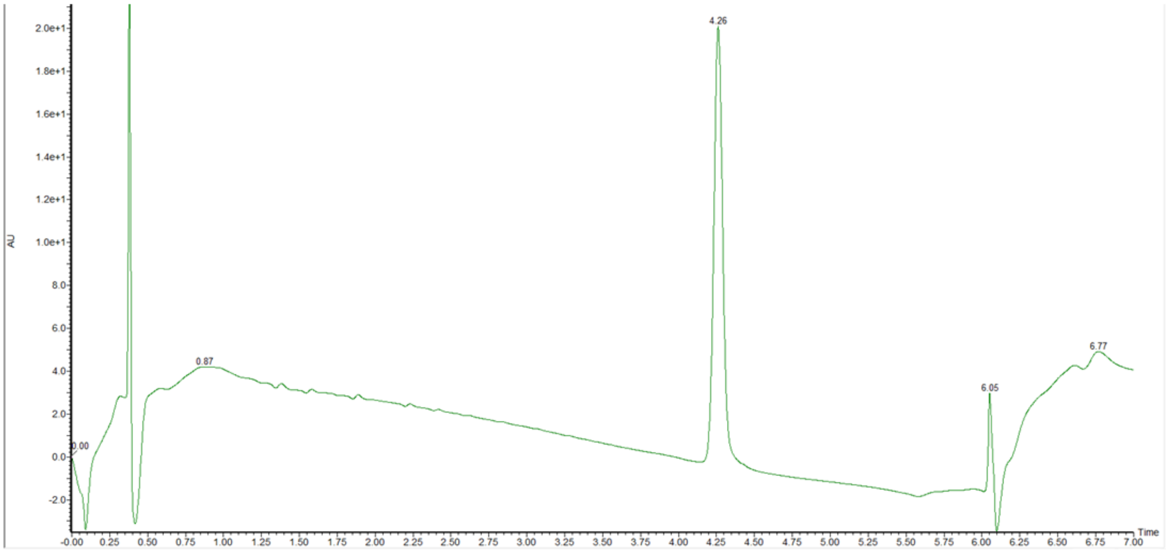


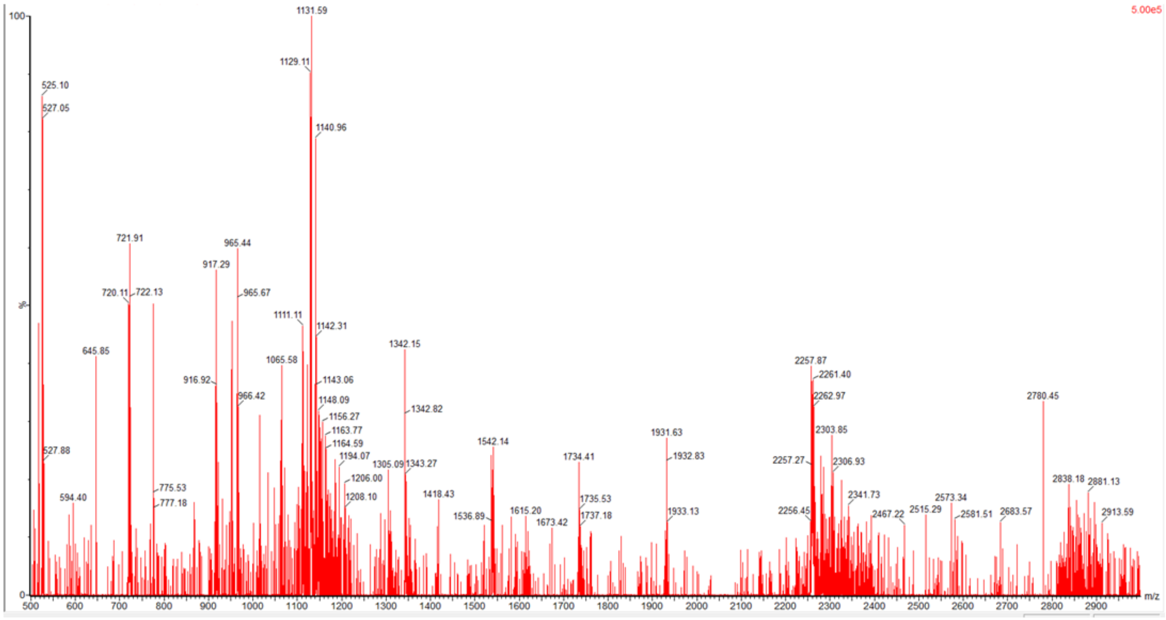


**Supplementary Figure 50 | UPLC-MS characterization of 1-Nse-8-Ntyr-Pep-2.** (Top image) UPLC characterization of 1-Nse-8-Ntyr-**Pep-2** with the gradient of 5 - 95% $CH_3CN$ in $H_2O$. (Bottom image) MS characterization of 1-Nse-8-Ntyr-**Pep-2.**

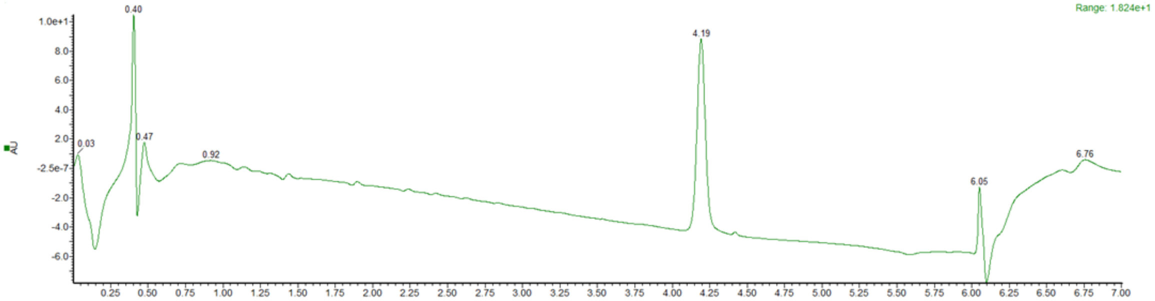


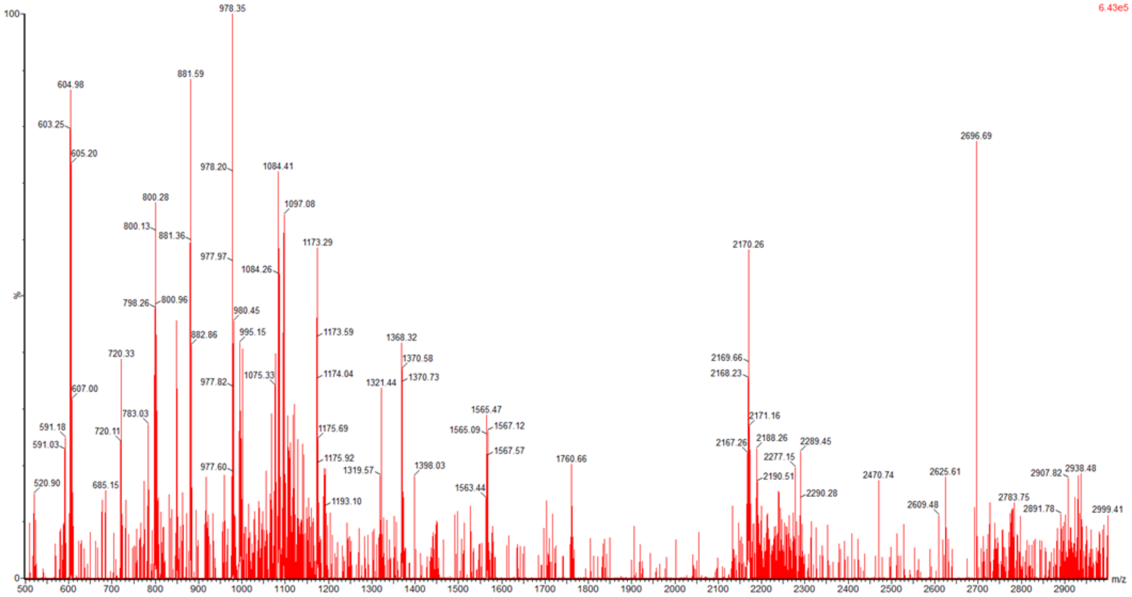


**Supplementary Figure 51 | UPLC-MS characterization of 13-Nte-Pep-2.** (Top image) UPLC characterization of 13-Nte-**Pep-2** with the gradient of 5 - 95% $CH_3CN$ in $H_2O$. (Bottom image) MS characterization of 13-Nte-**Pep-2.**

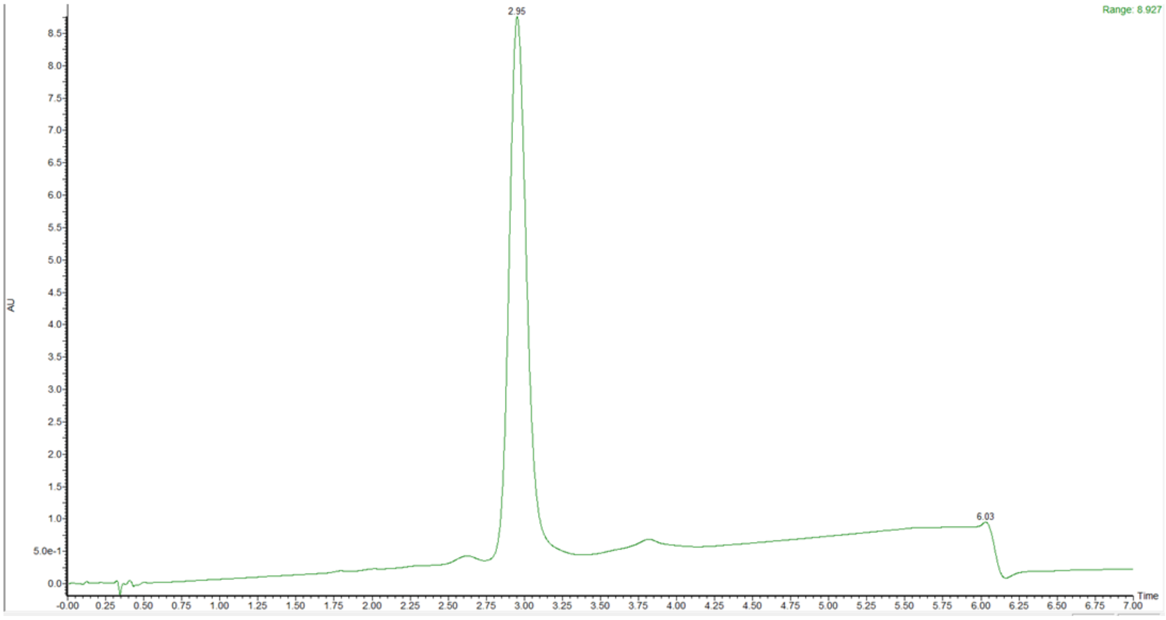


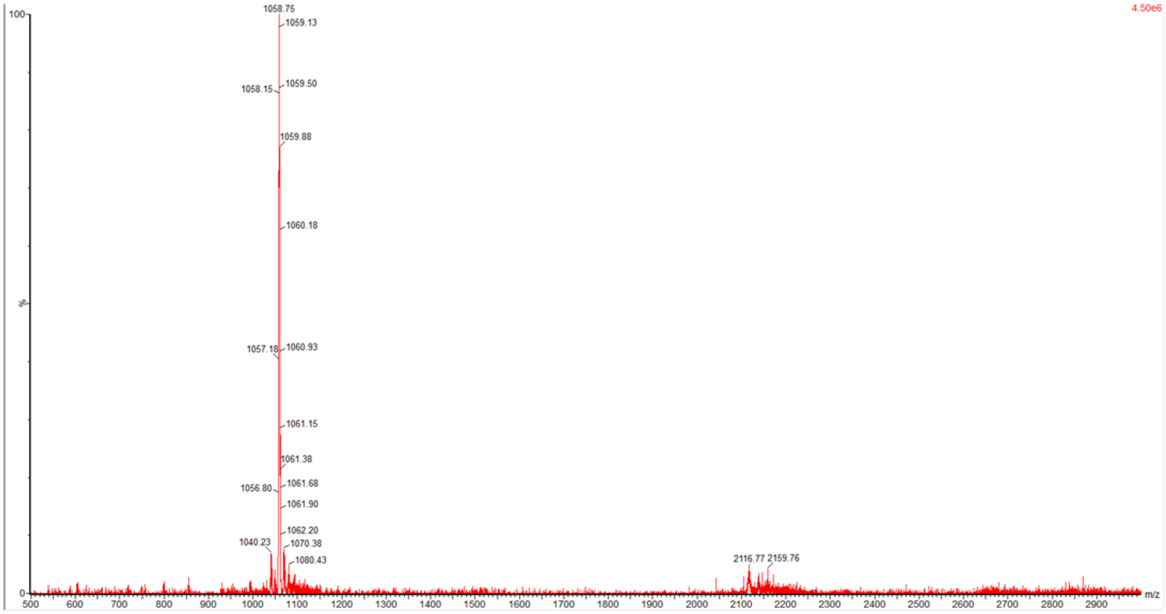


**Supplementary Figure 52 | UPLC-MS characterization of 13-Nhis-Pep-2.** (Top image) UPLC characterization of 13-Nhis-**Pep-2** with the gradient of 50 - 70% $CH_3CN$ in $H_2O$. (Bottom image) MS characterization of 13-Nhis-**Pep-2.**

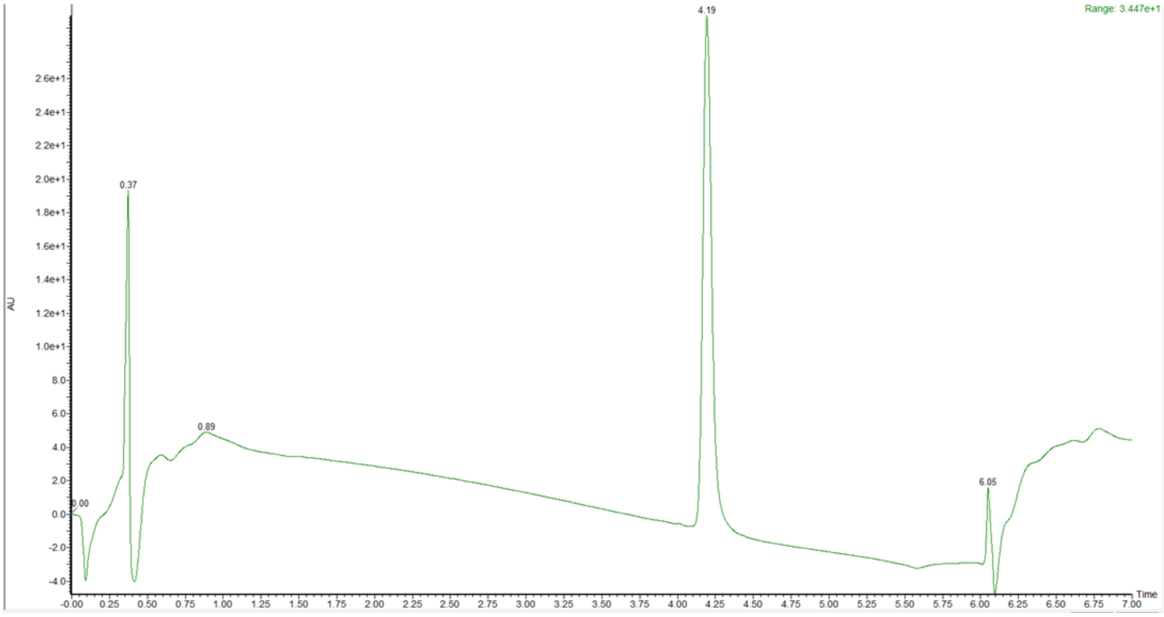


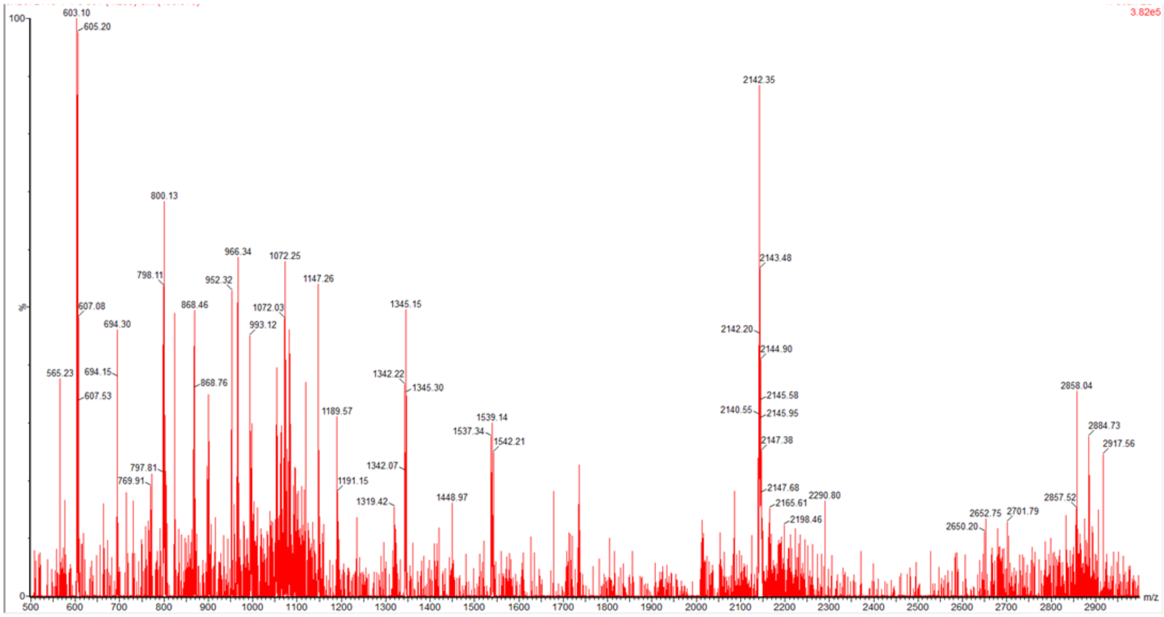


**Supplementary Figure 53 | UPLC-MS characterization of 13-Ntyr-Pep-2.** (Top image) UPLC characterization of 13-Ntyr-**Pep-2** with the gradient of 5 - 95% $CH_3CN$ in $H_2O$. (Bottom image) MS characterization of 13-Ntyr-**Pep-2.**

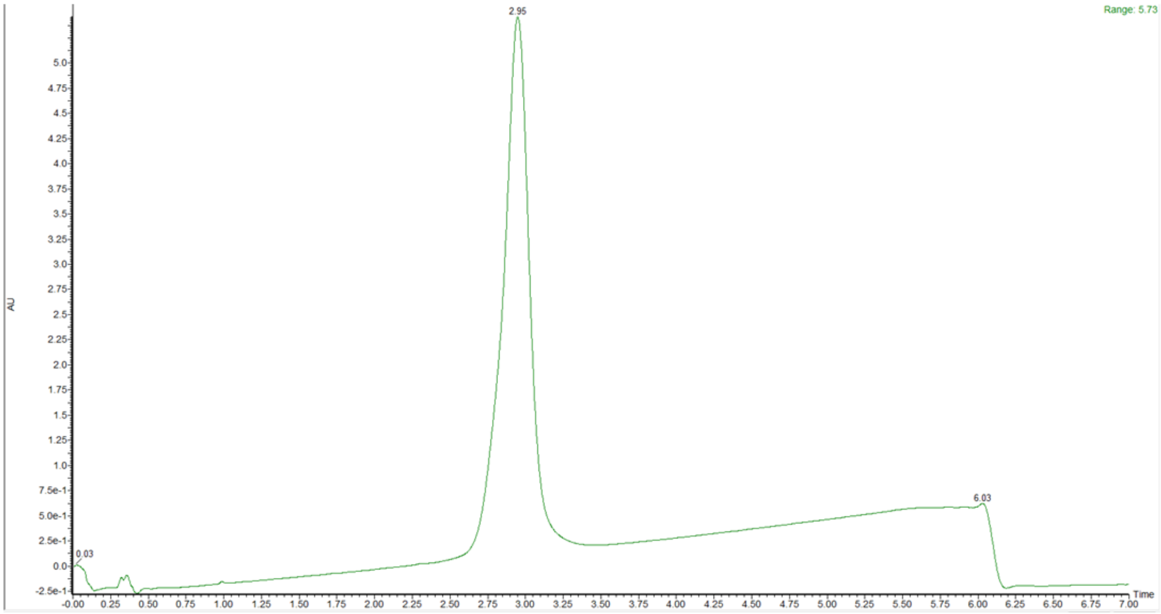


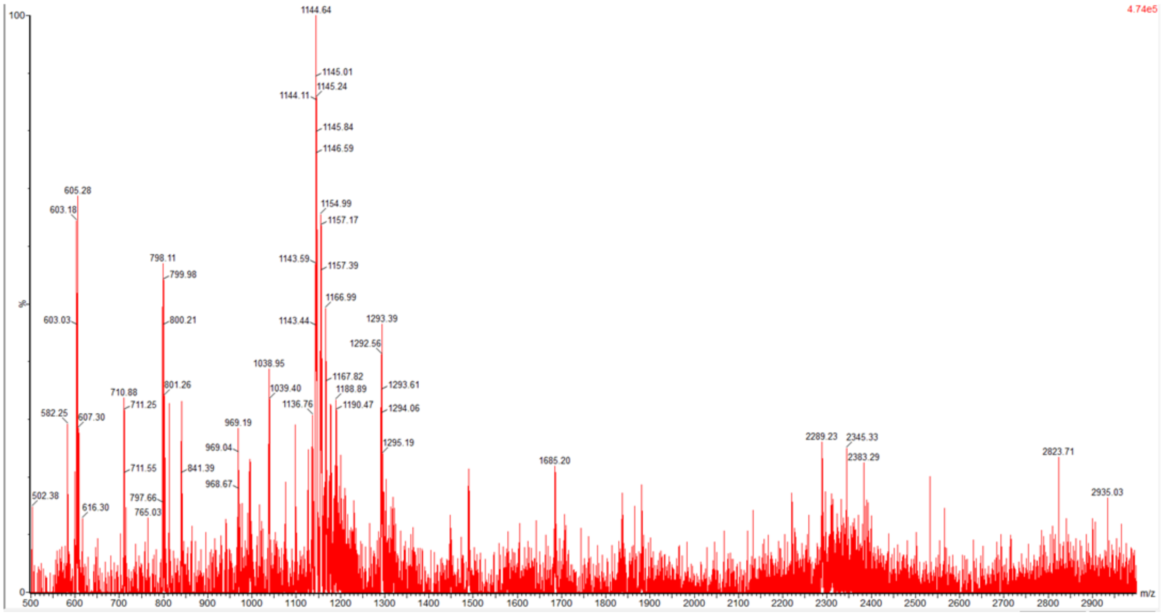


**Supplementary Figure 54 | UPLC-MS characterization of 13-Nbce-Pep-2.** (Top image) UPLC characterization of 13-Nbce-**Pep-2** with the gradient of 55 - 70% $CH_3CN$ in $H_2O$. (Bottom image) MS characterization of 13-Nbce-**Pep-2.**

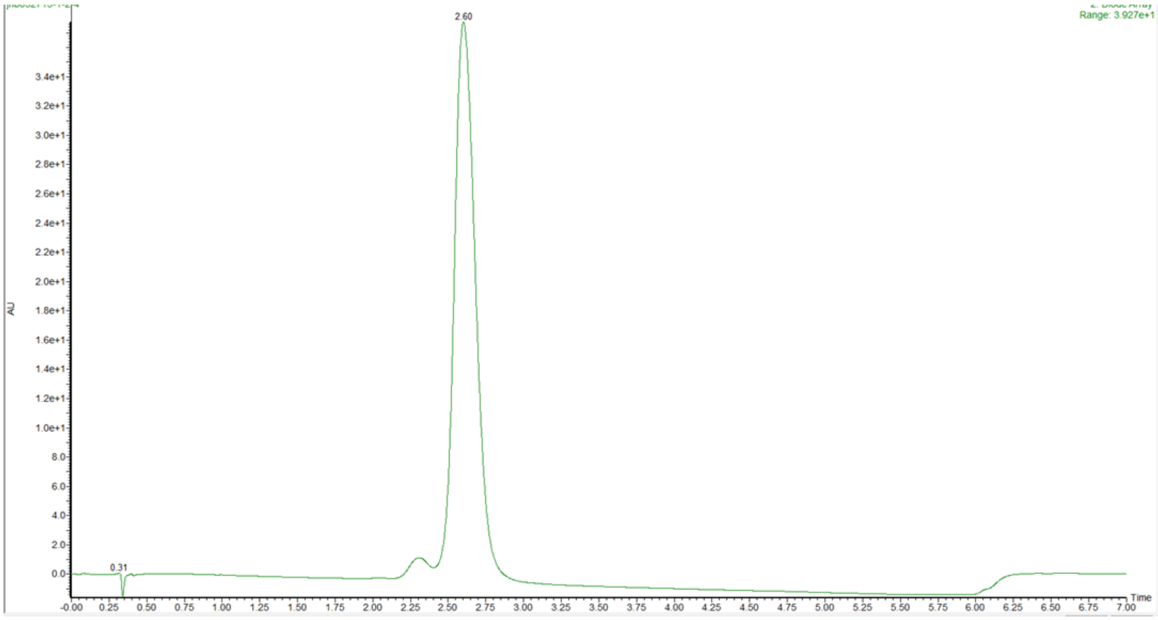


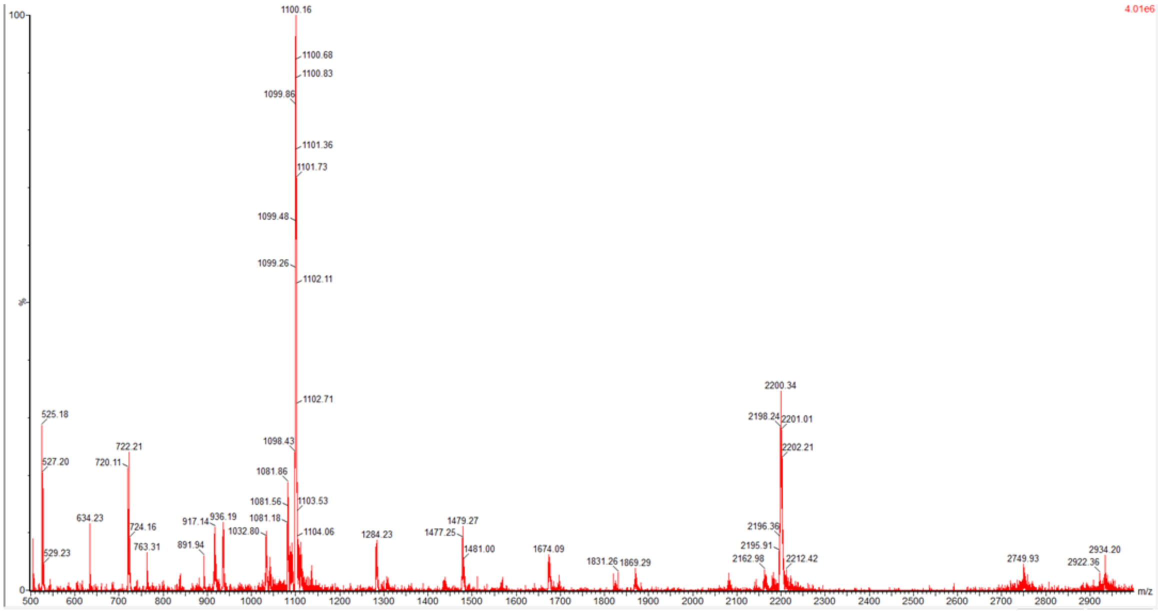


**Supplementary Figure 55 | UPLC-MS characterization of 1,14-(Nse)$_2$-Pep-2.** (Top image) UPLC characterization of 1,14-(Nse)$_2$-**Pep-2** with the gradient of 55 - 75% $CH_3CN$ in $H_2O$. (Bottom image) MS characterization of 1,14-(Nse)$_2$-**Pep-2.**

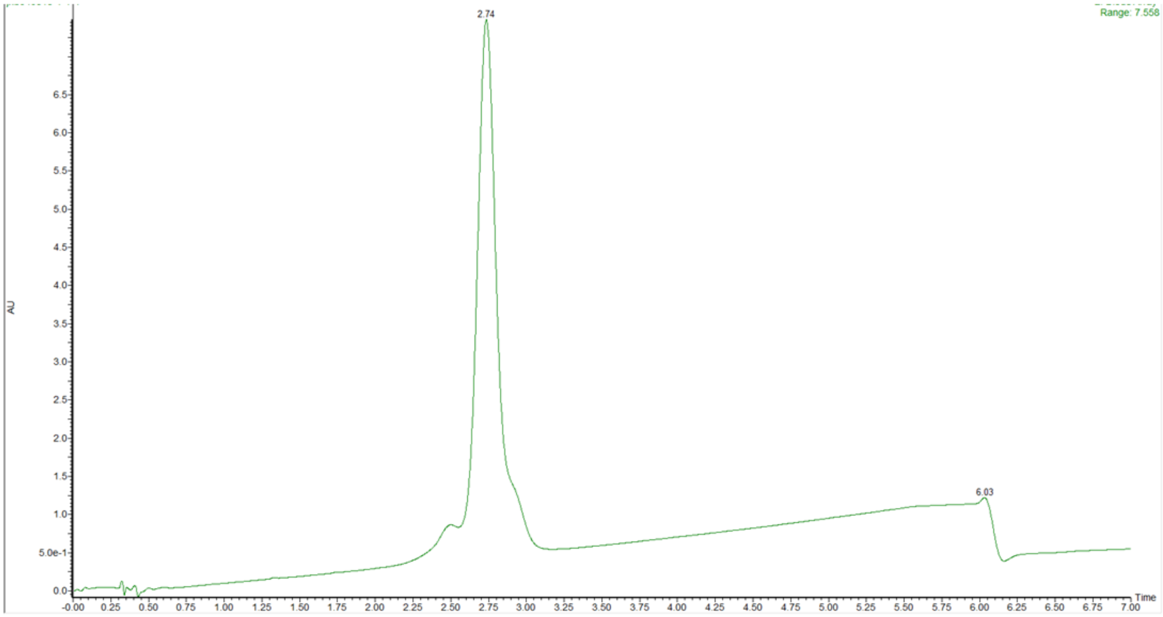


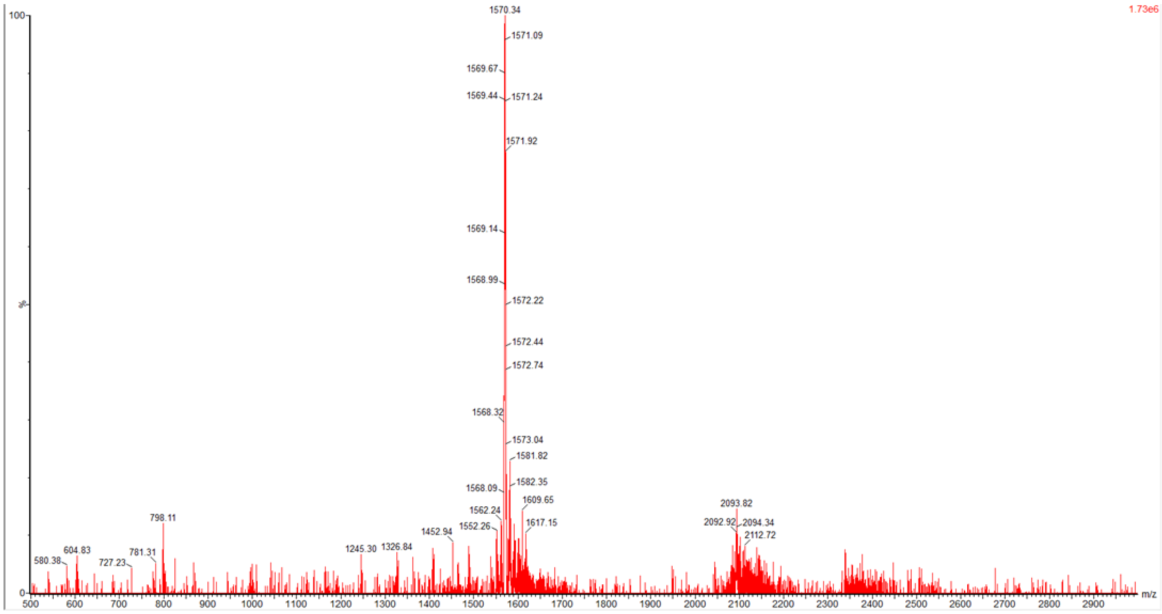


**Supplementary Figure 56 | UPLC-MS characterization of Ncd-Pep-2.** (Top image) UPLC characterization of Ncd-**Pep-2** with the gradient of 50 - 70% $CH_3CN$ in $H_2O$. (Bottom image) MS characterization of Ncd-**Pep-2.**

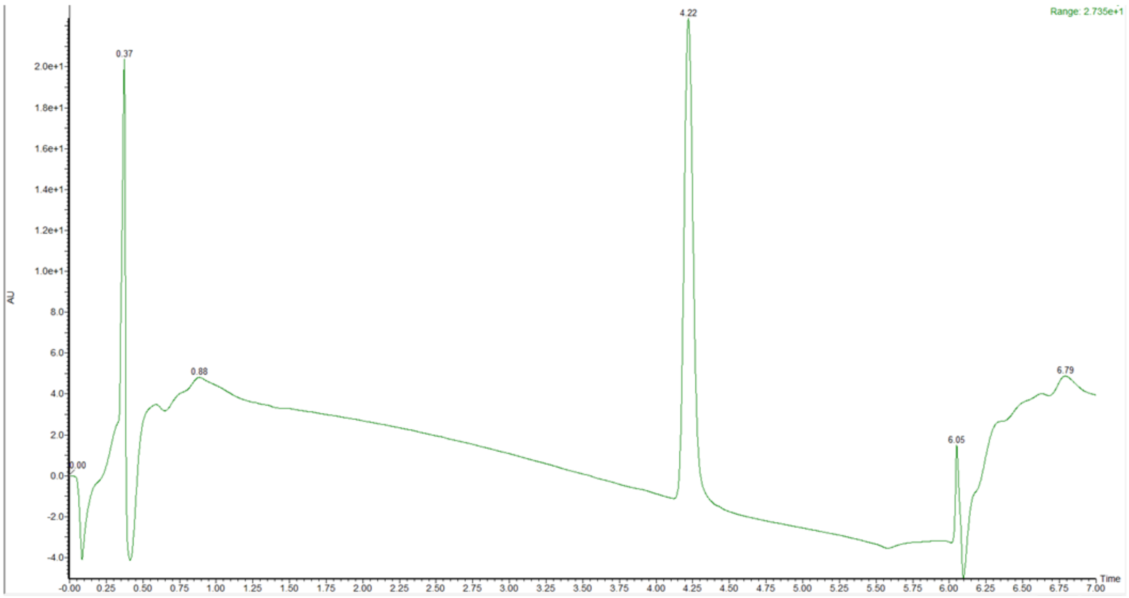


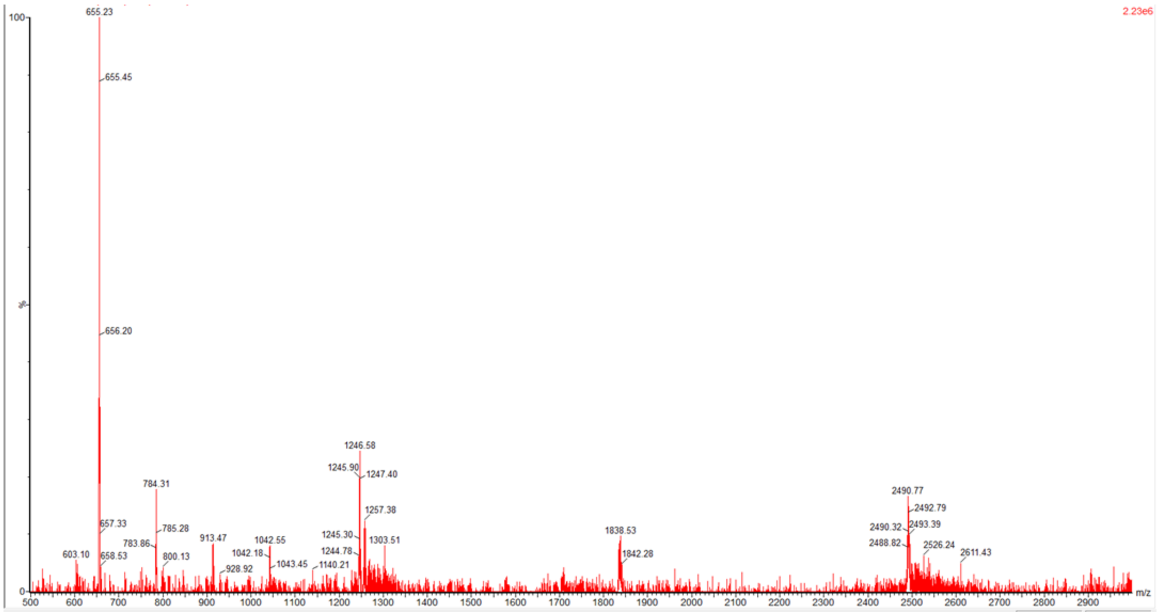


**Supplementary Figure 57 | UPLC-MS characterization of NHS-Rhodamine-labeled Pep-3.** (Top image) UPLC characterization of NHS-Rhodamine-labeled Pep-**3** with the gradient of 5 - 95% $CH_3CN$ in $H_2O$. (Bottom image) MS characterization of NHS-Rhodamine-labeled Pep-**3.**

# Supplementary Methods

## Materials

β-alanine tert-butyl ester hydrochloride was purchased from Chem-Impex International, Inc. was deprotected by the sodium hydroxide aqueous solution, then extracted with $CH_2Cl_2$, filtered and rotary evaporated for further reaction. *N,N'*-diisopropylcarbodiimide, bromoacetic acid and trifluoroactic acid (TFA) were purchased from Chem-Impex International, Inc. 2-(4-Chlorophenyl)ethylamine, cysteamine hydrochloride and 4-aminoazobenzene were purchased from VWR. 1-Pyrenemethylamine hydrochloride, chloroacetic acid, histamine, ammonium hydroxide solution (28.0-30.0% $NH_3$ basis), tyramine, tryptamine and *β*-cyclodextrin were purchased from Sigma-Aldrich. 4'-Aminobenzo-15-crown 5-Ether were purchased from TCI America. *p*-Toluenesulfonyl chloride and triphenylmethanol were purchased from Fisher Scientific. 2-(Tritylthio)ethanamine was synthesized according to the reported literature.[1] 2-(2-(2-methoxyethoxy)ethoxy)ethylamine was synthesized with the same protocol reported in the literature.[2] All other amine submonomers and other reagents are obtained from commercial sources and used without further purification.

## Synthesis of 2-(tritylthio)ethanamine

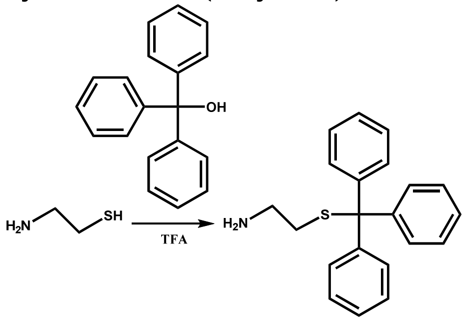


2-(tritylthio)ethanamine was synthesized according to the reported literature.[1] For the detail synthesis route, cysteamine hydrochloride (1.931 g, 17 mmol) was dissolved in the 8 mL of TFA. Triphenylmethanol (4.426 g, 17 mmol) were added, reacting for 40 minutes at room temperature. TFA was evaporated under a stream of nitrogen gas, and the resulted residue was triturated with ethyl ether. The white precipitate was filtered and then partitioned with aqueous NaOH (25 mL, 1M). Finally, the crude product solution was extracted with ethyl acetate. The organic layer was dried with anhydrous $Na_2SO_4$. The white solid product were obtained after the rotary evaporation and dried in a vacuum drying oven at 40 $^{o}$C for 24 hours (4.408 g, 13.8 mmol, Yield: 81%; MS *m/z*: (positive ion ESI) 639.15 for $[2M]^+$.

## Synthesis of mono-6-amino-6-deoxy-cyclodextrin ($CD\text{-}NH_2$)

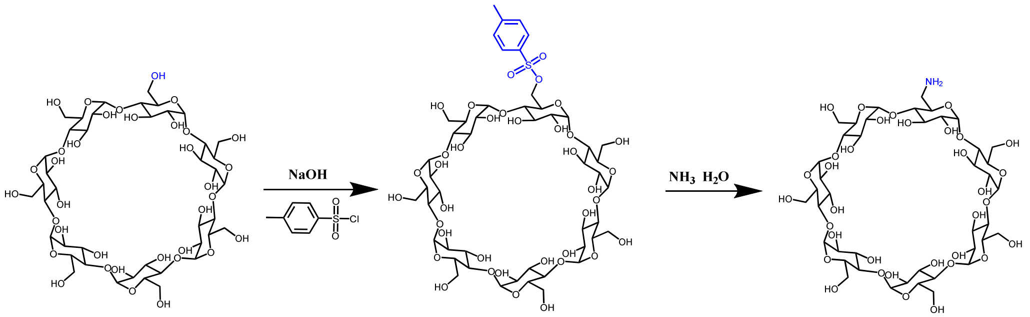


In the first step, mono-6-(*p*-toluenesulfonyl)-6-deoxy-cyclodextrin (CD-Ts) was synthesized according to the reported literature.[3] *β*-Cyclodextrin (50 g, 44.1 mmol) was dissolved in the 300 mL of deioned water, immersed in the 0 $^{o}$C ice bath. NaOH (5.475 g, 137 mmol) was added, leading to the complete dissolution of *β*-cyclodextrin. *p*-Toluenesulfonyl chloride (8.4 g, 44.1 mmol) dissolved in 30 mL of acetonitrile was step-wisely dropped into the solution. After that, the mixture was reacting 3 hours at room temperature. pH value of the mixture was adjusted to about 9.0, putting in the 4 $^{o}$C fridge overnight. The crude product was filtered and dried in a vacuum drying oven at 50 $^{o}$C for 2 days. The product of CD-Ts was obtained as white solid (10.977 g, 8.5 mmol, Yield: 19.3%).

CD-Ts (4.723 g, 3.66 mmol) was added into the single-neck round-bottom flask. 50 mL of ammonium hydroxide solution was added to complete dissolve the sample, reacting for 3 days at room temperature. The crude product was precipitated in the ice cold acetone for 3 times and dried in a vacuum drying oven at 30 $^{o}$C for 24 hours. The final product of CD-$NH_2$ was white solid (4.040g, 3.56 mmol, Yield: 97%), MS *m/z*: (positive ion ESI) 1134.29 for [$M^+$].

## Peptoid synthesis

### Methods for the automated solid-phase synthesis

Lipid-like peptoids were synthesized on a commercial Aapptec Apex 396 robotic synthesizer using a modified solid-phase submonomer synthesis method as described previously.[4,5] Rink amide resin (0.09 mmol) was used to generate C-terminal amide peptoids. In this method, the Fmoc group on the resin was deprotected by adding 2 mL of 20% (v/v) 4-Methylpiperidine/N,Ndimethylformamide (DMF), agitating for 20 min, draining, and washing with DMF. All DMF washes consisted of the addition of 1.5 mL of DMF, followed by agitation for 1 min (repeated five times). An acylation reaction was then performed on the amino resin by the addition of 1.6 mL of 0.6 M bromoacetic acid in DMF, followed by 0.35 mL of 50% (v/v) N,N-diisopropylcarbodiimide (DIC)/DMF. The mixture was agitated for 30 minutes at room temperature, drained, and washed with DMF for 5 times. Nucleophilic displacement of the bromide with various primary amines occurred by a 1.6 mL addition of the primary amine monomer as a 0.6 M solution in N-methyl-2-pyrrolidone (NMP), followed by agitation for 60 minutes at room temperature. The monomer solution was drained from the resin, and the resin was washed with DMF for 5 times. The acylation and displacement steps were repeated until a lipid-like peptoid of the desired length was synthesized.

### Methods for the manual synthesis

Rink amide resin (0.09 mmol) was used to generate C-terminal amide peptoids. In the synthesis procedure, the Fmoc groups on the resin were deprotected by adding 2 mL of 20% (v/v) 4-methylpiperidine/N,N-dimethylformamide (DMF), agitating for 40 min, filtering, and washing with DMF. For all DMF washes, 1 mL DMF was added and then agitated for 1 min (repeated five times). An acylation reaction was then performed on the amino resin by the addition of 1.5 mL of 0.6 M bromoacetic acid in DMF, followed by adding 0.30 mL of 50% (v/v) N,N-diisopropylcarbodiimide (DIC)/DMF. The mixture was agitated for 10 minutes at room temperature, filtered and washed with DMF for 5 times. Nucleophilic displacement of the bromine with different primary amines occurred by the addition of 1.5 mL of 0.6 M primary amine monomerin N-methyl-2-pyrrolidone (NMP), followed by the agitation for 10 minutes at room temperature. The monomer solution were filtered from the resin, and washed with DMF for 5 times. The acylation and displacement steps were repeated until the designed peptoid was synthesized.

**Methods for synthesizing other uncommon sequences**

**Synthesis of Pep-3:** The resulting rink amide resins (0.09 mmol) containing Pep-**2** obtained from automated solid-phase synthesis were mixed with a DMF solution of Fmoc-6-aminohexanoic acid (1.5 mL, 0.9 mmol) and 0.50 mL of 50% (v/v) *N*,*N*-diisopropylcarbodiimide (DIC)/DMF. The mixture was agitated overnight at room temperature, filtered, and washed well with DMF. The terminal Fmoc group was deprotected by adding 2 mL of 20% (v/v) 4-methylpiperidine/DMF. The mixture was agitated for 40 min, filtered, and washed well with DMF.

**Synthesis of Ncd-Pep-2:** The resulting rink amide resins (0.09 mmol) containing Pep-**2** obtained from automated solid-phase synthesis were mixed with a DMF solution of bromoacetic acid (1.5 mL, 0.9 mmol) and 0.30 mL of 50% (v/v) N,N-diisopropylcarbodiimide (DIC)/DMF. The mixture was agitated for 10 minutes at room temperature, filtered and washed with DMF for 5 times. In the nucleophilic displacement step, 1.5 mL of 0.3 M CD-$NH_2$ in DMF and $K_2CO_3$ (100 mg, 0.72 mmol) were added, followed by the agitation for 3 days at 40 $^oC$. The monomer solution were filtered from the resin, washed with deionized water for 5 times, and then washed well with DMF.

**Synthesis of peptoids containing *N*-[4-(2-phenyldiazenyl)phenyl]glycines] (Nazo)**

**13-Nazo-Pep-2**: Rink amide resins (0.09 mmol) containing Pep-**2** were mixed with 1.5 mL of 0.6 M bromoacetic acid in DMF, followed by adding 0.30 mL of 50% (v/v) N,N-diisopropylcarbodiimide (DIC)/DMF. The mixture was agitated for 10 minutes at room temperature, filtered and washed with DMF. In the nucleophilic displacement step, a NMP solution of 4-Aminoazobenzene (1.5 mL, 0.9 mmol) and tetrabutylammonium iodide (TBAI, 100 mg, 0.27mmol) was added into the above resins, followed by the agitation for 2 days at 40 $^oC$. The resulting resins were first washed with deionized water for 5 times and then washed with DMF for 5 times.

**Synthesis of peptoids containing *N*-[benzo-15-crown-5-ether]glycines (Nbce)**

**13-Nbce-Pep-2**: Similar to the synthesis of **13-Nazo-Pep-2**, in the nucleophilic displacement step, a NMP solution 4'-Aminobenzo-15-crown 5-Ether (1.5 mL, 0.9 mmol) and tetrabutylammonium iodide (TBAI, 100 mg, 0.27mmol) were used, followed by the

agitation for 2 days at 40 °C. The resulting resins were first washed with deionized water for 5 times and then washed with DMF for 5 times.

**Synthesis of peptoids containing [2-(4-imidazolyl)ethylamine]glycines (Nhis)**

**13-Nhis-Pep-2:** Rink amide resins (0.09 mmol) containing Pep-**2** were mixed with 1.5 mL of 0.6 M chloroacetic acid in DMF, followed by adding 0.30 mL of 50% (v/v) N,N-diisopropylcarbodiimide (DIC)/DMF. The mixture was agitated for 10 minutes at room temperature, filtered and washed with DMF. In the nucleophilic displacement step, a NMP solution of histamine (1.5 mL, 0.9 mmol) was added into the above resins, followed by the agitation for one hour at 40 °C. The resulting resins were washed well with DM.

*Notes: After introducing Nhis in the peptoid, chloroacetic acid was used instead of bromoacetic acid for all subsequent steps of acylation in order to reduce side product formation as described previously.[6] In the displacement step, primary amines substituted the chloride atom under the condition of agitation 1 hour at 40 °C.*

**Synthesis of peptoids containing N-[2-(1H-indol-3-yl)ethyl]glycine (Ntrp)**

**13-Ntrp-Pep-2:** Rink amide resins (0.09 mmol) containing Pep-**2** were mixed with 1.5 mL of 0.6 M chloroacetic acid in DMF, followed by adding 0.30 mL of 50% (v/v) N,N-diisopropylcarbodiimide (DIC)/DMF. The mixture was agitated for 10 minutes at room temperature, filtered and washed well with DMF. In the nucleophilic displacement step, a NMP solution of tryptamine (1.5 mL, 0.9 mmol) was added into the above resins, followed by the agitation for one hour at 40 °C. The resulting resins were washed with DMF for 5 times.

*Notes: After introducing Ntrp in the peptoid, chloroacetic acid was used instead of bromoacetic acid for all subsequent steps of acylation in order to reduce side product formation as described previously.[6] In the displacement step, primary amines substituted the chloride atom under the condition of agitation 1 hour at 40 °C.*

**Synthesis of peptoids containing N-[(1-pyrenemethyl)]glycines (Npyr)**

**1-Npyr-Pep-2:** Rink amide resins (0.09 mmol) containing Pep-**2** were mixed with 1.5 mL of 0.6 M bromoacetic acid in DMF, followed by adding 0.30 mL of 50% (v/v) N,N-diisopropylcarbodiimide (DIC)/DMF. The mixture was agitated for 10 minutes at room temperature, filtered and washed with DMF for 5 times. In the nucleophilic displacement step, a 3.0 mL methanol solution of 1-Pyrenemethylaminehydrochloride (0.9 mmol) and N,N-Diisopropylethylamine (DIPEA) (0.9 mmol) was added into the above resins, followed by the agitation for 30 minutes at room temperature. The resulting resins were washed with DMF for 5 times.

**Synthesis of NHS-Rhodamine-labeled Pep-3:**

After the synthesis of **Pep-3**, 2 mL of DMF solution of NHS-Rhodamine (0.9 mmol) and 0.50 mL of 50% (v/v) N,N-diisopropylcarbodiimide (DIC)/DMF were added and mixed with resins containing **Pep-3**, followed by the agitation for overnight at room temperature. The monomer solution were filtered from the resin, and washed with DMF for 5 times.

**Peptoid cleavage and HPLC purification**

For most of lipid-like pepotids, their final crude products were obtained by cleaving the corresponding resins with addition of 95% trifluoroacetic acid (TFA) in water. For peptoids containing *N*-(2-thiolethyl)glycine (Nse) side chains, their crude products were cleaved from the corresponding resins with the addition of a solution containing 90% TFA, 5% triisopropylsilane and 5% water. TFA was then evaporated under a stream of $N_2$ gas. Finally, crude peptoids were dissolved in $H_2O/CH_3CN$ (v/v=1:1) for HPLC purification.

All peptoids were purified by reverse-phase HPLC on a XBridge™ Prep C18 OBD™ column (10 µm, 19 mm ×100 mm), using a narrow gradient of acetonitrile in $H_2O$ with 0.1% TFA over 15 min. Purified peptoids were analyzed using Waters ACQUITY reverse-phase UPLC (the corresponding gradient at 0.4 mL/min over 7 min at 40°C with a ACQUITY®BEH C18, 1.7 µm, 2.1 mm × 50 mm column) that was connected with a Waters SQD2 mass spectrometry system (See Supplementary Figs 29 – 57). The final peptoid product was lyophilized from its solution in a mixture (v/v = 1:1) of water and acetonitrile. The peptoid powder was finally divided into small portions (1.0 or 2.0$\times 10^{-6}$ mol) and stored at -80°C.

**Peptoid sequences and their UPLC-MS characterizations** (See Supplementary Figs 29 – 57).

Structures of the synthesized peptoids and molecular weight of each peptoid as determined by mass spectrometry are shown below.

Pep-**1**: 1965.6 (Molecular weight), 1966.4 (Found:$[M+H]^+$), 983.7 (Found:$[M/2+H]^+$). UPLC-MS spectra were shown in Supplementary Fig. 29.

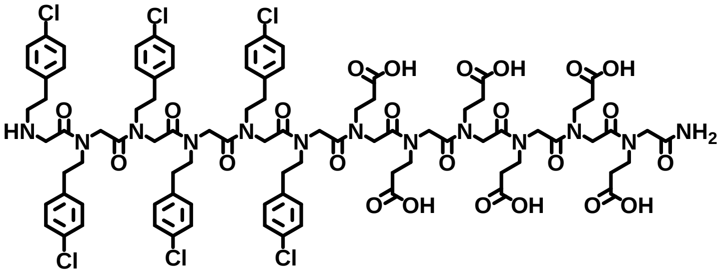


Pep-**2**: 1965.6 (Molecular weight), 983.6 (Found:$[M/2+H]^+$), 1966.3 (Found:$[M+H]^+$). UPLC-MS spectra were shown in Supplementary Fig. 30.

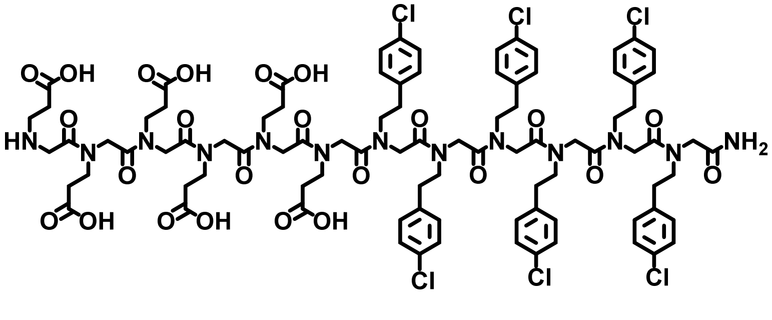

Pep-**3**: 2078.8 (Molecular weight), 2079.4 (Found:$[M+H]^+$), 1040.4 (Found: $[M/2+H]^+$). UPLC-MS spectra were shown in Supplementary Fig. 31.

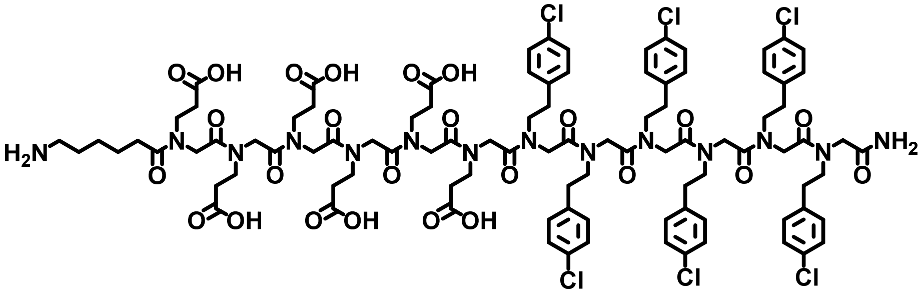

Pep-**4**: 2060.7 (Molecular weight), 2061.71 (Found:$[M+H]^+$), 1031.38 (Found:$[M/2+H]^+$). UPLC-MS spectra were shown in Supplementary Fig. 32.

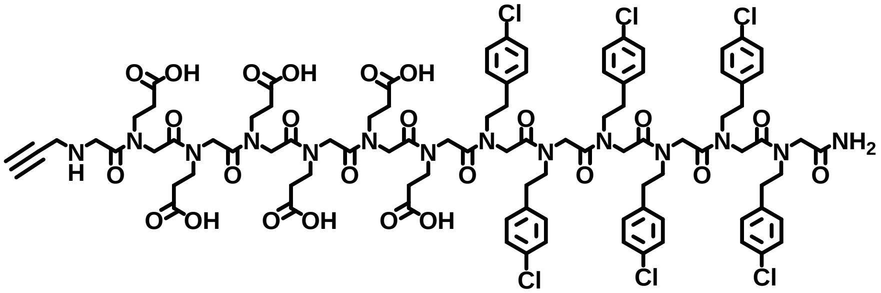

Pep-**5**: 2199.93 (Molecular weight), 2200.41 (Found:$[M+H]^+$), 1100.4 (Found: $[M/2+H]^+$). UPLC-MS spectra were shown in Supplementary Fig. 33.

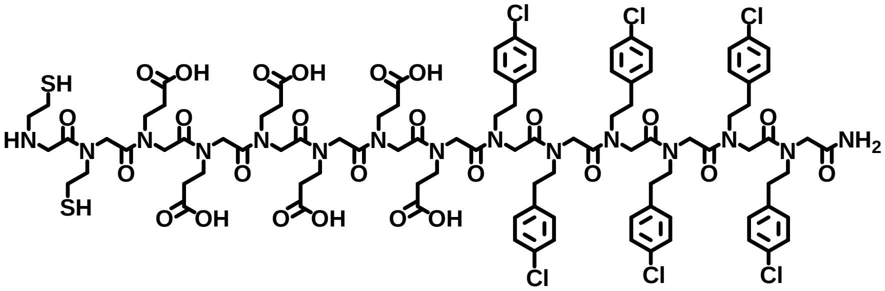

Pep-**6**: 1965.6 (Molecular weight), 1966.3 (Found:$[M+H]^+$), 983.4 (Found: $[M/2+H]^+$). UPLC-MS spectra were shown in Supplementary Fig. 34.

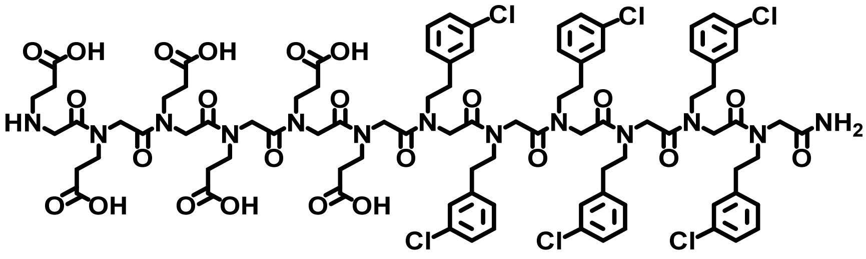

Pep-**7**: 1965.6 (Molecular weight), 1966.4 (Found:$[M+H]^+$), 983.6 (Found: $[M/2+H]^+$). UPLC-MS spectra were shown in Supplementary Fig. 35.

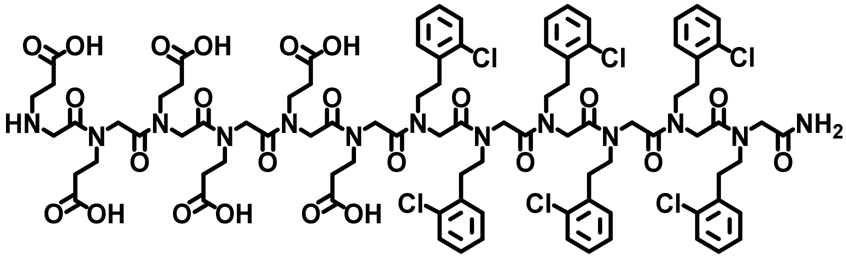

Pep-**8** **[**$(N_{4\text{-Cl}}pe)_3Nce_6$**]**: 1378.7 (Molecular weight), 1379.5 (Found:$[M+H]^+$). UPLC-MS spectra were shown in Supplementary Fig. 36.

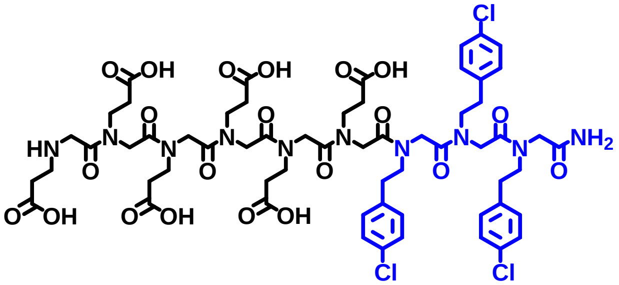

Pep-**9** **[**$(N_{4\text{-Cl}}pe)_6Nce_3$**]**: 1578.3 (Molecular weight), 1579.6 (Found:$[M+H]^+$). UPLC-MS spectra were shown in Supplementary Fig. 37.

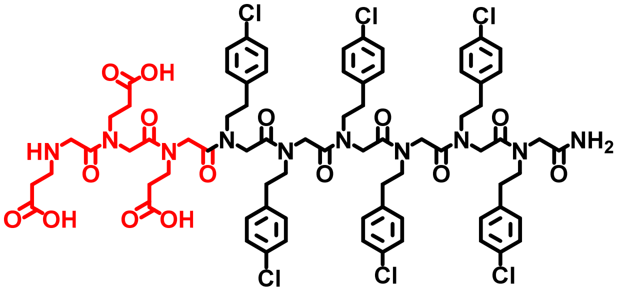

Pep-**10** **[**$(N_{4\text{-Cl}}pe)_6Nte_3$, Nte = N-2-(2-(2-methoxyethoxy)ethoxy)ethylglycine**]**: 1800.6 (Molecular weight), 1801.6 (Found:$[M+H]^+$), 901.8 (Found:$[M/2+H]^+$). UPLC-MS spectra were shown in Supplementary Fig. 38.

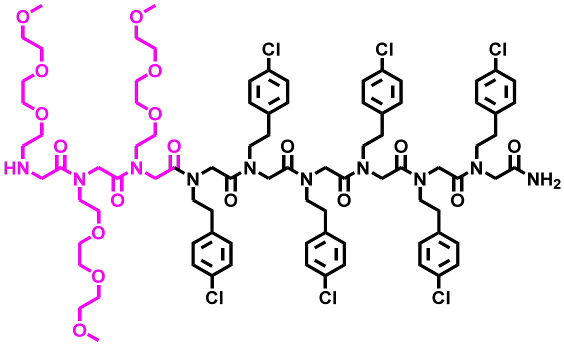

Pep-**11 [**(N$_{4\text{-Cl}}$pe)$_6$(NceNae)$_3$**]**: 1878.6 (Molecular weight), 1881.4 (Found:$[M+H]^+$), 940.1 (Found: $[M/2+H]^+$). UPLC-MS spectra were shown in Supplementary Fig. 39.

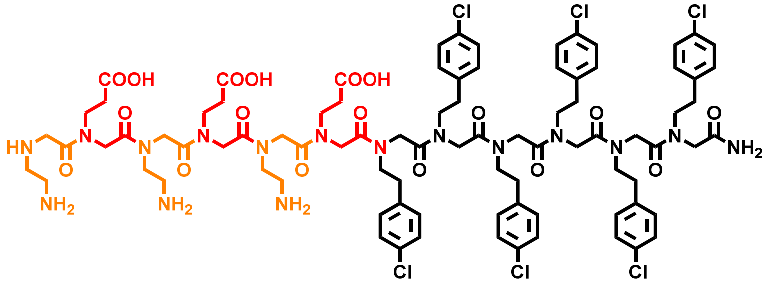

Pep-**12 [(Nhex)$_2$(N4-clpe)$_6$(Nce)$_6$]**: 2248.0 (Molecular weight), 2248.7 (Found:$[M+H]^+$), 1124.8 (Found: $[M/2+H]^+$). UPLC-MS spectra were shown in Supplementary Fig. 40.

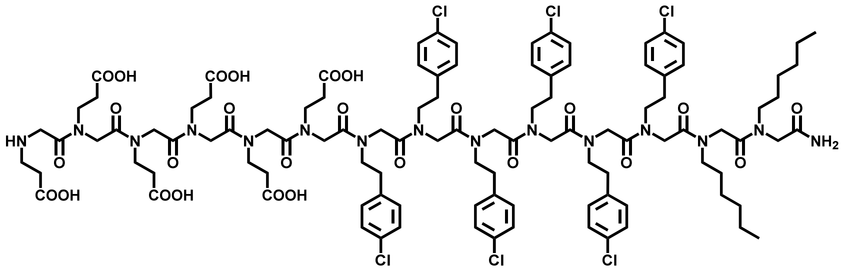

Pep-**13 [(Nhex)$_6$(Nce)$_6$]**: 1639.0 (Molecular weight), 1640.0 (Found:$[M+H]^+$). UPLC-MS spectra were shown in Supplementary Fig. 41.

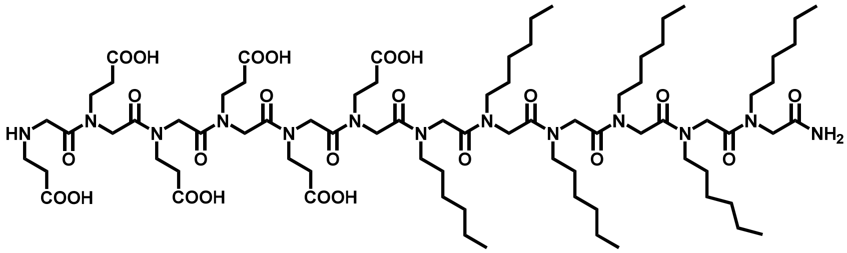

Pep-**14 [(Nhex)$_{12}$(Nce)$_6$]**: 2486.3 (Molecular weight), 2486.5 (Found:$[M]^+$), 1243.8 (Found: $[M/2]^+$). UPLC-MS spectra were shown in Supplementary Fig. 42.

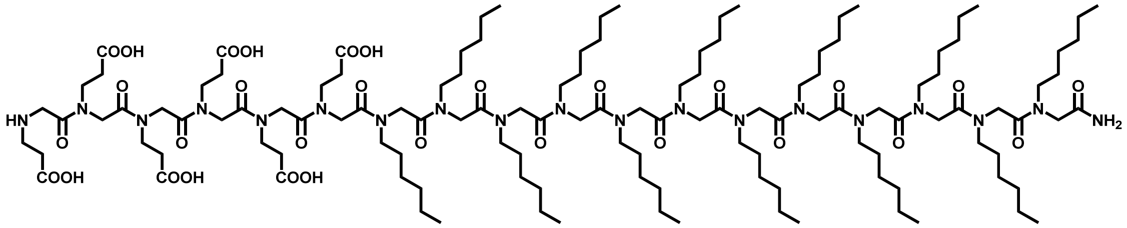

1-Ntyr-**Pep-2**: 2142.8 (Molecular weight), 2143.4 (Found:$[M+H]^+$), 1072.3 (Found:$[M/2+H]^+$). UPLC-MS spectra were shown in Supplementary Fig. 43.

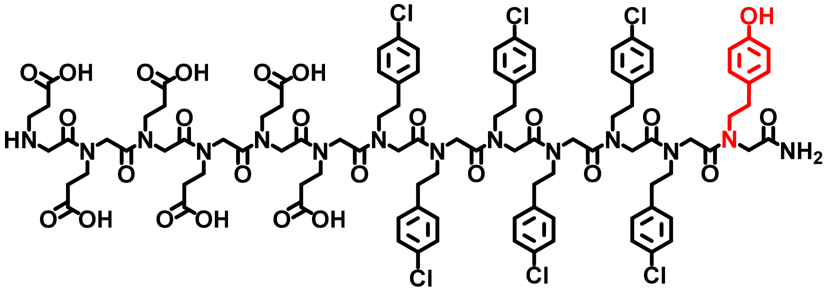

1-Npyr-**Pep-2**: 2236.92 (Molecular weight), 2237.70 (Found: $[M+H]^+$), 1119.59 (Found:$[M/2+H]^+$). UPLC-MS spectra were shown in Supplementary Fig. 44.

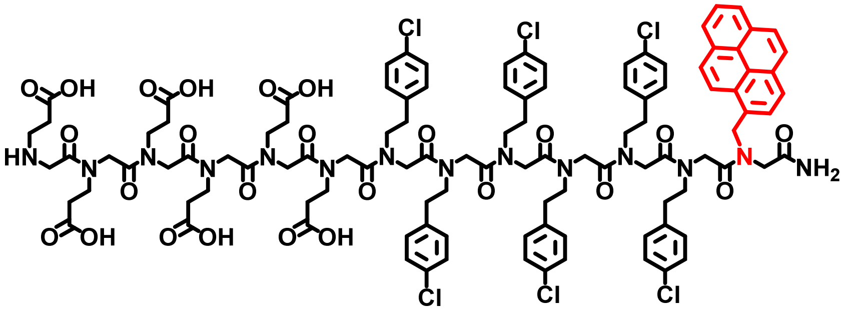

1-Nhex-**Pep-2**: 2106.81 (Molecular weight), 2108.15 (Found:$[M+H]^+$), 1054.25 (Found:$[M/2]^+$). UPLC-MS spectra were shown in Supplementary Fig. 45.

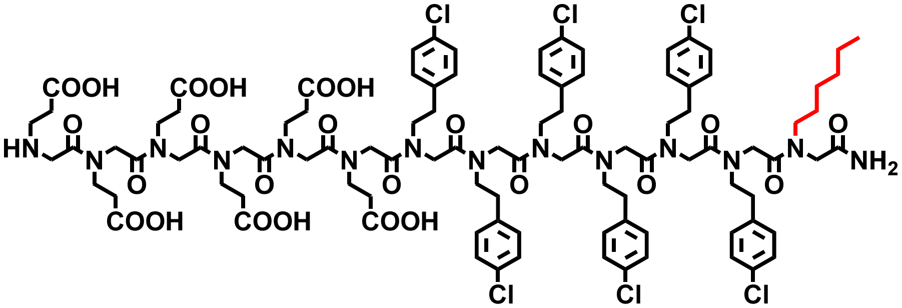

1,8-$(Nazo)_2$-**Pep-2**: 2240.12 (Molecular weight), 2440.22 (Found:$[M]^+$), 1221.0 (Found: $[M/2+H]^+$). UPLC-MS spectra were shown in Supplementary Fig. 46.

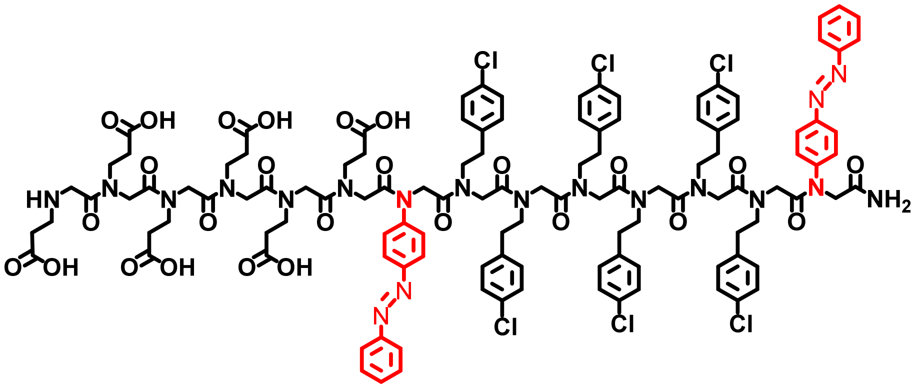

1,8-(Ntrp)$_2$-**Pep-2**: 2366.08 (Molecular weight), 2367.79 (Found:$[M+H]^+$), 1184.47 (Found: $[M/2+H]^+$). UPLC-MS spectra were shown in Supplementary Fig. 47.

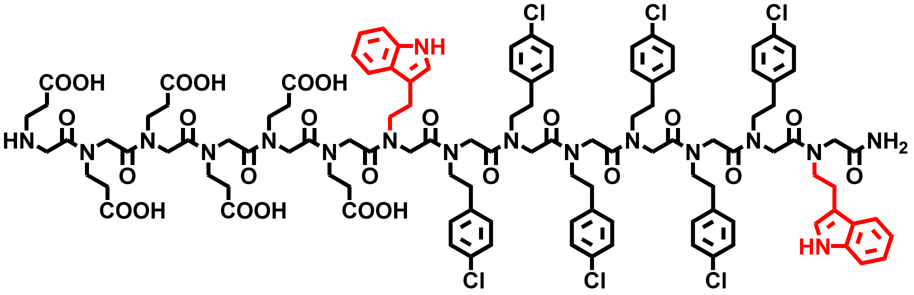

1,8-(Nse)$_2$-**Pep-2**: 2199.93 (Molecular weight), 2201.54 (Found:$[M+H]^+$), 1099.63 $[M/2+H]^+$). UPLC-MS spectra were shown in Supplementary Fig. 48.

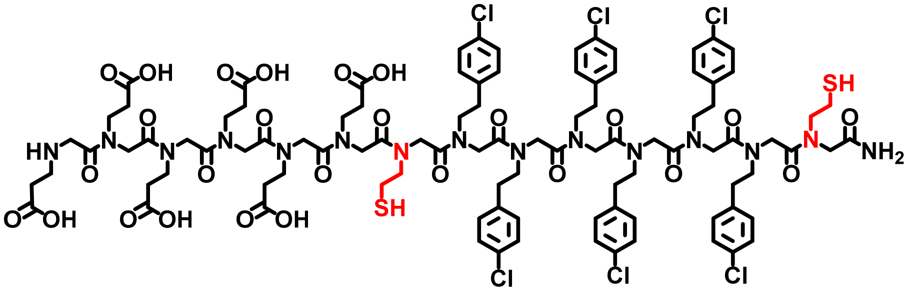

1,8-(Ntyr)$_2$-**Pep-2**: 2319.99 (Molecular weight), 2321.48 (Found:$[M+H]^+$), 1160.24 (Found:$[M/2]^+$). UPLC-MS spectra were shown in Supplementary Fig. 49.

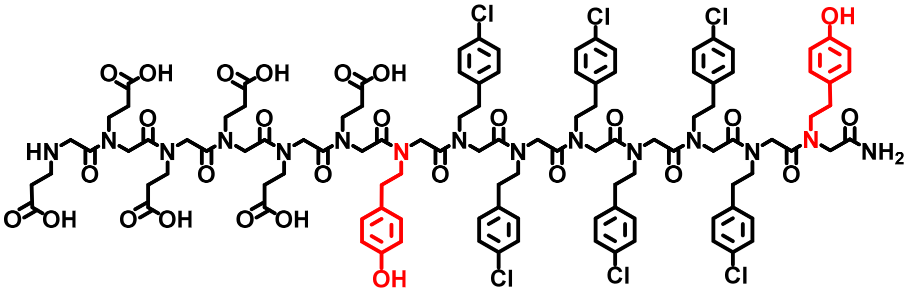

1-Nse-8-Ntyr-**Pep-2**: 2259.97 (Molecular weight), 2261.4 (Found:$[M+H]^+$), 1131.59 (Found: $[M/2+H]^+$). UPLC-MS spectra were shown in Supplementary Fig. 50.

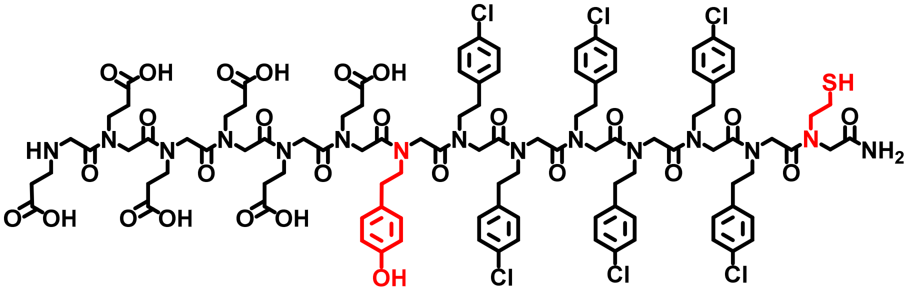

13-Nte-**Pep-2**: 2168.84 (Molecular weight), 2169.66 (Found:[M+H]$^{+}$), 1084.41 (Found: [M/2]$^{+}$). UPLC-MS spectra were shown in Supplementary Fig. 51.

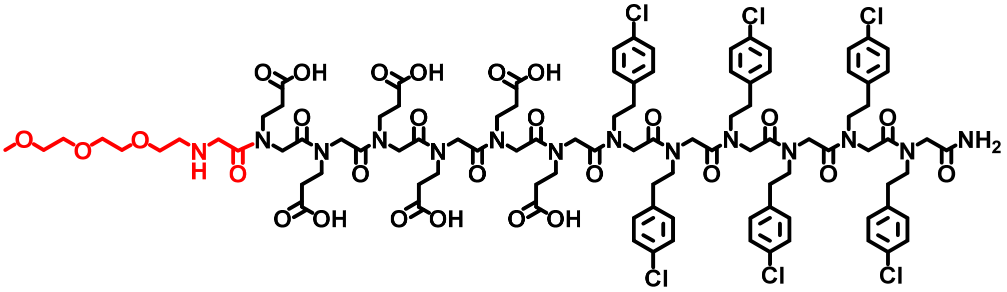

13-Nhis-**Pep-2**: 2116.77 (Molecular weight), 2116.77 (Found:[M]$^{+}$), 1058.75 (Found: [M/2]$^{+}$). UPLC-MS spectra were shown in Supplementary Fig. 52.

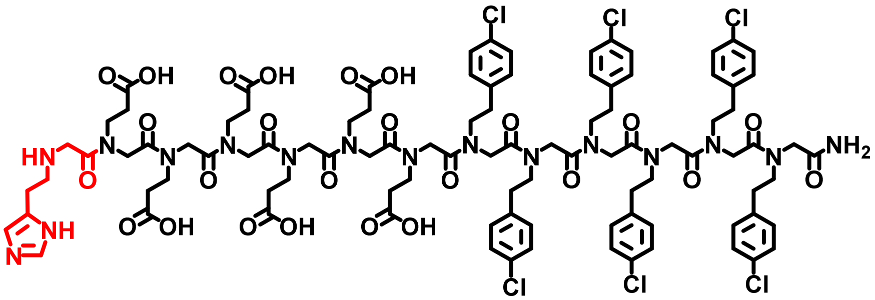

13-Ntyr-**Pep-2**: 2142.8 (Molecular weight), 2143.48 (Found:[M+H]$^{+}$), 1072.25 (Found: [M/2+H]$^{+}$). UPLC-MS spectra were shown in Supplementary Fig. 53.

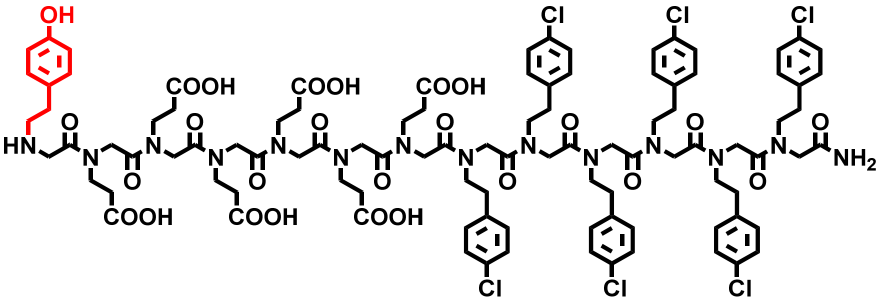

13-Nbce-**Pep-2**: 2288.94 (Molecular weight), 2289.23 (Found:[M]$^{+}$), 1144.64 (Found: [M/2]$^{+}$). UPLC-MS spectra were shown in Supplementary Fig. 54.

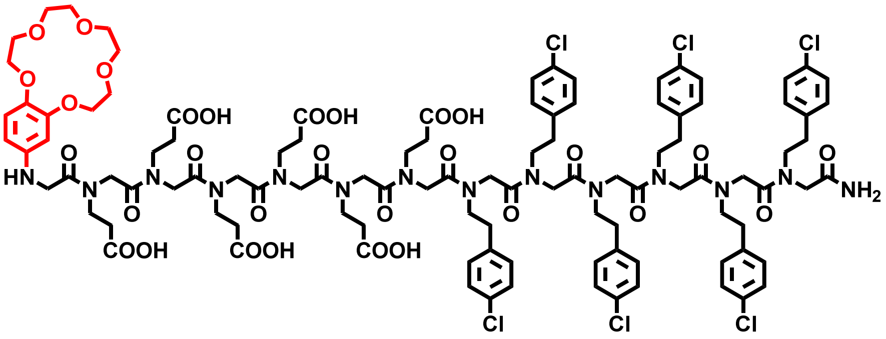

1,14-(Nse)$_2$-**Pep-2**: 2199.93 (Molecular weight), 2201.01 (Found:$[M+H]^+$), 1100.83 (Found: $[M/2+H]^+$). UPLC-MS spectra were shown in Supplementary Fig. 55.

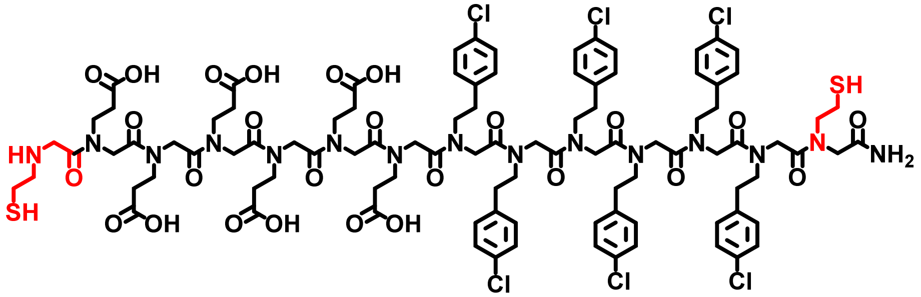

Ncd-**Pep-2**: 3139.6 (Molecular weight), 1571.1 (Found: $[M/2+H]^+$). UPLC-MS spectra were shown in Supplementary Fig. 56.

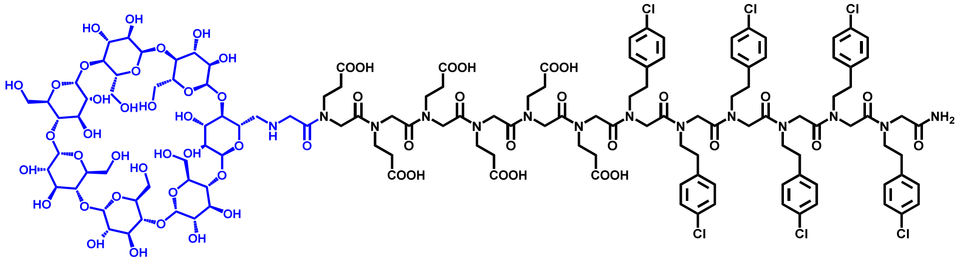

NHS-Rhodamine-labeled Pep-**3**: 2491.2 (Molecular weight), 2492.8 (Found:$[M+H]^+$), 1246.6 (Found: $[M/2+H]^+$). UPLC-MS spectra were shown in Supplementary Fig. 57.

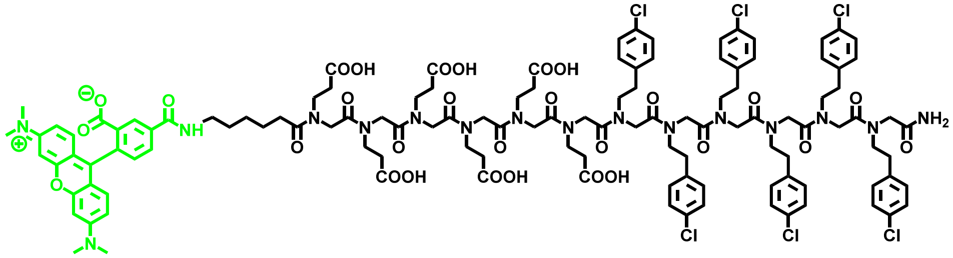

**Self-assembly of membrane-mimetic 2D nanomaterials from lipid-like peptoids**

Lyophilized and HPLC-grade peptoids were dissolved in the mixture of water and acetonitrile (v/v = 1:1) to make 5.0 mM clear solution, this clear solution was then transferred to 4 ºC refrigerator for slow evaporation. Suspensions or gel-like materials containing a large amount of crystalline membranes were formed after a few days.

For testing the influence of ionic strength in peptoid membrane formation, lyophilized Pep-**3** powders were dissolved in the mixture of water and acetonitrile (v/v = 1:1) with presence of NaCl (0.5 M) to make 5.0 mM clear solution for slow crystallization at 4 ºC.

For testing the change of solution pH in peptoid membrane formation, lyophilized peptoids were dissolved in the mixture of water and acetonitrile (v/v = 1:1) to make 5.0 mM clear solution, then the above solution pH was adjusted by adding 2.0 M NaOH

aqueous solution to a final pH value of 5.6, 7.4 or 10.50. This obtained clear solution was later used for slow evaporation in 4 ºC refrigerator. Suspensions or gel-like materials containing a large amount of crystalline membranes were formed after a few days.

For self-assembly in the mixture of PBS and CH3CN: Lyophilized peptoids were dissolved in the mixture of 1X PBS buffer and acetonitrile (v/v = 1:1) to make 5.0 mM clear solution, this clear solution was then transferred to 4 ºC refrigerator for slow evaporation. Suspensions or gel-like materials containing a large amount of crystalline membranes were formed after a few days.

**Characterizations of peptoid membranes**

Both *ex situ* (in air) and *in situ* (in fluid) AFM imaging were done in tapping mode or ScanAsyst mode at room temperature with a Bruker MultiMode 8. AFM samples were prepared by diluting peptoid membrane samples with water and using freshly cleaved mica as substrate. For stability test, pre-assembled Pep-**3** membranes were incubated with water in AFM fluid cell, *in situ* AFM images at different time points were collected. Pep-**3** stability in other solvents, such as pre-mixed water and acetonitrile (v/v = 1:1), ethanol, acetonitrile, aqueous solution with different salts and salt concentrations, was tested similarly by injected solvent into fluid cells or by fully exchanging these solvent with pre-injected waters in fluid cells. Similar protocols were used to prepare samples for salt-induced membrane thickness changes. For thermal stability test, gel-like materials of pre-assembled peptoid membranes in a 4.0 mL glass vial was placed in a 60 ºC oven overnight, and then used to prepare *ex situ* AFM samples by diluting them with water on freshly-cleaved mica surface. For the *in situ* formation of single-layer Pep-**3** membranes on mica surface: 2.0 mM aqueous solution of Pep-**3** were prepared by mixing lyophilized 3.0 µmol Pep-**3** in 1.5 mL water, and 10 µL 2.0 M NaOH was added to assist peptoid dissolution; 25 µL the resulting 2.0 mM Pep-**3** solution was diluted with 0.5 mL water and then well-mixed with 10 µL 0.1% TFA to get clear solution for *in situ* AFM studies. For Pep-**3** membrane repair experiment, pre-assembled Pep-**3** membranes were first deposited on mica surfaces and incubated with water. Selected membranes were purposely scratched to create defects using the AFM tip, and then aqueous solution of Pep-**3** (10 µM, pH 4.3) was injected into fluid cell for *in situ* membrane repair. For patterning Ncd-Pep-**2**, an aqueous solution of Ncd-Pep-**2** instead of Pep-**3** was used for repair of defects-containing Pep-**2** membranes.

TEM samples were prepared by pipetting one drop of water diluted peptoid membrane gels or suspensions onto carbon-coated electron microscopy grid; 2% phosphotungstic acid was then used for negative staining. TEM measurements were conducted on a 200-kV FEI Tecnai TEM microscope. SEM samples were prepared by pipetting one drop of water diluted peptoid membrane gels or suspensions onto silicon substrates. SEM measurements were performed on a FEI Helios Nanolab dual-beam focused ion beam/scanning electron microscopy (FIB/SEM) microscope. Powder X-ray diffraction data were collected at a multiple-wavelength anomalous diffraction and monochromatic macromolecular crystallography beamline, 8.3.1, at the Advanced Light Source located at Lawrence Berkeley National Laboratory. Beamline 8.3.1 has a 5 T single pole superbend

source with an energy range of 5 - 17 keV. Data were collected with a 3×3 CCD array (ADSC Q315r) detector at a wavelength of 1.1159 Å. Datasets were collected with the detector 200 mm from the sample. Peptoid membrane suspensions or pellets were pipetted onto a Kapton mesh (MiTeGen) and dry. Data was processed with custom Python scripts.

**Molecular Dynamics Simulations**

Since peptoid topologies and parameters are not present in standard MD forcefields, we generated them using the generalized amber forcefield (GAFF) and the Antechamber program.[7,8] The AM1-BCC model[9,10] was used to generate partial charges. GAFF parameters were converted into GROMACS-compatible topologies using the AcPype program,[11] and simulations were carried out using the AMBER03 forcefield[12] with the added peptoid parameters. GAFF-based peptoid models have been showed to correctly predict peptoid crystal structures.[13] In addition, a recently developed CHARMM-based model accurately predicts the free energy barrier between the *cis* and *trans* isomers of the peptoid backbone amide bond.[14]

Polymers were constructed in extended starting conformations with all-*trans* backbone amides using an in-house python script. While *cis* backbone amides are important in structures like the peptoid polyproline I helix[15] and a cyclic peptoid nonamer,[13] the *trans* configuration is preferred by about 3:1 for peptoids without branched sidechains.[16] In addition, the barrier for *cis*/*trans* isomerization in peptoids is about 29*k$T$,[17] which is beyond the thermal motions that can be sampled on the simulation timescale. Furthermore, peptoid assembly into extended planar nanostructures like the peptoid nanosheet and membrane requires the linear, untwisted Σ-strand backbone conformation,[18] which only forms with all-*trans* backbone amides.

We start a variety of configurations with half the peptoids oriented up along the z-axis and half oriented down. The hydrophobic region ($N_{4\text{-Clpe}}$ residues) is placed in the center to minimize exposed hydrophobic surface area. The sidechains are oriented along the y-axis, and the peptoids are stacked along the x-axis. Various system sizes were tried including 8 x 4, 16 x 4, and 16 x 6, and each system is solvated with a 2-nm layer of water in the z direction.

After a steepest-descent minimization of the starting structure, the system was gradually heated from 0 to 300K in 50K increments, with 30 ps at each temperature followed by 100 ps of relaxation at 300 K. MD simulations were performed with GROMACS 4.6.4 [19]. We used 1.0-nm cutoffs for van der Waals and Coulombic interactions and the particle-mesh Ewald method [20] for long-range electrostatic interactions. The simulations were performed in the NPT ensemble with a Parrinello-Rahman barostat [21] with a 1 ps coupling time at 1 bar and anisotropic pressure coupling. A Nose-Hoover thermostat [22] at 300 K with 0.2 ps coupling time was applied separately to the polymer and to the solution (including ions). Simulations were performed for 500 to 1000 ns using a timestep of 2 fs.

**X-ray diffraction calculations**

We calculate X-ray (powder) diffraction from simulation structures according to the formula

$$I(\mathbf{q}) = \left|\sum_{j=1}^{N} f_j(q)\exp(i\mathbf{q}\cdot\mathbf{r}_j)\right|, \quad (1)$$

where the sum is over all atoms, $f_j(q)$ is the Cromer-Mann scattering factor for atom $j$ at magnitude $q$ &, and $\mathbf{r}_j$ are the coordinates of atom $j$. We choose $N_q = N_x*N_y*N_z$. $N_d = b_d/dr$ rounded to the nearest integer, where $L_d$ is the length of the box vector in dimension d, and $dr$ is the (real space) grid spacing, which we set to 0.5 Å. For each triplet in i,j,k space, the corresponding $\mathbf{q}$ vector is $2\pi*(i/L_x,j/L_y,k/L_z)$. Since computed intensity scales with the square of the system size, we normalize the computed intensity by the square of the number of peptoid atoms.

In addition, to facilitate identification of the most important peaks for comparison between computation and experiment, we smoothed the computed XRD using a Gaussian function. For each reflection in each structure, we distribute the intensity over $q$ space using a Gaussian

$$I(q) = \frac{I_0(q_0)}{\sigma\sqrt{2\pi}}\exp\frac{(q-q_0)^2}{2\sigma^2}, \quad (2)$$

where $I_0$ and $q_0$ are the intensity and $q$ (magnitude) of the original reflection, respectively. We choose σ so that 90% of the probability density falls within 0.025 $Å^{-1}$ of $q_0$, which leads to $\sigma = 0.0152$ $Å^{-1}$.

## **Supplementary References**


1 Guo, W. H., Li, J. J., Wang, Y. A. & Peng, X. G. Luminescent CdSe/CdS core/shell nanocrystals in dendron boxes: Superior chemical, photochemical and thermal stability. *J. Am. Chem. Soc.* **125**, 3901-3909 (2003).

2 Sun, J., Stone, G. M., Balsara, N. P. & Zuckermann, R. N. Structure-conductivity relationship for peptoid-based PEO-mimetic polymer electrolytes. *Macromolecules* **45**, 5151-5156 (2012).

3 Petter, R. C., Salek, J. S., Sikorski, C. T., Kumaravel, G. & Lin, F. T. Cooperative binding by aggregated mono-6-(alkylamino)-beta-cyclodextrins. *J. Am. Chem. Soc.* **112**, 3860-3868 (1990).

4 Chen, C.-L., Qi, J., Tao, J., Zuckermann, R. N. & DeYoreo, J. J. Tuning calcite morphology and growth acceleration by a rational design of highly stable protein-mimetics. *Sci. Rep.* **4** (2014).

5 Zuckermann, R. N., Kerr, J. M., Kent, S. B. H. & Moos, W. H. Efficient method for the preparation of peptoids oligo(N-substituted glycines) by submonomer solid-phase synthesis. *J. Am. Chem. Soc.* **114**, 10646-10647 (1992).

6 Burkoth, T. S., Fafarman, A. T., Charych, D. H., Connolly, M. D. & Zuckermann, R. N. Incorporation of unprotected heterocyclic side chains into peptoid oligomers via solid-phase submonomer synthesis. *J. Am. Chem. Soc.* **125**, 8841-8845 (2003).

7 Wang, J., Wang, W., Kollman, P. A. & Case, D. A. Automatic atom type and bond type perception in molecular mechanical calculations. *J. Mol. Graph. Model.* **25**, 247-260 (2006).

8 Wang, J. M., Wolf, R. M., Caldwell, J. W., Kollman, P. A. & Case, D. A. Development and testing of a general amber force field. *J. Comput. Chem.* **25**, 1157-1174 (2004).

9 Jakalian, A., Bush, B. L., Jack, D. B. & Bayly, C. I. Fast, efficient generation of high-quality atomic Charges. AM1-BCC model: I. Method. *J. Comput. Chem.* **21**, 132-146 (2000).

10 Jakalian, A., Jack, D. B. & Bayly, C. I. Fast, efficient generation of high-quality atomic charges. AM1-BCC model: II. Parameterization and validation. *J. Comput. Chem.* **23**, 1623-1641 (2002).

11 Sousa da Silva, A. & Vranken, W. ACPYPE - AnteChamber PYthon Parser interfacE. *BMC Res Notes* **5**, 1-8 (2012).

12 Duan, Y. *et al.* A point-charge force field for molecular mechanics simulations of proteins based on condensed-phase quantum mechanical calculations. *J. Comput. Chem.* **24**, 1999-2012 (2003).

13 Butterfoss, G. L. *et al.* De novo structure prediction and experimental characterization of folded peptoid oligomers. *Proc. Natl. Acad. Sci. U. S. A.* **109**, 14320-14325 (2012).

14 Mirijanian, D. T., Mannige, R. V., Zuckermann, R. N. & Whitelam, S. Development and use of an atomistic CHARMM-based forcefield for peptoid simulation. *J. Comput. Chem.* **35**, 360-370 (2014).

15 Wu, C. W., Sanborn, T. J., Huang, K., Zuckermann, R. N. & Barron, A. E. Peptoid oligomers with α-chiral, aromatic side chains: Sequence requirements for the formation of stable peptoid helices. *J. Am. Chem. Soc.* **123**, 6778-6784 (2001).

16 Sui, Q., Borchardt, D. & Rabenstein, D. L. Kinetics and equilibria of cis/trans isomerization of backbone amide bonds in peptoids. *J. Am. Chem. Soc.* **129**, 12042-12048 (2007).

17 Gasparro, F. P. & Kolodny, N. H. NMR determination of the rotational barrier in N,N-dimethylacetamide. A physical chemistry experiment. *J. Chem. Edu.* **54**, 258 (1977).

18 Mannige, R. V. *et al.* Peptoid nanosheets exhibit a new secondary-structure motif. *Nature* **526**, 415-420 (2015).

19 Pronk, S. *et al.* GROMACS 4.5: a high-throughput and highly parallel open source molecular simulation toolkit. *Bioinformatics* **29**, 845-854 (2013).

20 Darden, T., York, D. & Pedersen, L. Particle mesh ewald - an n.log(n) method for ewald sums in large systems. *J. Chem. Phys.* **98**, 10089-10092 (1993).

21 Parrinello, M. & Rahman, A. polymorphic transitions in single-crystals - a new molecular-dynamics method. *J. Appl. Phys.* **52**, 7182-7190 (1981).

22 Hoover, W. G. Canonical dynamics - equilibrium phase-space distributions. *Phys. Rev. A* **31**, 1695-1697 (1985).
